# Identifying QTLs involved in hybrid performance and heterotic group complementarity: new GWAS models applied to factorial and admixed diallel maize hybrid panels

**DOI:** 10.1007/s00122-023-04431-w

**Published:** 2023-10-10

**Authors:** Aurélien Beugnot, Tristan Mary-Huard, Cyril Bauland, Valerie Combes, Delphine Madur, Bernard Lagardère, Carine Palaffre, Alain Charcosset, Laurence Moreau, Julie B. Fievet

**Affiliations:** 1 Université Paris-Saclay, INRAE, CNRS, AgroParisTech, UMR GQE-Le Moulon, 91272 Gif-Sur-Yvette, France; 2 Université Paris-Saclay, AgroParisTech, INRAE, UMR MIA Paris-Saclay, 91120 Palaiseau, France; 3 INRAE, UE 0394, 40390 Saint Martin De Hinx, France

## Abstract

**Key message:**

An original GWAS model integrating the ancestry of alleles was proposed and allowed the detection of background specific additive and dominance QTLs involved in heterotic group complementarity and hybrid performance.

**Abstract:**

Maize genetic diversity is structured into genetic groups selected and improved relative to each other. This process increases group complementarity and differentiation over time and ensures that the hybrids produced from inter-group crosses exhibit high performances and heterosis. To identify loci involved in hybrid performance and heterotic group complementarity, we introduced an original association study model that disentangles allelic effects from the heterotic group origin of the alleles and compared it with a conventional additive/dominance model. This new model was applied on a factorial between Dent and Flint lines and a diallel between Dent-Flint admixed lines with two different layers of analysis: within each environment and in a multiple-environment context. We identified several strong additive QTLs for all traits, including some well-known additive QTLs for flowering time (in the region of Vgt1/2 on chromosome 8). Yield trait displayed significant non-additive effects in the diallel panel. Most of the detected Yield QTLs exhibited overdominance or, more likely, pseudo-overdominance effects. Apparent overdominance at these QTLs contributed to a part of the genetic group complementarity. The comparison between environments revealed a higher stability of additive QTL effects than non-additive ones. Several QTLs showed variations of effects according to the local heterotic group origin. We also revealed large chromosomic regions that display genetic group origin effects. Altogether, our results illustrate how admixed panels combined with dedicated GWAS modeling allow the identification of new QTLs that could not be revealed by a classical hybrid panel analyzed with traditional modeling.

**Supplementary Information:**

The online version contains supplementary material available at 10.1007/s00122-023-04431-w.

## Introduction

The phenotypic superiority of a hybrid relative to its progenitors was first observed by Darwin (1876), described on maize by East and Shull (East 1908; Shull 1908) and then named hybrid vigor or heterosis by Shull (1914). This empirical observation prompted the invention of hybrid varieties as a mean to propagate a superior genotype in the presence of inbreeding depression (Shull 1908). Three non-exclusive genetic mechanisms can explain heterosis: dominance, overdominance and epistasis (Birchler et al. 2010). At the biological level, dominance at a locus is defined as the deviation between the value of heterozygous genotypes and the average value of the two homozygous ones. Only two loci with dominant favorable alleles carried each by a different parent are enough to generate heterosis (Davenport 1908; Bruce 1910; Jones 1917). Overdominance is the advantage of the heterozygous genotype relative to the best homozygous genotype (Hull 1946). In this case, a single locus can explain the hybrid advantage (Hull 1945; Crow 1948). Importantly, two or more tightly linked loci with favorable dominant and unfavorable recessive alleles in repulsion mimic overdominance in the corresponding genomic region, a phenomenon referred to as pseudo-overdominance (Jones 1917; Graham et al. 1997; Crow 1999). Epistasis can also contribute to heterosis due to positive interaction effects between alleles at different loci inherited from different parents (Richey 1942; Powers 1944; Jinks and Jones 1958; Williams 1960). The relative contribution of these mechanisms remains subject to debate (Lamkey and Edwards 1999).

Hybrid breeders progressively structured the parental lines into heterotic groups to avoid evaluating crosses between closely related inbred lines. This process prevents inbreeding depression and therefore maximizes hybrid performance. These groups were developed and improved in parallel based on the performances of the inter-group hybrids, following the principle of reciprocal recurrent selection (Hull 1945; Melchinger and Gumber 1998). Groups diverged due to both the genetic drift and selection processes (Lamkey and Lorenz 2014; Gerke et al. 2015), leading to different intra-group allelic frequencies. Hence, crossing parental lines selected from divergent groups to produce hybrids is particularly interesting since it warrants the assembly of the favorable alleles fixed in each group and a high degree of heterozygosity of the hybrids preventing inbreeding depression (Reif et al. 2007). As an example, maize Northern European breeding programs are based on two main heterotic groups: (i) the Dent group composed of several heterotic American groups (mostly Iowa Stiff Stalk Synthetic, Non-Stiff Stalk and Iodent) originating from maize selection in the US corn belt and (ii) the Flint group gathering European lines originally derived from local landraces (Tenaillon and Charcosset 2011; Brandenburg et al. 2017). Identifying the loci involved in the inter-group hybrid performance and the complementarity between heterotic groups is of main interest to optimize hybrid breeding programs.

Differences in QTL effects between ancestry groups were reported in human populations (Williams et al. 2000) with variable amplitude, up to opposite sign effects (Deng 2001). Meta-analysis (Evangelou and Ioannidis 2013; Li and Keating 2014) was used to investigate the variation of effects across human ancestry groups (Ioannidis et al. 2004; Waters et al. 2010; Marigorta and Navarro 2013) and to point out interactions between alleles and group origins (Ntzani et al. 2012; Wyss et al. 2018). In maize, within-group effects were highlighted for the phenology (Buckler et al. 2009; Durand et al. 2012; Giraud et al. 2014; Rio et al. 2020). The possible reasons for these allelic effect variations according to the origin include: (i) differences in polymorphism, (ii) epistatic effects between the causal QTLs and the genetic background (Rio et al. 2020), (iii) differences in local linkage disequilibrium (LD) patterns between SNPs and causal QTLs.

To study the implication of these factors in the heterotic group complementarity, it is necessary to disentangle the allelic effects from those of the group origin. To solve this issue, Rio et al. 2020 suggested using admixed lines derived from inter-group crosses, the genome of which is composed of a patchwork of chromosomic segments of different ancestries. They proposed a new GWAS model that included both the SNP allele and its group origin and applied it on an admixed inbred line panel evaluated per se for traits related to plant architecture and phenology. This strategy made it possible to detect QTLs showing different effects depending on the group origin.

Here, we propose to extend this approach by evaluating an admixed diallel hybrid panel to investigate the loci involved in hybrid performances and, more particularly, in heterotic group complementarity. To this end, we developed a generic hybrid GWAS model accounting for allele-based and ancestry-based effects in the same model to (i) detect additive and dominance QTL involved in hybrid performances, (ii) highlight loci in which allelic effects depend on the heterotic group they originate from and (iii) reveal the existence of ancestry effects, beyond the allelic state at individual SNPs. We used two different hybrid panels to test this approach: a Dent-Flint factorial panel and a diallel panel issued from admixed Dent-Flint inbred lines. This method was applied to performances measured in several trials to evaluate the co-location of QTLs through environmental variations and for different traits involved in phenology (anthesis and silking dates), grain moisture and grain yield. We benchmarked our approach with a classical GWAS model not accounting for the allele origin effects. We discussed the interest in admixed hybrid panels breaking the structure between heterotic groups for identifying loci involved in heterotic group complementarity, whose effects are hidden by the genetic structure when using a factorial hybrid panel.

## Material and methods

### Plant material

**Fig. 1:**
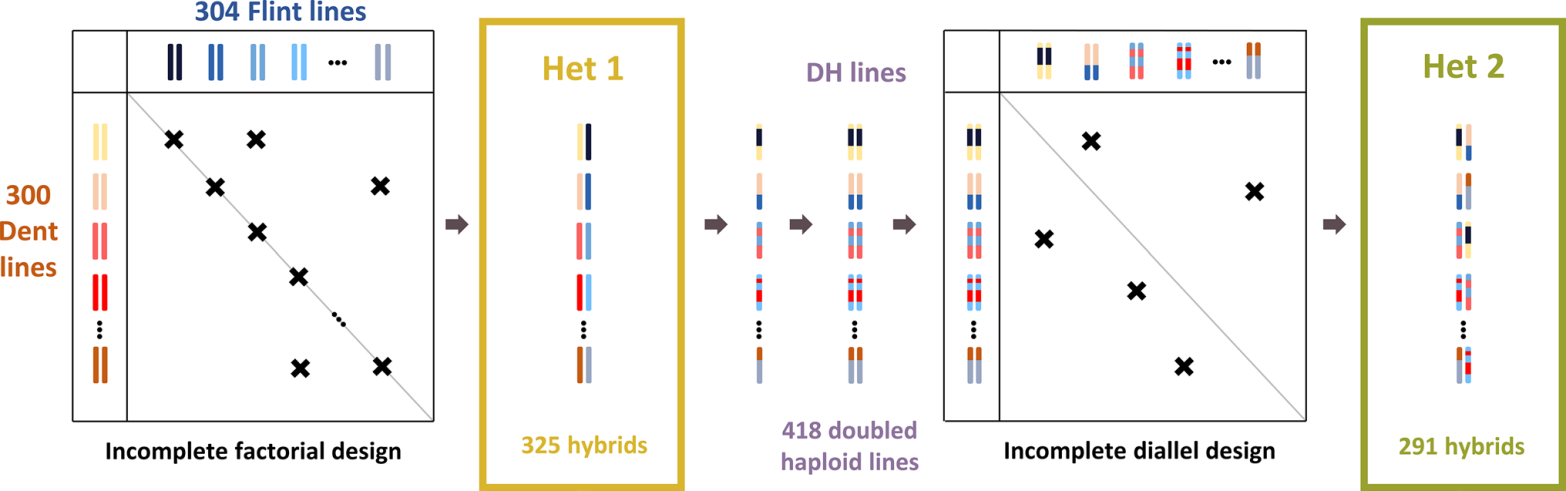
Diagram of Het1 and Het2 hybrid panel production. The 325 Het1 hybrids were produced by crossing Dent and Flint lines, following a sparse factorial design. DH lines were generated from these hybrids and crossed following a sparse diallel design to produce the 291 Het2 admixed hybrids

In this study, two hybrid panels were developed following the procedure described in Fig. 1.

*Het1 panel* 304 Flint inbred lines and 300 Dent inbred lines were collected from the Flint panel (Rincent et al. 2014a, b) and the Dent panel (Rio et al. 2019), in order to represent the maize diversity used in North European selection programs. Lines within each group were selected to cover different breeding generations. These Dent and Flint lines were crossed following an incomplete factorial design to produce 325 single-cross hybrids. The resulting hybrid genomes were 50% percent Dent and 50% Flint, with one allele of each origin at all loci. Each line contributed to 1 to 5 hybrids (1.2 on average). Het1 was evaluated in seven environments over two years (3 in 2014 and 4 in 2015) at six different locations in France (Aubiat AUB, Caussade CAU, Morlaas MOR, Saint-Martin-de-Hinx SMH, Rhodon RHO, Villampuy VIL). Each environment consisted of a single field trial of 512 two-row plots of 9.28 m^2^ each. Within each environment, hybrids were separated within the field into two blocks regarding their precocity to avoid competition. One block included the group of early and intermediate flowering genotypes (which flowered between 201 and 212 days after January 1st), and the second one was composed of the group of intermediate and late flowering genotypes (flowering time between 212.5 and 228 days after January 1st).

*Het2 panel* 321 admixed lines between the two heterotic group origins were generated from 202 Het1 hybrids (among the 325) using doubled haploidization method (Bordes et al. 1997) and are described in Rio et al. (2019). Admixed DH lines were crossed following a sparse diallel design leading to 291 hybrids. Each admixed line contributed to 1 to 4 hybrids (1.8 on average). At a given locus, Het2 admixed hybrids carry alleles from different group origins and can therefore be homozygote Dent, homozygote Flint or heterozygote Dent/Flint. The Het2 panel was phenotyped at 4 locations (Aubiat AUB, Jargeau JAR, Saint-Martin-de-Hinx SMH, Souprosse SOU) over two years (2 in 2016 and 3 in 2017), which resulted in 5 different environments (JAR16, AUB17, SOU17, SNH16, SMH17, see Roth et al. (2022) for details). Each environment consisted of a single field trial of 512 two-row plots of 9.28 m^2^ each.

For both panels, the same 4 hybrids (DKC4841, B73 x UH007, Millesim and PH207 x UH007) were used as checks and were repeated in all environments (3.8 times on average for Het1 and 2.9 times for Het2).

### Plant phenotyping and field data correction

Each panel was phenotyped for four traits: two phenological stages (male and female flowering time, called FloM and FloF, respectively, in days between the 1st of January corresponding to the date at which half of the plants of a plot exhibited visible tassels or silks, respectively) and two traits associated with yield (grain moisture (HUM) in percentage of fresh weight and grain yield (GY) at 15% grain moisture at harvest in q/ha). Several plots were discarded following experimenter recommendations and after filtering plots based on plant density (performances of plots with a number of plants lower than the median minus 15 plants were discarded). Outlier observations were then filtered out. The percentage of discarded observations for outliers represented 2% (on average across environments and traits) for Het1 and 1% for Het2 of the total observations retained by field experimenters. In a given environment, each hybrid was evaluated 1.1 times on average.

Local environmental effects were corrected within each environment using a model adapted to each panel. The following model was applied on Het1:

$$\begin{gathered} Y_{{{\text{htfrc}}}} = \mu + \lambda _{{\text{t}}} + \delta _{{\text{f}}} + R_{{\text{r}}} + C_{{\text{c}}} + G_{{\text{h}}} + E_{{{\text{htfrc}}}} \hfill \\ R\sim N\left( {0,I\sigma _{{\text{R}}}^{2} } \right) \hfill \\ C\sim N\left( {0,I\sigma _{{\text{C}}}^{2} } \right) \hfill \\ G\sim N\left( {0,I\sigma _{{\text{G}}}^{2} } \right) \hfill \\ E\sim N\left( {0,I\sigma _{\varepsilon }^{2} } \right) \hfill \\ \quad \quad R \bot C \bot G \bot E \hfill \\ \end{gathered}$$

where ${Y}_{\mathrm{htfrc}}$ is the phenotype of hybrid *h* with check status *t* in the precocity group *f*, at row *r* and column *c*, $\mu$ is the intercept, ${\lambda }_{\mathrm{t}}$ is the effect of check t (t has 5 levels, one for each of the 4 checks and 1 for experimental hybrids), ${\delta }_{f}$ is the effect of the precocity bloc *f*, ${R}_{\mathrm{R}}$ the random effect of plot row *r* with ${\sigma }_{R}^{2}$ variance and ${C}_{\mathrm{c}}$ the random effect of plot column *c* with ${\sigma }_{C}^{2}$ variance, ${G}_{h}$ is the random effect of genotype *h* with ${\sigma }_{G}^{2}$ the genetic variance and ${E}_{htfrc}$ is the error with ${\sigma }_{\varepsilon }^{2}$ the error variance. The sign $\perp$ indicates independence between random effects. Row and column effects were predicted using best linear unbiased prediction (BLUP). Corrected phenotypes (further referred to as phenotypes in the next sections) were obtained by subtraction of these predicted values from phenotypic values. An equivalent model without the precocity group effect was applied to the Het2 panel (See Appendix S1). The choice for each trait and each trial of a “row-column” model to correct for spatial heterogeneities was based on AIC/BIC criteria. As Het1 and Het2 were not evaluated in the same trials, they cannot be directly compared. In order to compare them, for each trait, we used the average corrected phenotypes of the checks that were evaluated in both Het1 and Het2 field trials to estimate the environmental difference between the two trial networks and we adjusted the mean performances of Het1 and Het2 for this difference. From this point, checks were removed from the analyses described below. For each trait, the corrected hybrid phenotypes were used to compute broad sense heritability within each environment and globally across environments (Appendix S2). To do so, we considered simple models including a random genetic effect for single-environment analyses plus a fixed environment and random genotype-by-environment effects for the multi-environment analysis.

### Genotyping

Dent and Flint lines were genotyped with a 600K SNPs Affymetrix Maize Genotyping array (Unterseer et al. 2014). Heterozygous and missing data were imputed in each group separately using BEAGLE v.3.3.2 (Browning and Browning 2009) by (Rio et al. 2019). Admixed lines were genotyped using a private 15K SNPs array provided by Limagrain. This array included a subset of the 50K Illumina MaizeSNP50 BeadChip loci (Ganal et al. 2011). From this genotyping information, the positions of recombination events were identified, and the different chromosomal segments carried by the admixed lines were assigned to one of the heterotic group origins (Flint, noted F, or Dent, noted D). DH lines were imputed up to 600K SNPs based on information on adjacent SNPs obtained from the 15K SNPs chip (Rio et al. 2019) and their parental lines 600K SNPs genotypes (Dent and Flint lines). Genotypes of single-cross hybrids of Het1 and Het2 panels were reconstructed from their respective parental genotypic data. In each panel, markers with Minor Allele Frequencies (MAF) < 5% were discarded. This resulted in a total of 482,078 markers for Het1 and 481,843 markers for Het2.

**Fig. 2:**
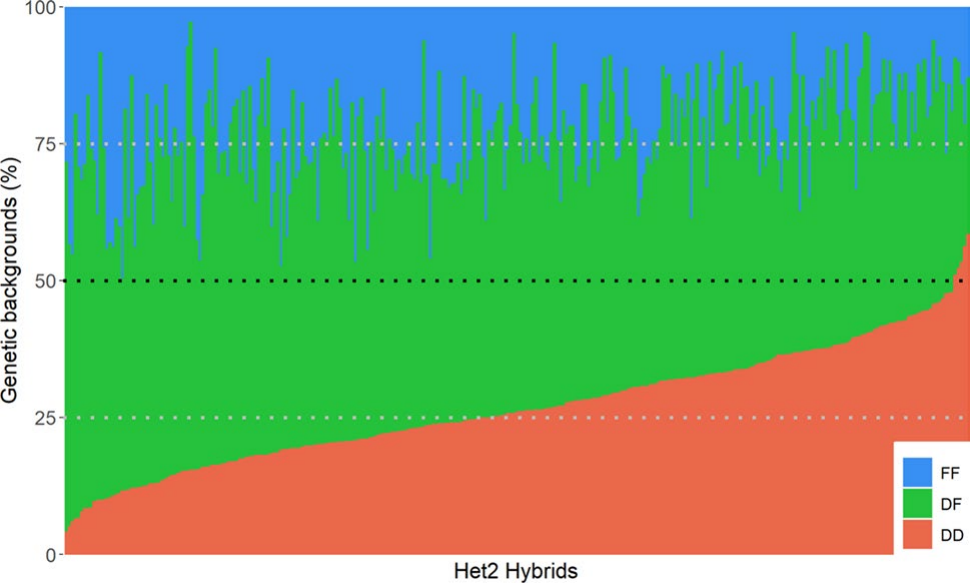
Repartition of genetic background proportions (DD, DF and FF) in the Het2 hybrids. Proportions are calculated based on physical distances (Mbp)

Het2 hybrids showed a wide range of admixture degrees between heterotic groups (Fig. 2). On average, they were constituted of 27% of loci with both alleles originating from the Dent group (DD genetic background, ranging from 4 to 58%), 23% of loci with both alleles from the Flint group (FF genetic background, ranging from 3 to 50%) and 50% of loci with one allele from each heterotic group (DF genetic background, ranging from 22 to 88%) which is close to the expected values (25%, 25% and 50%, respectively).

## Methods

### Variance partition

Before performing GWAS, variance decomposition was done to estimate the proportion of additive and non-additive genetic variance components. We used a model proposed by Vitezica et al. (2017) to partition hybrid genetic variance into additivity, dominance or epistatic deviation terms. This method is an extension of the Natural and orthogonal interaction approach (Álvarez-Castro and Carlborg 2007) to the estimation of covariances between individuals through kinship matrices (see below for their computation).

The following model was applied to each environment separately (single-environment model or MONO model):

$$\begin{gathered} Y_{{{\text{hr}}}} = \mu + A_{{\text{h}}} + D_{{\text{h}}} + I_{{\text{h}}}^{{{\text{aa}}}} + I_{{\text{h}}}^{{{\text{ad}}}} + I_{{\text{h}}}^{{{\text{dd}}}} + E_{{{\text{hr}}}} \hfill \\ A\sim N\left( {0,{\text{ K}}_{{\text{a}}} \sigma_{{\text{a}}}^{2} } \right), D\sim N\left( {0,{\text{ K}}_{{\text{d}}} \sigma_{{\text{d}}}^{2} } \right) \hfill \\ I^{{{\text{aa}}}} \sim N\left( {0,{\text{ K}}_{{{\text{aa}}}} \sigma_{{{\text{aa}}}}^{2} } \right), I^{{{\text{ad}}}} \sim N\left( {0,{\text{ K}}_{{{\text{ad}}}} \sigma_{{{\text{ad}}}}^{2} } \right), I^{{{\text{dd}}}} \sim N\left( {0,{\text{ K}}_{{{\text{dd}}}} \sigma_{{{\text{dd}}}}^{2} } \right) \hfill \\ E\sim N\left( {0,{ }I\sigma_{\varepsilon }^{2} } \right) \hfill \\ A\; \bot D\; \bot I^{{{\text{aa}}}} \;\bot I^{{{\text{ad}}}}\; \bot I^{{{\text{dd}}}} \bot\; E \hfill \\ \end{gathered}$$

where ${Y}_{hr}$ is the phenotype of the replicate *r* of hybrid *h* and $\mu$ is the intercept, ${A}_{\mathrm{h}}$ is the additive value with covariance ${\mathrm{K}}_{\mathrm{a}}{\sigma }_{\mathrm{a}}^{2}$, ${\mathrm{K}}_{a}$ being the additive kinship matrix and ${\sigma }_{a}^{2}$ the additive variance, ${D}_{\mathrm{h}}$ is the dominance value with covariance ${\mathrm{K}}_{\mathrm{d}}{\sigma }_{\mathrm{d}}^{2}$, ${\mathrm{K}}_{\mathrm{d}}$ being the dominance kinship matrix and ${\sigma }_{d}^{2}$ the dominance variance, ${I}_{\mathrm{h}}^{\mathrm{aa}}$, ${I}_{\mathrm{h}}^{\mathrm{ad}}$ and ${I}_{\mathrm{h}}^{\mathrm{dd}}$ are, respectively, the additive-by-additive, additive-by-dominance and dominance-by-dominance epistatic effects with covariance matrices ${\mathrm{K}}_{\mathrm{aa}}{\upsigma }_{\mathrm{aa}}^{2}$, ${\mathrm{K}}_{\mathrm{ad}}{\upsigma }_{\mathrm{ad}}^{2}$ and ${\mathrm{K}}_{\mathrm{dd}}{\upsigma }_{\mathrm{dd}}^{2}$ and variances ${\upsigma }_{\mathrm{aa}}^{2}$, ${\upsigma }_{\mathrm{ad}}^{2}$ and ${\upsigma }_{\mathrm{dd}}^{2}$ and for each of these effects, respectively, and ${E}_{hr}$ is the error vector with each element assumed to be independent with ${\sigma }_{\varepsilon }^{2}$ the error variance.

Specific interaction terms for the additive and dominance effects were added to the model to extend it to a multi-environment context (multiple environments model or MULTI model). We defined environment-specific variances for the error.

$$\begin{gathered} Y_{{{\text{her}}}} = \mu + \theta_{{\text{e}}} + A_{{\text{h}}} + D_{h} + I_{h}^{{{\text{aa}}}} + I_{h}^{{{\text{ad}}}} + I_{h}^{{{\text{dd}}}} + AE_{{{\text{he}}}} + DE_{he} + E_{{{\text{her}}}} \hfill \\ A\sim N\left( {0, K_{a} \sigma_{a}^{2} } \right) , \; D\sim N\left( {0, K_{d} \sigma_{d}^{2} } \right) , \hfill \\ I^{aa} \sim N\left( {0, K_{aa} \sigma_{aa}^{2} } \right),\; I^{ad} \sim N\left( {0, K_{ad} \sigma_{ad}^{2} } \right) , I^{dd} \sim N\left( {0, K_{dd} \sigma_{dd}^{2} } \right) \hfill \\ AE_{e} \sim N\left( {0, K_{a} \sigma_{a\left( e \right)}^{2} } \right) IND,DE_{e} \sim N\left( {0, K_{d} \sigma_{d\left( e \right)}^{2} } \right) IND \hfill \\ E_{e} \sim N\left( {0, I\sigma_{\varepsilon \left( e \right)}^{2} } \right) IND,\quad e \in \left\{ {1; \ldots ;n_{env} } \right\} \hfill \\ A \; \bot D \; \bot I^{aa} \; \bot I^{ad} \; \bot I^{dd} \bot \; AE_{e} \; \bot DE_{e} \; \bot E_{e} \hfill \\ \end{gathered}$$

where ${Y}_{her}$ is the phenotype of the replicate r of hybrid h in environment e, $\mu$, ${A}_{h}$, ${D}_{h}$, ${I}_{h}^{aa}$, ${I}_{h}^{ad}$, ${I}_{h}^{dd}$ are equivalent to effects in MONO model,${\theta }_{e}$ is the environmental effect of environment e, ${AE}_{e}$, ${DE}_{e}$ and ${E}_{e}$ are environment-specific additivity, dominance and error effects, respectively, with environment-specific variances ${\sigma }_{a(e)}^{2}$, ${\sigma }_{d(e)}^{2}$ and ${\sigma }_{\varepsilon (e)}^{2}$ within each environment. Interactions between epistatic effects and environments have been neglected due to weak epistatic effects. ${n}_{env}$ is the number of environments. Here, $IND$ stands for the independence of random effects between environments.

Kinship matrices were computed following Vitezica et al. 2017 for additive and non-additive effects. We defined ${H}_{a}$ and ${H}_{d}$ as the additive and dominant genotyping matrices containing the ${h}_{aim}$(or ${h}_{dim}$) coefficient for individual *i* at the marker *m* coded as follows:

$$h_{{{\text{aim}}}} :\left\{ {\begin{array}{*{20}c} { - \left( {0 - p_{{{\text{Bb}}}} - 2p_{{{\text{bb}}}} } \right)} \\ { - \left( {1 - p_{{{\text{Bb}}}} - 2p_{{{\text{bb}}}} } \right)} \\ { - \left( {2 - p_{{{\text{Bb}}}} - 2p_{{{\text{bb}}}} } \right)} \\ \end{array} } \right.\quad {\text{for}}\quad \begin{array}{*{20}c} {{\text{BB}}} \\ {{\text{Bb}}} \\ {{\text{bb}}} \\ \end{array}$$

$$h_{{{\text{dim}}}} :\left\{ {\begin{array}{*{20}c} { - \frac{{2p_{{{\text{Bb}}}} p_{{{\text{bb}}}} }}{{p_{{{\text{BB}}}} + p_{{{\text{bb}}}} - \left( {p_{{{\text{BB}}}} - p_{{{\text{bb}}}} } \right)^{2} }}} \\ {\frac{{4p_{{{\text{BB}}}} p_{{{\text{bb}}}} }}{{p_{{{\text{BB}}}} + p_{{{\text{bb}}}} - \left( {p_{{{\text{BB}}}} - p_{{{\text{bb}}}} } \right)^{2} }}} \\ { - \frac{{2p_{{{\text{BB}}}} p_{{{\text{Bb}}}} }}{{p_{{{\text{BB}}}} + p_{{{\text{bb}}}} - \left( {p_{{{\text{BB}}}} - p_{{{\text{bb}}}} } \right)^{2} }}} \\ \end{array} } \right.\quad {\text{for}}\quad \begin{array}{*{20}c} {{\text{BB}}} \\ {{\text{Bb}}} \\ {{\text{bb}}} \\ \end{array}$$

with ${p}_{bb}$, ${p}_{bB}$ and ${p}_{BB}$ the genotypic frequencies of bb, Bb and BB, respectively, with b and B the two alleles of locus B.

Kinship matrices for additivity (${K}_{\mathrm{a}}$) and dominance (${K}_{\mathrm{d}}$) were calculated as:

$$K_{{\text{a}}} = \frac{{H_{{\text{a}}} H_{{\text{a}}}^{\prime } }}{{\left\{ {tr\left[ {H_{{\text{a}}} H_{{\text{a}}}^{\prime } } \right]} \right\}/n}}\quad K_{d} = \frac{{H_{{\text{d}}} H_{{\text{d}}}^{\prime } }}{{\left\{ {tr\left[ {H_{{\text{d}}} H_{{\text{d}}}^{\prime } } \right]} \right\}/n}}$$

where $tr(A)$ is the trace of matrix $A$ and n is the number of genotypes. From these two matrices, three epistasis kinship matrices were derived: ${K}_{\mathrm{aa}}$, ${K}_{\mathrm{ad}}$ and ${K}_{\mathrm{dd}}$ for, respectively, additive-by-additive, additive-by-dominance and dominance-by-dominance epistasis.

$$K_{aa} = \frac{{K_{{\text{a}}} \odot K_{{\text{a}}} }}{{\left\{ {tr\left[ {K_{{\text{a}}} \odot K_{{\text{a}}} } \right]} \right\}/n}}\quad K_{ad} = \frac{{K_{{\text{a}}} \odot K_{{\text{d}}} }}{{\left\{ {tr\left[ {K_{{\text{a}}} \odot K_{{\text{d}}} } \right]} \right\}/n}}\quad K_{{{\text{dd}}}} = \frac{{K_{{\text{d}}} \odot K_{{\text{d}}} }}{{\left\{ {tr\left[ {K_{{\text{d}}} \odot K_{{\text{d}}} } \right]} \right\}/n}}$$

where $\odot$ denotes Hadamard product.

### GWAS models

A classical parametrization for the alleles is 1 or 0 according to the presence or the absence of the alleles of the reference genotype (here, the B73 line). In addition, we identified the Dent or Flint origin (D or F, respectively) of the local genetic background surrounding the alleles. Both information was combined into 4 SNP-Origin (SO) alleles: 1D, 0D, 1F and 0F. For example, 1D corresponds to the allele 1 observed in the Dent local genetic background (see Rio et al. 2020). Genotypes were constructed from these alleles following each panel's specificities. Het1 hybrids are DF (for Dent/Flint) at all loci, whereas Het2 hybrids were admixed between both heterotic groups and therefore displayed the three possible genetic backgrounds: DD, DF and FF. As a consequence, 4 SO genotype combinations (SNP-origin genotypes) were observable in Het1 (0D0F, 0D1F, 1D0F and 1D1F), whereas 10 SO genotypes were observable in Het2 (the same 4 genotypes from Het1 and 0D0D, 1D0D, 1D1D, 0F0F, 1F0F and 1F1F in addition).

Two Genome-Wide Association Study (GWAS) strategies were applied to every locus using SO genotypes as marker effects: a single-environment GWAS (Gso-MONO) and a multiple environments GWAS (Gso-MULTI).

For a marker *m*:

$$Y_{{{\text{hcr}}}} = \mu + \beta _{{\text{c}}} + A_{{\text{h}}} + D_{{\text{h}}} + I_{{\text{h}}}^{{{\text{aa}}}} + I_{{\text{h}}}^{{{\text{ad}}}} + I_{{\text{h}}}^{{{\text{dd}}}} + E_{{{\text{hcr}}}} \quad \left( {{\text{Gso-MONO}}} \right)$$

$$Y_{{{\text{hcer}}}} = \mu + \theta_{{\text{e}}} + \beta_{{\text{c}}} + A_{{\text{h}}} + D_{{\text{h}}} + I_{{\text{h}}}^{{{\text{aa}}}} + I_{{\text{h}}}^{{{\text{ad}}}} + I_{{\text{h}}}^{{{\text{dd}}}} + {\text{AE}}_{{{\text{he}}}} + {\text{DE}}_{{{\text{he}}}} + E_{{{\text{hcer}}}} \quad \left( {{\text{Gso-MULTI}}} \right)$$

where ${Y}_{hecr}$ is the phenotype of the hybrid *h*, with the SO genotypes *c* for the marker *m* and the replicate *r* in the environment *e*, ${Y}_{hcr}$ is the same for a specific environment and ${\beta }_{c}$ is the effect of the SO genotypes *c*. The ten SO genotypes effects are redundant with the general mean $\mu$ and thus cannot be uniquely estimated. Other effects are similar to the ones described in MONO and MULTI models. The SO genotypes exploit all available information of markers (4 SO genotypes in Het1 and 10 in Het2). SO genotypes can be connected to the genetic value decomposition developed by Fisher (1918) in the context of biallelic loci.

**Fig. 3:**
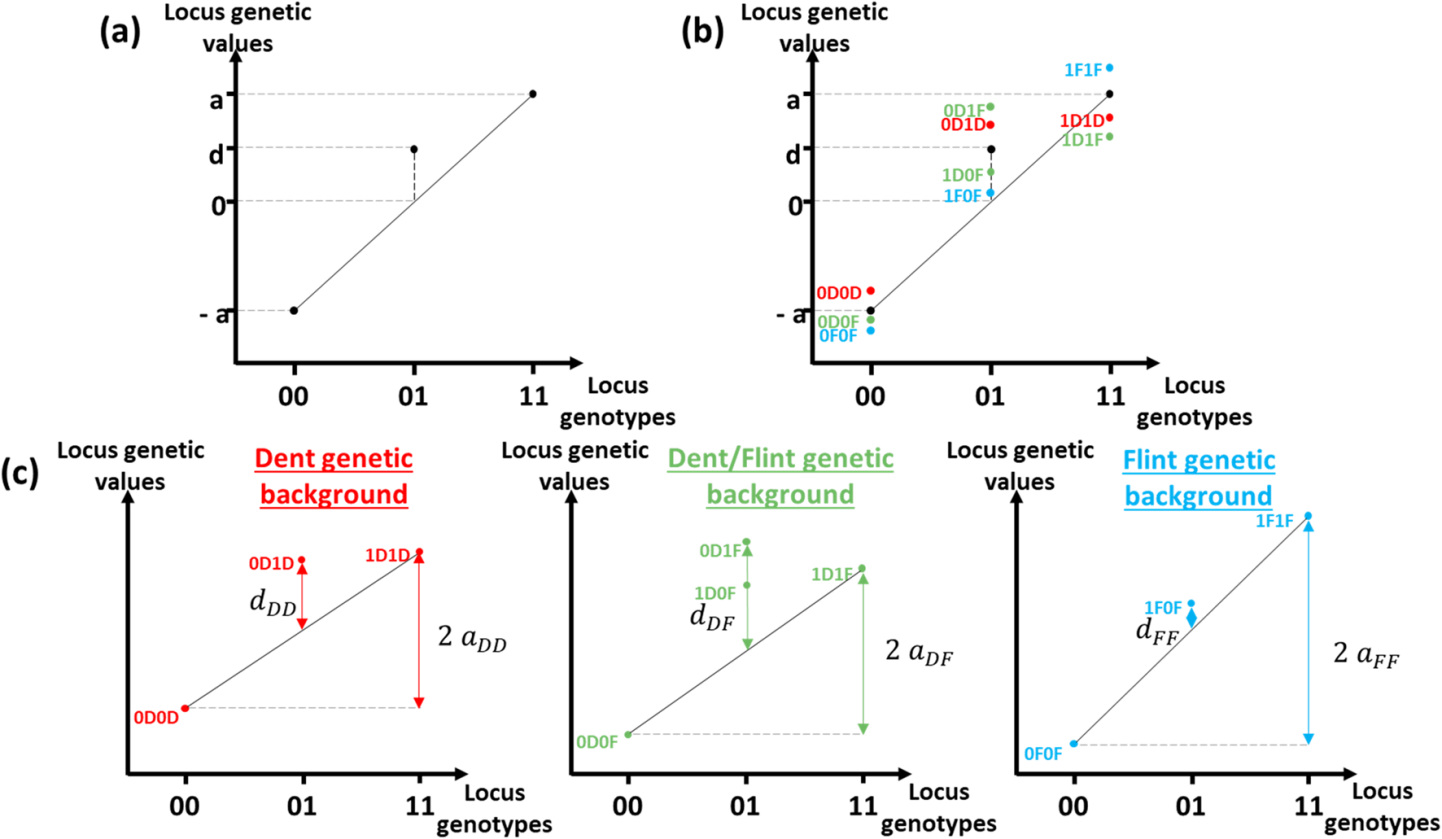
Generalization of the locus biological effects of the Fisher’s model (Falconer et al. (1996) representation) in the case of structured populations. **a** Genetic value description using an SNP-based reference model. **b** Adding genetic background information in the SNP-based model. **c** Decomposition of the SNP-origin model according to the different genetic backgrounds

In this formalism, the genetic value can be decomposed into biological additivity and dominance gene actions (Fisher 1918; Falconer et al. 1996; Lynch and Walsh 1998). Additivity is the effect brought by one allele (for instance: “1”) and is calculated as the half difference between the two homozygous genotype values (difference between 11 and 00). The difference between the mean of homozygous genotypes and the heterozygous genotype value is set as the dominance (Fig. 3a). These biological effects are defined initially for a single unstructured population and did not consider the possibility of different effects according to the genetic structure of the population (Fig. 3b).

In the presence of genetic structure, the biallelic model of Fisher (1918) may be applied within each genetic background, leading to the definition of three additive effects and three dominance effects (Fig. 3c).

$$\left\{ {\begin{array}{*{20}c} {a_{{{\text{DD}}}} = \frac{{\beta_{{1{\text{D}}1{\text{D}}}} - \beta_{{0{\text{D}}0{\text{D}}}} }}{2}} \\ {a_{{{\text{FF}}}} = \frac{{\beta_{{1{\text{F}}1{\text{F}}}} - \beta_{{0{\text{F}}0{\text{F}}}} }}{2}} \\ {a_{{{\text{DF}}}} = \frac{{\beta_{{1{\text{D}}1{\text{F}}}} - \beta_{{0{\text{D}}0{\text{F}}}} }}{2}} \\ \end{array} } \right. \left\{ {\begin{array}{*{20}c} {d_{DD} = \beta_{{1{\text{D}}0{\text{D}}}} - \frac{{\beta_{{1{\text{D}}1{\text{D}}}} + \beta_{{0{\text{D}}0{\text{D}}}} }}{2}} \\ {d_{{{\text{FF}}}} = \beta_{{1{\text{F}}0{\text{F}}}} - \frac{{\beta_{{1{\text{F}}1{\text{F}}}} + \beta_{{0{\text{F}}0{\text{F}}}} }}{2}} \\ {d_{{{\text{DF}}}} = \frac{{\beta_{{0{\text{D}}1{\text{F}}}} + \beta_{{1{\text{D}}0{\text{F}}}} }}{2} - \frac{{\beta_{{1{\text{D}}1{\text{F}}}} + \beta_{{0{\text{D}}0{\text{F}}}} }}{2}} \\ \end{array} } \right.$$

From these definitions, we defined the mean additivity and dominance effects over the different genetic backgrounds as:

$$a = \frac{{a_{{{\text{DD}}}} + a_{{{\text{FF}}}} + a_{{{\text{DF}}}} }}{3}\;\;\; d = \frac{{d_{{{\text{DD}}}} + d_{{{\text{FF}}}} + d_{{{\text{DF}}}} }}{3}$$

Differences between within genetic background additivity or dominance effects reveal particular associations between SNP alleles and allelic origins. Two effects $s$ and $t,$ for both additivity and dominance, were defined: the $s$ effect was defined as the difference between genetic effects measured in the two homozygous backgrounds “DD” and “FF” and the $t$ effect corresponded to the difference between genetic effects measured in the heterozygous background “DF” and the mean of those measured in homozygous backgrounds.

$$\left\{ {\begin{array}{*{20}c} {s_{{\text{a}}} = a_{{{\text{DD}}}} - a_{{{\text{FF}}}} } \\ {s_{{\text{d}}} = d_{{{\text{DD}}}} - d_{{{\text{FF}}}} } \\ \end{array} } \right.\quad \left\{ {\begin{array}{*{20}c} {t_{{\text{a}}} = a_{{{\text{DF}}}} - \frac{{\left( {a_{{{\text{DD}}}} + a_{{{\text{FF}}}} } \right)}}{2}} \\ {t_{{\text{d}}} = d_{{{\text{DF}}}} - \frac{{\left( {d_{{{\text{DD}}}} + d_{{{\text{FF}}}} } \right)}}{2}} \\ \end{array} } \right.$$

Similar to additivity and dominance, two “origin” effects were defined as an effect of the number of Dent origin alleles, ${o}_{a}$, and a specific effect of two alleles with different allele origin ${o}_{d}$. These effects were defined with homozygous SNP genotypes only (00 and 11) as:

$$\left\{ {\begin{array}{*{20}c} {o_{{{\text{a}} 00}} = \frac{{\beta_{{0{\text{D}}0{\text{D}}}} - \beta_{{0{\text{F}}0{\text{F}}}} }}{2}} \\ {o_{{{\text{a}} 11}} = \frac{{\beta_{{1{\text{D}}1{\text{D}}}} - \beta_{{1{\text{F}}1{\text{F}}}} }}{2}} \\ \end{array} } \right. \left\{ {\begin{array}{*{20}c} {o_{{{\text{d}} 00}} = \beta_{{0{\text{D}}0F}} - \frac{{\beta_{{0{\text{F}}0{\text{F}}}} + \beta_{{0{\text{D}}0{\text{D}}}} }}{2}} \\ {o_{{{\text{d}} 11}} = \beta_{{1{\text{D}}1{\text{F}}}} - \frac{{\beta_{{1{\text{F}}1{\text{F}}}} + \beta_{{1{\text{D}}1{\text{D}}}} }}{2}} \\ \end{array} } \right.$$

$$o_{{\text{a}}} = \frac{{o_{{{\text{a}} 00}} + o_{{{\text{a}} 11}} }}{2} o_{{\text{d}}} = \frac{{o_{{{\text{d}} 00}} + o_{{{\text{d}} 11}} }}{2}$$

To complete the description of SO genotypes, ${\Delta }_{\mathrm{LD}}$ effect was defined to distinguish the two double heterozygous SO genotypes 0D1F and 1D0F. This effect was an indicator of the difference in group-specific linkage disequilibrium between the tested marker and a causal QTL.

$${\Delta }_{{{\text{LD}}}} = \beta_{{{\text{0D1F}}}} - \beta_{{{\text{1D0F}}}}$$

We used the previous equations to define contrasts for testing the different additivity and dominance effects (Table 1). In addition, contrasts testing the global effect of the marker (which is equivalent to testing if one SO genotype is different from the others) were developed on the SO genotypes present on each panel (Tables S1 and S2). The contrast ${\mathrm{g}}_{Het2}$ carried out on the 10 SO genotypes observed in the Het2 panel, and the contrast ${\mathrm{g}}_{Het1}$ carried out only on the four common SO genotypes between Het1 and Het2 panels. Each effect was tested through contrast tests based on SO genotypes. Note that all the considered contrasts were identifiable. As the effects were based on contrasts between SO genotypes, they corresponded to biological effects and not statistical effects which depends on the genotypic frequencies (see Fisher 1918 and Falconer, 1996 for a graphical representation of the two types of effects in a biallelic case). As a consequence, it is possible to directly compare the effects based on the same SO genotypes in both panels (additivity and dominance in an inter-origin genetic background (${a}_{\mathrm{DF}}$, ${d}_{\mathrm{DF}}$), the linkage disequilibrium effect ${\Delta }_{\mathrm{LD}}$ and the global test ${\mathrm{g}}_{Het1}$).

**Table 1:**
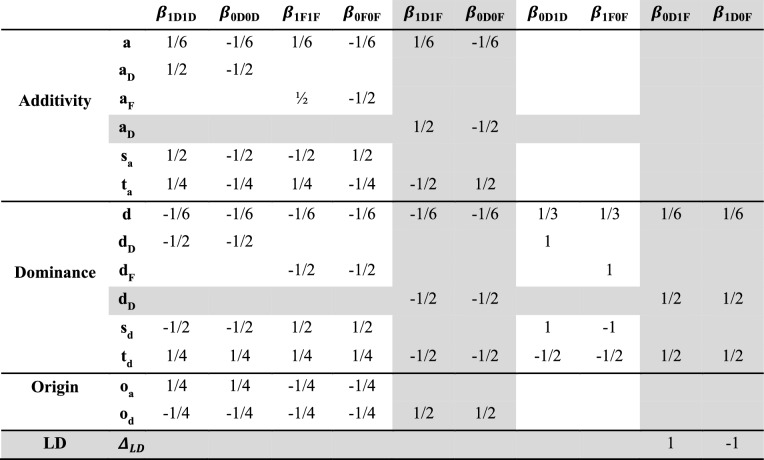
List of contrasts associated with additivity, dominance, origin and linkage disequilibrium

In gray, contrasts tested in both panels. In white, contrasts only tested in the Het2 panel. Only non-null coefficients are displayed.

To limit the redundancy of information between the marker effects and the residual polygenic effects, the GWAS model at marker *m* was fitted by leaving out the chromosome carrying *m* from the kinship matrices calculation (Rincent et al. 2014a, b). In addition, to prevent excessing computation time, the covariances between observations due to polygenic effects were estimated under an H_0_ model without marker effect and were partially re-estimated in the maker-by-marker analysis (See method details of the methodology in Appendix S3). The contrasts were tested using Wald tests. For the two models, Gso-MONO and Gso-MULTI, markers were discarded if their minor SO genotype was carried by less than 5 individuals. Corrections for multiple tests were applied to control the false discovery rate (Benjamini and Hochberg 1995) and were implemented jointly for all environments and separately for each contrast, panel, GWAS model and trait.

### Additive and dominance GWAS model

To illustrate the originality of our method and evaluate its properties, an “additive-dominance” model (further called Gad-MONO and Gad-MULTI, respectively, for single and multi-environment) was used as a benchmark. For a marker *m*:

$$Y_{{{\text{hr}}}} = \mu + x_{{{\text{hr}}}}^{{\text{a}}} \alpha + x_{{{\text{hr}}}}^{{\text{d}}} \delta + A_{{\text{h}}} + D_{{\text{h}}} + I_{{\text{h}}}^{{{\text{aa}}}} + I_{{\text{h}}}^{{{\text{ad}}}} + I_{{\text{h}}}^{{{\text{dd}}}} + E_{{{\text{hr}}}} \quad ({\text{Gad-MONO}})$$

$$Y_{{{\text{her}}}} = \mu + \theta_{{\text{e}}} + x_{{{\text{hr}}}}^{{\text{a}}} \alpha + x_{{{\text{hr}}}}^{{\text{d}}} \delta + A_{{\text{h}}} + D_{{\text{h}}} + I_{{\text{h}}}^{{{\text{aa}}}} + I_{{\text{h}}}^{{{\text{ad}}}} + I_{{\text{h}}}^{{{\text{dd}}}} + {\text{AE}}_{{{\text{he}}}} + {\text{DE}}_{{{\text{he}}}} + {\text{E}}_{{{\text{her}}}} \quad ({\text{Gad-MULTI}})$$

where ${Y}_{\mathrm{her}}$ is the phenotype of the hybrid *h* at the replicate *r* in the environment *e*, ${Y}_{hr}$ is the same for a specific environment, ${x}_{\mathrm{hr}}^{a}$ is an indicator for the statistical additive effect $\alpha$ defined as a regression over the number of SNP allele 1, ${x}_{\mathrm{hr}}^{d}$ is an indicator for the statistical dominance effect $\delta$ set as a deviation from additivity due to the SNP heterozygous state. Other effects are similar to the ones described in MONO and MULTI models. Filters and variance estimation methods were identical to the ones from SO genotype models.

All statistical analyses (variance partition and GWAS) were performed using the MM4LMM R package (Laporte et al. 2022).

### QTL clustering

For a given GWAS, significant markers for a given contrast were grouped into “QTL” based on LD extent (Fig. S1). We adapted a method proposed by Negro et al. (2019) to build LD windows on each side of each significant marker. For a given significant marker and one of the sides of the marker, we proceeded as follows:

1. An LD window of 0.5 cM was initialized. The LD window size was initialized at 0.5 cM
2. The linkage disequilibrium (LD) was calculated (R^2^ computed on SNP hybrid genotypic information) between the significant marker and all the other markers within the LD window. The relation between LD values and genetic distances between markers was then inferred using the Hill and Weir regression model (Hill and Weir 1988). An R^2^ threshold of 0.1 was used to estimate the LD extent.
3. If the LD extent exceeded the LD window length, then the LD window length was increased by 0.1 cM, and the LD extent was estimated again in the new window. This sequence was repeated until the LD extent became inferior to the window length.
4. The final LD extent estimated value was used to define the LD window length.

Significant markers with overlapping LD windows were clustered into the same QTL, and their LD windows were used to define the QTL interval. To compare the different models and panels, we merged the QTLs detected with the MULTI and the different MONO models, *i.e.,* different environments, into a single meta-QTL (referred to in the following sections as QTL) if their intervals overlapped.

## Results

### Variance components and trait heritabilities

After correction by the average values of the checks across the different environments (Table S4), Het1 and Het2 panels displayed on average similar hybrid performances for the flowering times (197.5 days and 196.2 for FloF in Het1 and Het2, respectively) but differed for GY (89.0 and 79.1 q/ha in Het1 and Het2, respectively) and Hum (29.0 and 25.1% in Het1 and Het2, respectively).

Heritabilities were calculated with single and multiple-environment models. Het1 single-environment heritabilities ranged between 0.91 and 0.99 for FloM and between 0.55 and 0.79 for GY (Table S3). The heritabilities evaluated with the multiple-environment model ranged between 0.98 (FloF and FloM) and 0.88 (GY) (Table S4). Het2 showed equivalent results with single-environment heritabilities between 0.79 and 0.97 for FloM and between 0.67 and 0.82 for GY (Table S3). Multiple-environment heritabilities varied between 0.95 for FloM and 0.79 for GY (Table S4). Overall, heritabilities calculated in the multiple-environment model were superior to the single-environment heritabilities.

Large differences between environments were observed for all genetic components of variance in the MONO model (Table S5). The partition of the genetic variance highlighted the preponderance of additivity relative to other effects for all traits and environments. Across environments, the average ratio of additivity variance over the sum of all genetic variances was high in both panels (from 85% (GY) to 91% (FloF) for Het1 and from 73% (GY) to 86% (FloM) for Het2). The average ratio of dominance variance over the sum of genetic variances was inferior to 4% in Het1 for all traits. A higher ratio was observed in Het2 for all traits (up to 17% for GY). The average ratio of epistatic variances across environments was of the same order of magnitude for all traits (from 8% (FloF) to 12% (Hum) in Het1 and from 10% (GY) to 15% (FloF) in Het2).

**Fig. 4:**
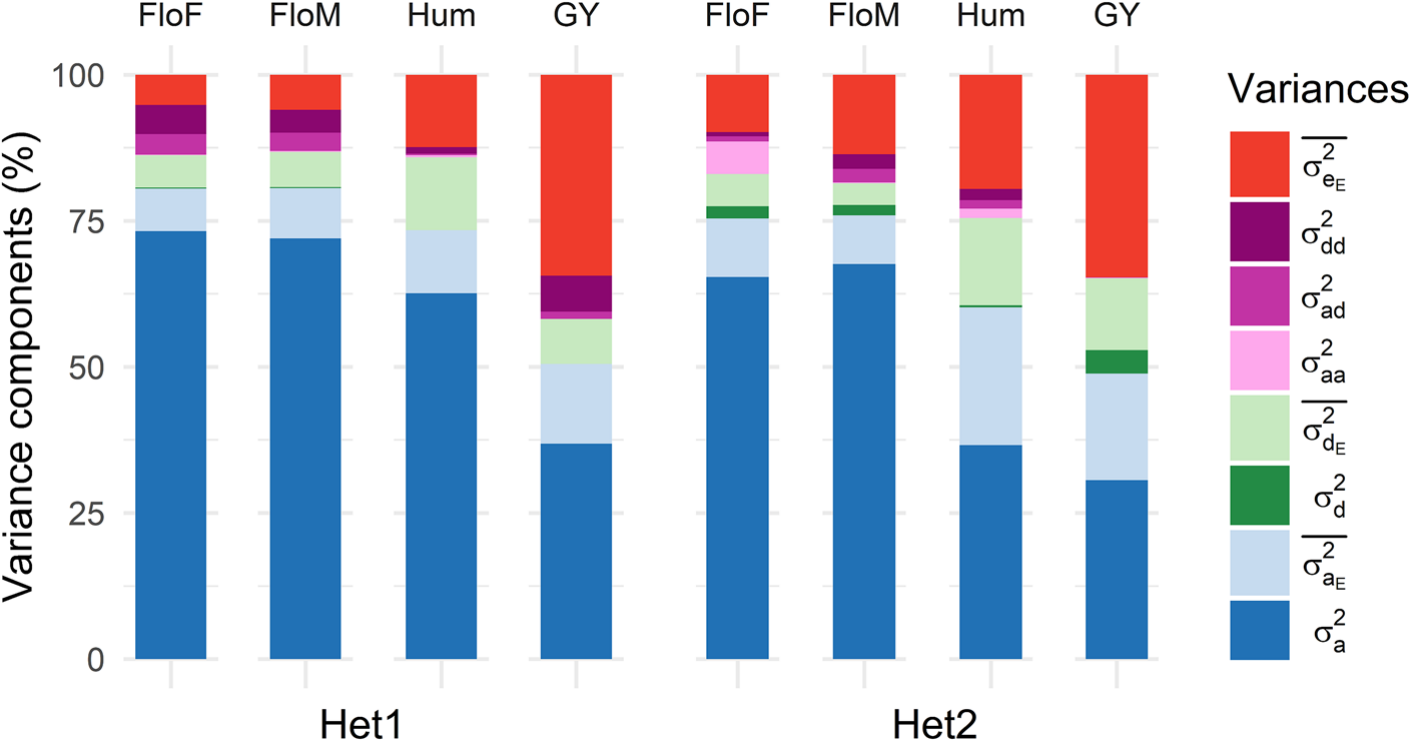
Multiple-environment (MULTI) variance component (in percentage) for Het1 and Het2. The variances ${\sigma }_{\mathrm{a}}^{2}$, ${\sigma }_{\mathrm{d}}^{2}$, ${\sigma }_{\mathrm{aa}}^{2}$, ${\sigma }_{\mathrm{ad}}^{2}$ and ${\sigma }_{\mathrm{dd}}^{2}$ are, respectively, the additive, dominance and additive-by-additive, additive-by-dominance and dominance-by-dominance epistatic variance (see Material & Methods section for details). The average GxE variances $\overline{{\sigma }_{{\mathrm{a}}_{\mathrm{E}}}^{2}}$, $\overline{{\sigma }_{{\mathrm{d}}_{\mathrm{E}}}^{2}}$ and $\overline{{\sigma }_{{\mathrm{e}}_{\mathrm{E}}}^{2}}$ are calculated as the mean over ${\sigma }_{{\mathrm{a}}_{(\mathrm{e})}}^{2}$, ${\sigma }_{{\mathrm{d}}_{(\mathrm{e})}}^{2}$ and ${\sigma }_{{\upvarepsilon }_{(\mathrm{e})}}^{2}$ GxE variances, respectively

MULTI model confirmed the preponderance of additivity in the main genetic variance (defined as the sum of additive, dominance and epistasis variances) (Fig. 4). The percentage of additivity variance (${\sigma }_{a}^{2}$) over the main genetic variance was always superior to 83% (GY) in Het1 and 87% (Hum) in Het2 (Table S8). The percentage of dominance variance (${\sigma }_{\mathrm{d}}^{2}$) over the main genetic variances was higher in Het2 than in Het1 (in particular for GY with 12% for Het2 and < 1% for Het1). The percentage of epistatic variances over the main genetic variance was lower than the MONO model, with values between 3% (Hum) and 17% (GY) in Het1 and between 1% (GY) and 12% (Hum) in Het2.

Genotype-by-environment interaction effects (referred to later on as GxE effects, or AxE and DxE for the additive-by-environment and the dominance-by-environment interaction effects, respectively) were observed for all traits (Tables S6, S7 and S8). The average AxE variance was always inferior to the additive variance for all traits and panels. Interestingly, the average DxE variance was always superior to the dominance variance. This tendency was stronger in Het2 than in the Het1 panel.

### Comparing additive-dominance models and SO genotype models

The GWAS analysis with the SO model consisted of testing for each marker different effects using contrasts between the corresponding SO genotypes (see “GWAS models” section). As the number of SO genotypes differed from one contrast to another, the number of candidate markers ranged from 324,343 to 471,147 markers for Het1 and from 62,955 to 446,478 markers for Het2 (Table S9-12). Two nominal FDR levels were considered (0.05 and 0.2; see Table S13 and S14 for a complete description of the number of significant markers). As expected in GWAS, the distribution of the P values was similar to a uniform law for most of the contrasts (see QQPlots in Fig. S2). Two exceptions were observed for the two contrasts related to the “origin” effects, ${o}_{a}$ and ${o}_{d}$, which showed an inflation of the number of low Pvalues. Manhattan plots revealed that these low Pvalues corresponded to markers located on large chromosomic regions (size up to 156.6 Mbp, Fig. S3).

A standard “additive and dominance” GWAS model (referred to as the Gad model) was used as a benchmark to evaluate the interest of the “SNP-Origin” GWAS model (Gso model). The QTLs were compared within each panel between Gad and Gso models for additivity ($\alpha$ for the Gad model and $a$, ${a}_{DD}$, ${a}_{DF}$, ${a}_{FF}$, ${s}_{a}$ and ${t}_{a}$ for the Gso model) and for dominance ($\delta$ for the Gad model and $d$, ${d}_{\mathrm{DD}}$, ${d}_{\mathrm{DF}}$, ${d}_{\mathrm{FF}}$, ${s}_{\mathrm{d}}$ and ${t}_{\mathrm{d}}$ for the Gso model) (Table 2).

**Table 2:** Comparison of QTLs identified with Gad and Gso models

Panel	Contrast	Trait	Tot^b^	Gad only^c^	Both	Gso only^c^
Het#1	Additivity^a^	FloF	4	1	3	0
		FloM	9	1	8	0
		Hum	21	2	16	3
		GY	4	0	4	0
	Dominance^a^	FloF	1	0	1	0
		FloM	0	0	0	0
		Hum	3	2	1	0
		GY	0	0	0	0
Het#2	Additivity^a^	FloF	49	38	6	5
		FloM	70	60	6	4
		Hum	30	13	9	8
		GY	3	3	0	0
	Dominance^a^	FloF	9	0	0	9
		FloM	14	2	1	11
		Hum	2	0	0	2
		GY	7	0	0	7

QTLs detected with the MULTI and the different MONO models, *i.e.,* different environments, were merged if their intervals overlapped

^a^ “Additivity” and “Dominance” terms correspond, respectively, to the $\alpha$ and $\delta$ effects in the Gad model and $a$, ${a}_{\mathrm{DD}}$, ${a}_{\mathrm{DF}}$, ${a}_{\mathrm{FF}}$, ${s}_{\mathrm{a}}$ and ${t}_{\mathrm{a}}$, and $d$, ${d}_{\mathrm{DD}}$, ${d}_{\mathrm{DF}}$, ${d}_{\mathrm{FF}}$, ${s}_{\mathrm{d}}$ and ${t}_{\mathrm{d}}$ effects in the model Gso

^b^“Tot” indicates the number of all QTLs identified with at least one model

^c^“Gad only” and “Gso only” correspond to specific QTLs only identified with the Gad or Gso model, respectively. The nominal FDR level was set at 0.05

In Het1, most QTLs were commonly identified by the Gad and the Gso models. A total of 7 additive QTLs (and 2 dominance QTLs) were identified with one of the two models only. In Het2, the proportion of QTLs detected by the two models was much lower than in Het1. A large part of the additive QTLs was only detected with the Gad model (43 to 100%), whereas the dominance QTLs were mostly found by the Gso model (79 to 100%).

**Fig. 5:**
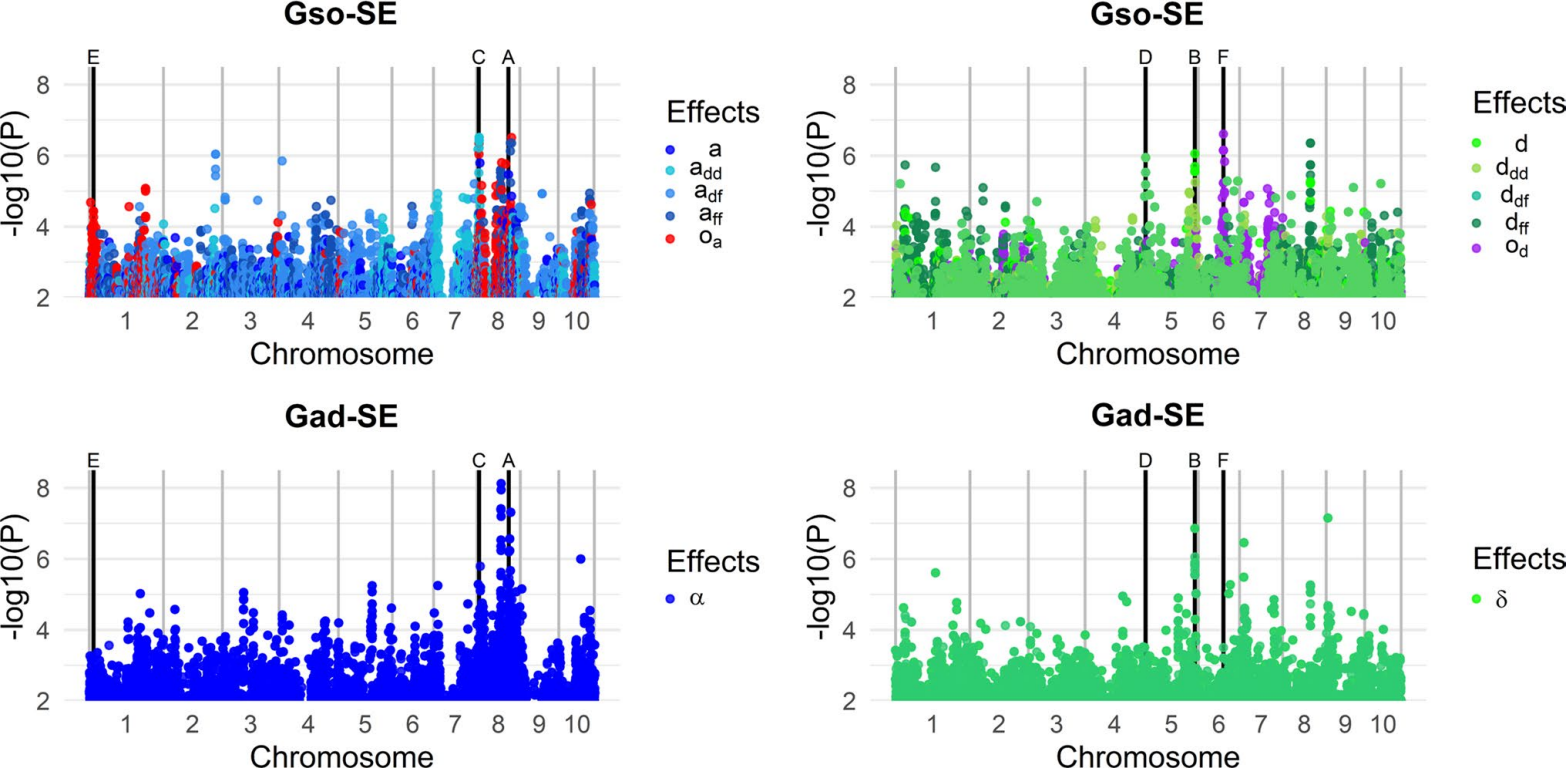
Manhattan plots of additive (blue, left graphs) and dominance (green, right graphs) P values for the Gad-MONO and Gso-MONO models in SOU17 for FloF in Het2. Origin tests were added as elements of comparison in red (on the top left graph) and purple (on the top right graph). Vertical black lines and the letters A to F indicate the position of the representative markers of the Gso model effects, detailed in Fig. 6 (color figure online)

**Fig. 6:**
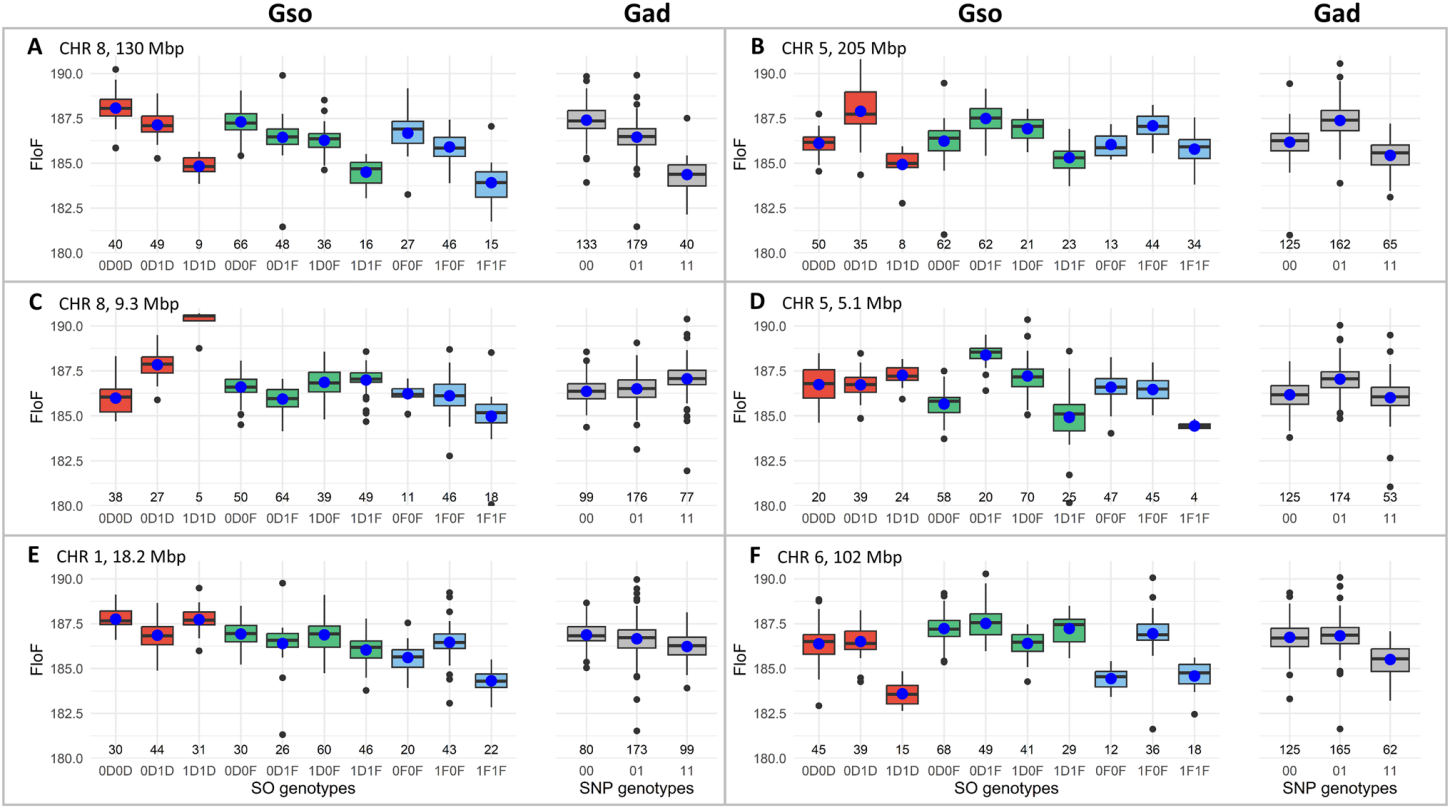
Effects at representative markers detected in SOU17 for FloF in Het2. Boxplot of phenotype corrected by the kinship according to the Gso genotypes for each representative marker (A to F, three left boxplots in red, four middle boxplots in green and three right boxplots in blue are for DD, DF and FF genetic back-grounds for the Gso model, respectively, gray for Gad model). The number at the bottom indicates the number of observations for each SO genotype. Markers A and B represent QTLs identified with both Gad and Gso models, respectively, for additivity and dominance. Markers C and D represent within background effects in the Gso model with no effect in the Gad model. Markers E and F represent the origin effect on the Gso model. Markers A, C and E represent additive effects. Markers B, D and F represent dominance effects (color figure online)

To illustrate the similarities and differences between the Gad and Gso models, we selected representative markers for FloF in the GWAS conducted for environment SOU17 for the Het2 panel (Fig. 5). We represented the SO genotypes for these markers (Fig. 6, see Table S15 for details).

Markers A and B illustrate QTLs detected with both the Gad and Gso models. Marker A showed a strong significant additive $a$ effect (with an effect of − 1.5 days). Within background effects were found for ${a}_{DD}$, ${a}_{DF}$ and ${a}_{\mathrm{FF}}$ with the same magnitude of effects (− 1.6, − 1.4, − 1.4 days, respectively), although less significant. A similar additive effect was identified with the Gad model ($\alpha$ of − 1.5 days). This marker was located in the vicinity of a well-known flowering QTL at 130 Mbp on chromosome 8 (Ducrocq et al. 2008; Bouchet et al. 2017; Rio et al. 2020). Marker B revealed an equivalent situation for the dominance effect. This marker displayed dominance effects with the Gso model for the average dominance effect $d$ (1.7 days) and in all the genetic backgrounds (${d}_{\mathrm{DD}}$ of 2.4 days, ${d}_{\mathrm{DF}}$ of 1.4 days and ${d}_{\mathrm{FF}}$ of 1.2 days).

Markers C and D illustrate the differences between the Gad and Gso models and the consequences of ignoring within background effects in GWAS. Marker C has a strong ${a}_{\mathrm{DD}}$ effect of 2.6 days that was detected in the Gso model but not with the Gad model (P value of 0.22 for the $\alpha$ effect). The specificity of the Dent genetic background is confirmed by the ${s}_{\mathrm{a}}$ test (with an effect of 3.2 and a Pvalue of 9.71e-5). Similarly, marker D showed an effect for dominance within the DF background ${d}_{\mathrm{DF}}$ (with an effect of 2.5 days) but no dominance effect $\delta$ according to the Gad model.

In addition to new QTLs for additivity and dominance, the Gso model allowed us to investigate the origin effect itself. Marker E was spotted for a significant additive effect ${o}_{\mathrm{a}}$ of the origin on chromosome 5 with a Pvalue of 5.36e-5 and an effect of 1.4 days. Marker F illustrated the ${o}_{\mathrm{d}}$ (*P* value of 2.47e-7) with a significant difference between the DF background and the other backgrounds of 2.5 days.

### Trait genetic architecture in hybrid panels as revealed by the Gso model

**Fig. 7:**
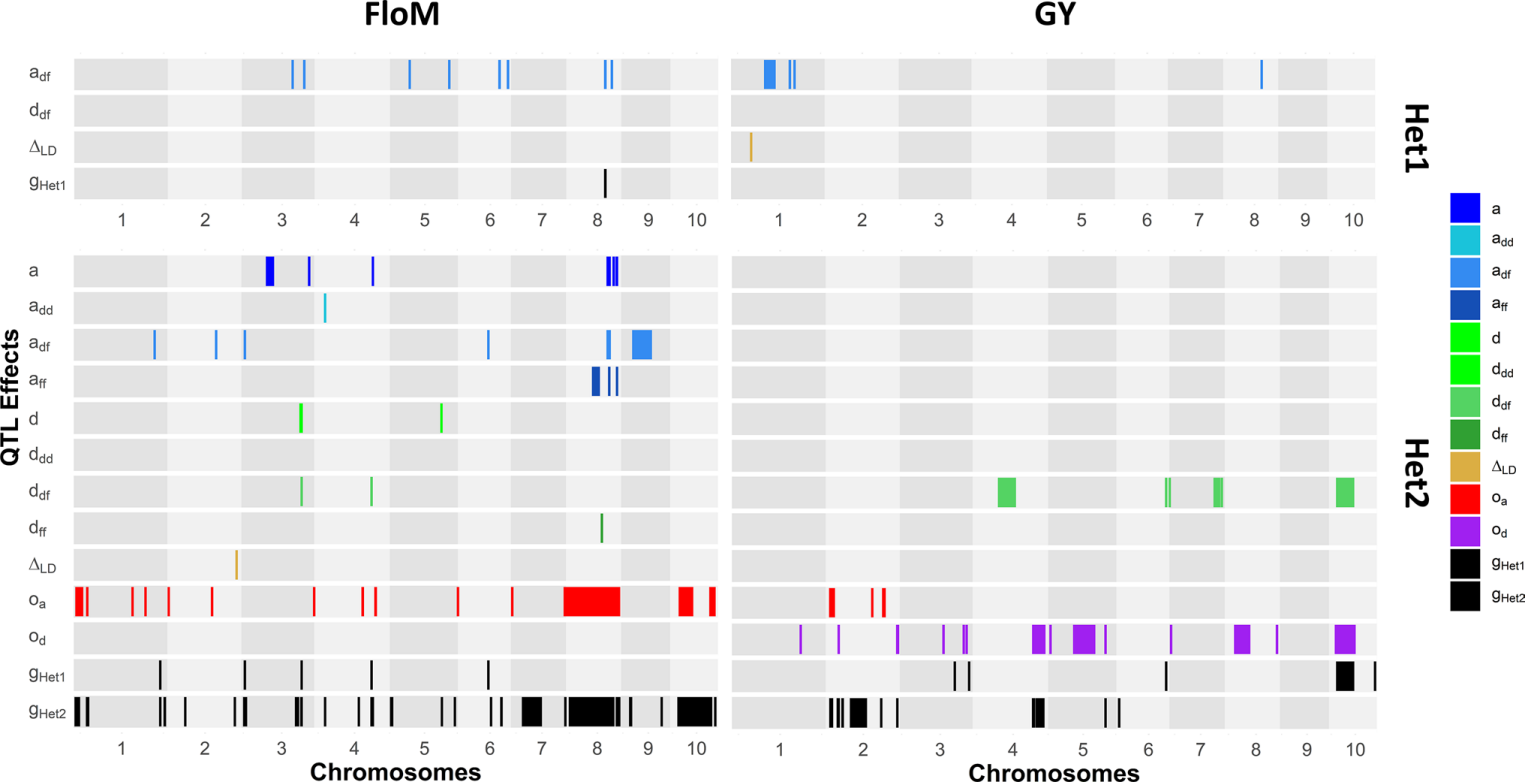
Representation of QTLs for the male flowering time (FloM) and the grain yield (GY) in the Het1 and Het2 panels. Rectangles represent the QTL intervals. All results from the MONO and MULTI models were compiled for the Gso model. The nominal FDR level was fixed at 0.05

Four contrasts were tested in Het1: additivity and dominance in the “Dent-Flint DF” genetic background, ${a}_{\mathrm{DF}}$ and ${d}_{\mathrm{DF}}$, the difference between the two double heterozygous SO genotypes ${\Delta }_{\mathrm{LD}}$ and the global test of the QTL ${g}_{Het1}$ (Table 3, Fig. 7, Fig. S4). Most of the detected QTLs were additive (${a}_{DF}$ effect), especially for Hum (19 QTLs) and FloM (8 QTLs). Dominance ${d}_{\mathrm{DF}}$ QTLs were only found for FloF and GY (1 QTL for each trait). Interestingly, a complementary analysis of the ratio between the additive and the dominance effects revealed that all the significant dominance QTLs were overdominant (|d/a|> 1, see **Table S16**). Only one association for ${\Delta }_{\mathrm{LD}}$ effect was detected for GY. Several QTLs for the global effect ${g}_{Het1}$ were identified (2, 1 and 3 QTLs for FloF, FloM and Hum, respectively).

**Table 3:** Number of QTLs identified for each trait and each contrast

		FloF	FloM	Hum	GY
Het1	${a}_{\mathrm{DF}}$*	3	8	19	4
	${d}_{\mathrm{DF}}$*	1	0	1	0
	${\Delta }_{\mathrm{LD}}$*	0	0	0	1
	${g}_{Het1}$*	2	1	3	0
Het2	$a$	2	6	5	0
	${a}_{\mathrm{DD}}$	4	1	2	0
	${a}_{\mathrm{DF}}$*	7	6	2	0
	${a}_{\mathrm{FF}}$	0	3	10	0
	${s}_{\mathrm{a}}$	0	0	0	0
	${t}_{\mathrm{a}}$	0	0	0	0
	$d$	0	2	0	0
	${d}_{\mathrm{DD}}$	0	0	0	0
	${d}_{\mathrm{DF}}$*	9	2	0	6
	${d}_{\mathrm{FF}}$	0	1	2	0
	${s}_{\mathrm{d}}$	0	0	0	0
	${t}_{\mathrm{d}}$	0	8	0	1
	${\Delta }_{\mathrm{LD}}$*	5	1	1	0
	${o}_{\mathrm{a}}$	0	29	0	8
	${o}_{\mathrm{d}}$	3	0	0	15
	${g}_{Het1}$*	14	5	2	5
	${g}_{Het2}$	33	40	1	13

QTLs detected with the Gso-MULTI and the different Gso-MONO models, *i.e.,* different environments, were merged if their QTL intervals overlapped. Tested effects common to the Het1 and Het2 are marked with an asterisk (1D1F, 1D0F, 0D1F and 0D0F). The nominal FDR level was fixed at 0.05

As in Het1, we identified a large number of additive QTLs (with $a$, ${a}_{\mathrm{DD}}$, ${a}_{DF}$, ${a}_{FF}$, ${s}_{a}$ and ${t}_{a}$ effects) for all the traits (except GY) in Het2 (Table 3, Fig. 7, Fig. S4). FloF and FloM displayed mostly ${a}_{DF}$ QTLs (7 and 6 QTLs, respectively), whereas Hum showed an important number of ${a}_{FF}$ QTLs (10 QTLs). No additive QTLs were detected for GY. QTLs associated with a dominance effect ($d$, ${d}_{DF}$ and ${d}_{FF}$) were identified for all traits, in a higher number than in the Het1 panel. FloM and FloF displayed mostly ${d}_{DF}$ effects (with 9 and 2 QTLs, respectively), whereas Hum was characterized by ${d}_{FF}$ (2 QTLs) effects. Dominance QTLs were identified for GY exclusively for ${d}_{DF}$ (6 QTLs). No QTL was identified for ${s}_{d}$ and only a few for ${t}_{d}$ for FloM and GY. As in Het1, all significant dominance QTLs were identified with an overdominance effect (Table S16). Moreover, the ${d}_{DF}$ QTLs corresponded to the most important differences of SNP allelic frequencies between the Dent and the Flint group (Fig. S5). In addition to additive and dominance QTLs, QTLs associated with an effect of the origin (${o}_{a}$ and ${o}_{d}$) were found for all traits except Hum. FloM displayed essentially additive effect of the origin (29 QTLs) whereas GY showed both additive and dominance effects of the origin (8 and 15 QTLs, respectively). ${\Delta }_{LD}$ QTLs were mostly detected for FloF (5 QTLs). The test for the global effect of the QTL ${g}_{Het2}$ revealed numerous QTLs for all traits in particular for FloF, FloM and GY. The test of the contrast including the four SO genotypes also present in Het1 (${g}_{Het1}$) revealed QTLs for all traits but fewer than with ${g}_{Het2}$.

### QTL co-location between multiple environments

The comparison between QTLs detected with the Gso-MONO model in the different environments provided insight into their stability. In order to compare QTLs detected in different environments, a QTL merging procedure was applied. This was done separately for additive ($$a$$, $${a}_{DD}$$, $${a}_{DF}$$, $${a}_{FF}$$, $${s}_{a}$$ and $${t}_{a})$$, dominance ($$d$$, $${d}_{DD}$$, $${d}_{DF}$$, $${d}_{FF}$$, $${s}_{d}$$ and $${t}_{d}$$) and origin ($${o}_{a}$$ and $${o}_{d}$$) contrasts, respectively (Table S17 and S18).

In the Het1 panel (Table S17), QTLs were mostly found in a single environment. Only 3 QTLs were identified in at least two environments, and all of them were also found with the Gso-MULTI model for the flowering traits (1 and 2 QTLs for FloF and FloM, respectively). One of these QTLs, located on chromosome 8 at position 119 – 124 Mbp, was shared by both flowering traits. No dominance QTL was identified in more than two environments. Gso-MULTI did not reveal any additional QTL compared to those found with the Gso-MONO model.

As in Het1, only a few QTLs co-locating between environments were identified in Het2. Additive QTLs were identified in at least 2 environments for FloF (1 QTL), FloM (3 QTLs) and Hum (2 QTLs) (Table S18). Interestingly, among them, several additive QTLs were detected for both flowering time traits (FloF and FloM) on chromosomes 4 and 8. No dominance QTLs were identified as co-locating between environments. The additive effect of the origin showed several co-locating QTLs for FloF (4 QTLs). No co-locating QTLs were identified for ${\mathrm{o}}_{d}$.

Most of the strong co-locating QTLs identified in Het2 with the Gso-MONO model were confirmed with the Gso-MULTI model. Conversely, Gso-MULTI identified new QTLs undetected with Gso-MONO, e.g., 5 additive QTLs and 7 dominance QTLs for FloF.

### Comparison between factorial and admixed diallel hybrid panels

We compared the four contrasts that were common to Het1 and Het2: ${a}_{DF}$, ${d}_{DF}$,${\Delta }_{LD}$ and ${g}_{Het1}$. We considered a QTL to be detected in both panels if the QTLs intervals overlapped. For the comparable effects between Het1 and Het2, we did not detect any common QTL. Note that when considering a less stringent FDR level (FDR level of 0.2, Table S19), we found several shared additive QTLs for FloF (18 QTLs), FloM (7 QTLs) and Hum (5 QTLs) and shared dominance QTLs for FloF (3 QTLs) and GY (4 QTLs). Some of them matched with regions identified as co-locating between environments in both panels: for FloF on chromosome 4 (22 – 162 Mbp) and CHR 8 (117 – 141 Mbp) and for FloM on chromosome 8 (119–141 Mbp). Nevertheless, the majority of QTLs were specific to one of the panels. On average, over the contrasts, we identified more additive and dominance specific QTLs in Het2 than in Het1 for the flowering traits. On opposite, more QTLs were found in Het1 than in the Het2 panel for Hum. GY displayed a different situation with more additive QTLs identified in Het1 but more dominance QTLs identified in Het2.

## Discussion

### Genetic architecture of agronomic traits

Variance partition and GWAS results provided some insight into the genetic architecture underlying the traits analyzed in this study. High heritabilities were found for all traits. These high values were due to the large genetic diversity present in the Dent and in the Flint inbred line panels used to derive the two hybrid panels. This large initial diversity explains the large genetic variances found (Tables S3-5) that led to the high trait heritabilities. For both panels, the majority of the genetic variance of all traits was due to additive effects (Tables S5-S8). The additive variance was important for the flowering traits, with values comparable to previous reports (Rogers et al. 2021; Roth et al. 2022). It has to be noted that the additive variance estimated in this study corresponds to statistical additivity and includes part of the dominance and epistatic biological effects (Vitezica et al. 2013). Several authors proposed new models accounting for non-additive effects (Su et al. 2012; Vitezica et al. 2013; Da et al. 2014). In these models, variances associated with non-additive effects reflect deviation from the above-defined statistical additivity. In this study, the contribution of non-additive effects differed highly from one trait to another. In Het2, the proportion of dominance over the global genetic variance was higher for grain yield (12% in MULTI models) than for flowering traits (2 to 3%), consistent with previous work where dominance variance explained 4 to 10% of the genetic variance for male flowering time and 14 to 20% for the grain yield (Dias et al. 2018). Other studies showed a prevalence of dominant variance for yield (Zdunic et al. 2008), with up to 81% of the genetic variance due to dominance variance (Ceballos et al. 1998).

The Het1 panel was issued from a factorial mating design between two unrelated heterotic groups, whereas Het2 was issued from a diallel mating design between admixed Dent-Flint lines. By construction, Het2 hybrids showed variable levels of inbreeding (Roth et al. 2022) and were expected to display a larger proportion of dominance variance than Het1. This dominance variance is partially "fixed" within the Het1 panel, whereas the Het2 panel was developed to unleash this fixed variance. Consistently, we observed higher dominance variance components in Het2 than in Het1, especially for grain yield, which shows large heterosis.

### The relative importance of additive and dominance QTLs

Our GWA studies revealed QTLs associated with additive and non-additive effects (Table 3). Flowering traits and grain moisture presented numerous additive QTLs, consistent with previous analyses that showed that most of the loci controlling flowering time were additive (Buckler et al. 2009). Some of the QTLs we detected have been repetitively found in other studies. It is the case of the vgt1-vgt2 region on chromosome 8 for the flowering traits (Chardon et al. 2004; Salvi et al. 2007; Ducrocq et al. 2008; Buckler et al. 2009; Bouchet et al. 2013, 2017; Castelletti et al. 2014; Rincent et al. 2014a, b; Rio et al. 2019; Mayer et al. 2020). We identified these QTLs in both panels and in all environments, highlighting the high stability of their effects across environmental conditions. Beyond this region, it would have been interesting to compare our QTLs with the ones found in previous studies and look for candidate genes. Such comparisons were beyond the scope of this study and were limited by the large size of our QTLs intervals (from few centimorgans to more than 10 centimorgans) which could contain multiple potential candidate genes, especially for integrative traits such as grain yield.

In addition to additive QTLs, several dominance QTLs were also found for the flowering traits in Het2. We hypothesize that inbreeding leads to a delay in reproductive organ growth and, therefore, in the anthesis or silking date. A large number of dominance QTLs was found as well for grain yield, which is known for being highly impacted by inbreeding depression. For all traits, all the detected dominance QTLs showed overdominant effects (Table S16). Several other studies pointed out the importance of the contribution of overdominance QTLs to heterosis (Lippman and Zamir 2007), particularly in the centromeric regions with low recombination rates, leading to hypothesize that mostly pseudo-overdominance is involved (Larièpe et al. 2012). In this study, we suspect a lack of detection power to identify partial dominance QTLs compared to overdominance (or pseudo-overdominance) QTLs due to a lower amplitude of the QTL effects. Also, the number of recombination events between Het1 and Het2 panels was too low to break the tight linkage disequilibrium between dominance QTLs in repulsion, which favored the identification of pseudo-overdominance QTLs. At the hybrid level, dominance QTLs contribute to the hybrid performance by generating heterosis. Moreover, unbalanced allelic frequencies between the Dent and Flint population favored the expression of heterosis, especially in the DF background generating a complementarity between the heterotic groups.

We observed a difference between the results of the variance partition and the number of QTLs identified as having dominance or additive effects. For example, for grain yield, the additive variance was more important than the dominance variance (in mono and multi-environments), whereas the GWAS revealed mostly dominance QTLs for this trait. This discrepancy can be explained by two reasons. First, the effects tested in the variance partition and the GWAS model differed. The variance partition is based on the statistical QTL effects (breeding value $\alpha$ and dominance deviation $\delta$, tested in the Gad model) that depend on both the biological additive and dominance effects ($a$ and $d$) and the allelic frequencies in the population (Fisher 1918; Falconer et al. 1996; Lynch and Walsh 1998), whereas the Gso GWAS model is based on biological additive and dominance effects ($a$ and $d$). Huang and Mackay (2016) reported that variance components do not provide information on the average gene action and alarmed about the false inference of genetic architecture based on variance decomposition, which tends to overestimate the contribution of additivity. Variance partition is nevertheless helpful for estimating the proportion of the phenotypic variance associated with the breeding values. Secondly, the detected QTLs explained only part of the genetic variability. We hypothesize that many QTLs with minor effects were not detected due to statistical limitations, even though they contributed to the overall genetic variation. This phenomenon is known as the "missing heritability problem" (Maher 2008). For example, according to the findings of Xiao et al. (2017), the numerous QTLs for flowering time identified by Buckler et al. (2009) and Tian et al. (2011) only account for a small proportion (less than 7.5%) of the total genetic variance of these traits.

### Stability of QTLs across environments

We investigated GxE interactions with two approaches: an analysis of the variance partition at a multi-trial scale and a study of the stability of the QTLs identified in GWAS across environments. First, we showed in both panels that variance partitions differed from one environment to another, confirming the role of GxE across environments. Interestingly, additivity and dominance did not interact in the same way with the environment. The comparison between the main genetic variance and the GxE variances revealed the important contribution of the additivity to the main genetic variance, whereas the dominance displayed a higher amount of GxE variances (Table S8) as shown by Buckler et al. (2009), Romay et al. (2013) Peiffer et al. (2014) and Rogers et al. (2021). In particular, in Het2, we identified important dominance-by-environment variances for grain yield.

Differences in QTL effects between environments are expected in the presence of GxE interactions. Bernardo (2008) suggested that GxE interaction is one of the principal explanations for the inconsistency between the results of GWAS analysis of complex traits. For all traits and in both panels, we showed that most detected QTLs were specific to one single environment. In particular, almost all the dominant QTLs for grain yield were identified in only one environment. In addition to the presence/absence of detected QTLs between environments, we also observed variation in the amplitude or the sign of the effects (as illustrated in Fig. S6 in DD genetic background). Boer et al. (2007) found similar results, showing that GxE QTLs represent a significant part of the QTLs identified for yield and grain moisture.

### The benefits and drawbacks of hybrid admixed panels

To evaluate the interest of admixed panels in GWAS, we compared two hybrid panels, one obtained from a classical factorial crossing design between heterotic groups and one obtained from a diallel crossing design between admixed lines. These panels were related to each other since they were both issued from the same founder lines. The relatedness between the two panels and the use of the Gso model based on biological effects should have led to the detection of some common QTLs in the two panels. However, all the QTLs detected with an FDR of 0.05 were specific to one of the two panels. This can be explained by a limited detection power in both panels, leading to the detection in each of a limited set of QTLs. Indeed, by relaxing the FDR level at 0.2, we found more QTLs shared by the two panels (Table S19), including an additive QTL corresponding to well-known flowering time QTLs vgt1 and vgt2 on chromosome 8. As Het1 only displayed Dent-Flint genotypes, i.e., four genotype classes, the number of observations in each genotypic class was higher than in Het2, where the total number of observations was split into ten genotypic classes. Therefore, for the contrasts that can be detected in both panels, the detection power was expected to be higher in Het1 than in Het2. In addition to this power issue, the panels were evaluated in different environments and in different years. We showed previously that different environments could lead to different QTL results (Fig S6), whatever the panel and this effect might be even stronger when comparing two different panels. Lastly, an essential difference relied on the ability to test unique effects with the Gso model in Het2. The admixture within the hybrid genotypes allowed us to test the effects according to the genetic background and of the origin effect itself.

Beyond their relevance for GWAS, admixed inbred lines raise issues in terms of hybrid breeding since they deviate from the heterotic patterns used by breeders. Het2 hybrid indeed had on average lower performances than Het1 hybrids for grain yield, which can be explained by the potential inbreeding depression associated with Dent/Dent or Flint/Flint segments. Creating lines from commercial hybrids, which typically leads to admixture, is nevertheless a frequent practice in developing countries to complement these created from open pollinated varieties (Guo et al. 2013). Admixed inbred lines developed from commercial hybrids can provide an interesting source to enrich diversity and sustain genetic gain. In this context, using our GWAS approach in a hybrid admixed panel might be helpful to distinguish haplotype blocks to introduce in a given breeding pool from those that should be discarded as they may contribute inbreeding depression in hybrids with the complementary pool.

### The benefits and limits of the Gso model for QTL detection

We compared the Gso GWAS and a more traditional Gad GWAS, not accounting for the allelic ancestry (Table 2, Figs. 5 and 6). We found several QTLs shared by the two models, in particular in Het1. Nevertheless, most QTLs were detected with only one of the two models. The first difference between the two models was the way effects were defined. In the Gso model, they were defined as a linear combination of the estimated value of the Gso genotypic classes. This approach allowed testing any possible effect after orthogonalization for other effects. Nevertheless, a drawback was the need to estimate all Gso genotypic class parameters. The number of observations was split between all Gso genotypic classes, and some were more frequent than others by construction. In Het2, the DF background had a higher frequency (50% of the individuals at a given locus on average) than FF and DD backgrounds (25% each). The SNP allelic frequencies also affect the probability of observation of each genotypic class. The limited representation of genotypic classes reduced the precision of estimation and the detection power for effects to the point where it was impossible to test certain effects involving such classes. In the Gad model, the effects were tested as a regression over the number of SNP alleles for the additivity and a deviation due to the heterozygous SNP genotype for the dominance. $\alpha$ and $a$ tests (or $\delta$ and $d$) were not completely equivalent (correlation of 0.92 was estimated between the Gad and Gso effect estimates for additivity on average across traits and environment, 0.90 for dominance, see **Table S20**). Therefore, the number of estimated parameters differed between Gad and Gso models, with 2 parameters for Gad and 4 to 10 (respectively, for Het1 and Het2) in Gso. The exploitation of all the degrees of freedom available in the Gso model allows testing effects (within genetic background, comparison test, origin…) unavailable in the Gad model (see part "Gso effects: an indicator of the heterotic group complementarity and differentiation").

QTLs only with the Gad model can be due to the lower power of the Gso model in case of lower representation of genotypic class in the Gso model compared to the Gad model or QTLs, which are strongly differentiated between the heterotic groups. In this last case, the QTLs cannot be tested with the Gso model due to the filter on the genotypic classes. By testing effects overall the genetic backgrounds, the Gad model is more parsimonious in terms of estimated parameters allowing a gain of detection power compared to effects nested within genetic backgrounds (Rebai and Goffinet 1993; Jannink and Jansen 2001; Bardol et al. 2013; Giraud et al. 2014). However, ignoring the levels of complexity brought by the genetic background may have a cost in detection power when allelic effects vary (Jannink and Jansen 2001; Rio et al. 2020). Several authors suggested to use of models integrating local genetic background information (Tang et al. 2010; Pasaniuc et al. 2013; Zhang and Stram 2014; Aschard et al. 2015; Skotte et al. 2019), and a model using SO alleles was proposed by Rio et al. (2020) on admixed DH lines. In this study, we proposed an adaptation of Rio et al. (2020) method to identify background QTLs in the context of hybrid populations.

### Gso effects: an indicator of the heterotic group complementarity and differentiation

In this work, we presented a new GWAS strategy for evaluating the variations of QTL effects according to allele group origin. To our knowledge, there is no other already available GWAS model allowing one to account for group origin effects and dominance effects. Some software, such as rrBLUP (Endelman 2011) account for additive kinship and non-additive Gaussian kernels but only test additive effects of the markers in GWAS. The software TASSEL (Bradbury et al. 2007) tests additive and dominance marker effects and any kinship matrices can be specified. It also proposes to use contrast tests for the marker effects, but only relies on SNP genotypes. Other methods, like the one implemented in R/qtl2 (Broman et al. 2019), includes the possibility of estimating founder effects but does not make it possible to model group origin effects and dominance effects. These methods were not suitable for our specific application case as they do not account for the population origin of the alleles and admixed hybrid panels.

We proposed to use the Gso model to identify several effects: within background, additive or dominance effect of the origin. A within background QTL indicates a particular interaction between the SNP effects and the local genetic background surrounding the marker due to: (i) a different linkage disequilibrium (LD) between SNPs and QTLs across groups, (ii) within group genetic mutations in QTL regions and/or (iii) local epistatic interactions between QTLs and other loci that have differentiated allele frequencies between groups.

Previous articles reported within genetic background QTLs (Charcosset et al. 1994; Rebaï et al. 1997; Blanc et al. 2006; Giraud et al. 2014; Rio et al. 2020). We reported a dominance QTL in the DF background ($${d}_{DF})$$ for the grain yield on chromosome 10 (20–70 Mbp) with a P value of 10e-8 that coincides with previous findings for genetic background interaction (Blanc et al. 2006). Dominance QTLs in the DF background with unbalanced frequencies (Fig. S5) between the heterotic groups contribute to the complementarity between the heterotic groups. We revealed another important region for a within background additive effect ($${a}_{DD}$$ and $${a}_{DF}$$) on the chromosome 8. Previous studies (Buckler et al. 2009; Rio et al. 2020) pointed out that this region surrounding two major QTLs for flowering time (*vgt1* and *vgt2*) shows unbalanced allelic frequencies between heterotic groups, underlying the differentiation of the heterotic groups.

Different patterns of within background QTLs appeared between the traits (Table 3). Significant additive effects were found in all three genetic backgrounds ($${a}_{DD}$$, $${a}_{DF}$$ and $${a}_{FF}$$) for the flowering traits, but mainly in the DF background for FloF. Grain moisture presented mostly QTLs in the FF backgrounds ($${a}_{FF}$$ and $${d}_{FF}$$). Grain yield showed dominance QTLs only in the DF background ($${d}_{DF})$$. One explanation to these differences in behavior may correspond to variations of expression of intra- or inter-group epistatic effects (González-Diéguez et al. 2021).

We highlighted only a few QTLs for the comparisons between within background effects (${t}_{a}$, ${s}_{a}$, ${t}_{d}$ and ${s}_{d}$), in particular between the additivity in the DF background and the DD and FF backgrounds for the grain yield. These QTLs marked the differentiation of effect between heterotic groups. The low number of QTLs associated with these tests despite the number of within background QTLs can be explained by the lack of detection power in the Het2 panel. The test ${\Delta }_{LD}$ comparing the two double SNP and origin heterozygous genotypes (1D0F and 0D1F) was mostly found significant for the female flowering time in Het2, but the majority of the QTLs identified were unstable across panels and environments.

In addition to within background QTLs, we identified numerous QTLs with an effect of the origin for all traits except grain moisture (Table 3). The length of these QTL intervals varied greatly, from a few centimorgans up to the size of a chromosome (${o}_{a}$ effect on chromosome 8 for the male flowering time). The large size of these QTLs can be explained by the limited resolution of the origin information in Het2. For male flowering, the genetic structure in heterotic groups is not completely reshuffled in Het2 due to the low number of recombination events on each chromosome between Het1 and Het2. Some large chromosomic segments shared the allelic origin information. Whenever a segment is associated with the phenotypic variation, most markers within the segment stood out as significant, leading to a large number of positive results.

Major regions for the additive effect of the origin (${o}_{\mathrm{a}}$) were identified for the male flowering time, in particular on the chromosome 8 (with effect values up to 2.2 days) in the vicinity of the known region for SNP effects (13–170 Mbp). Other regions were found on chromosomes 1, 2, 7 and 10. Grain yield displayed mostly dominance effect of the origin (${o}_{\mathrm{d}}$) with a strong QTL on chromosome 4 (187–231 Mbp) of 11.4 qtx/ha. Origin dominance effects contribute to the superiority of the inter-group hybrids, *i.e.,* the complementarity between heterotic groups. Suppose a locus with completely differentiated allelic frequencies between the groups and a dominance effect. It cannot be tested through the Gso dominance effects in Het2 due to filtering on the genotypic classes (see above). But testing the origin effect of another locus with balanced frequencies in the vicinity is equivalent to testing the hidden SNP dominance effect of the causal locus. An origin dominance effect, therefore, indicates the proximity of highly differentiated QTLs with dominant effects that are involved in the complementarity between heterotic groups.

Compared to other traits, grain moisture did not reveal any effect of the origin. As the Dent group is known for having delayed silking and anthesis dates (Rebourg et al. 2003; Dubreuil et al. 2006; Unterseer et al. 2016) and quicker desiccation (Hunter et al. 1979) compared to the Flint group, we hypothesize that both phenomena compensate each other, resulting in no group difference in allele effect for the grain moisture measured at harvest and therefore no correlation between the origin and the phenotype.

## Conclusions

This study introduced a new GWAS method to describe the genetic architecture underlying hybrid performance for agronomic traits, the differentiation and the complementarity between heterotic groups. We identified the main additive QTLs for the flowering traits and emphasized the importance of dominance in the hybrid performance, in particular for grain yield. We showed that most of all dominance QTLs exhibit overdominance, or more likely, pseudo-overdominance QTLs. Such genomic regions justify maintaining locally differentiated heterotic groups to fix the favorable form in the hybrid population but are responsible for the limitations of the transfer of these regions from one group to another. Dominance QTLs, in addition to unbalanced frequencies between heterotic groups, are one of the major factors of heterotic group complementarity. Thanks to the Gso model disentangling the effect of the allele from the effect of the origin and the use of admixed lines between the heterotic groups, we highlighted new QTLs, usually hidden in the regular factorial hybrid panel such as Het1. We identified within background QTLs in the Dent, Flint and Dent-Flint backgrounds for the additive and the dominance effect. These QTLs are the result of the differentiation between the heterotic groups. In addition, we found as well several regions with an effect of the allelic origin itself in Het2. In particular, the dominance effect of the origin can detect the presence of closed dominance loci with unbalanced frequencies involved in the complementarity between heterotic groups.

### Supplementary Information

Below is the link to the electronic supplementary material.

Supplementary file1 (DOCX 1667 KB)

Supplementary file 2 (DOCX 20 KB)

## Data Availability

Data are available from the Data INRAE Institutional Data Access (contact via https://data.inrae.fr/dataset.xhtml?persistentId=doi:10.57745/OSNEQM&version=DRAFT for Het1 panel, https://data.inrae.fr/dataset.xhtml?persistentId=doi:10.15454/ZGP766 for Het2 panel) for researchers who meet the criteria for access to confidential data.

## References

[CR1] Álvarez-Castro JM, Carlborg Ö (2007). A unified model for functional and statistical epistasis and its application in quantitative trait loci analysis. Genetics.

[CR2] Aschard H, Gusev A, Brown R, Pasaniuc B (2015). Leveraging local ancestry to detect gene-gene interactions in genome-wide data. BMC Genet.

[CR3] Bardol N, Ventelon M, Mangin B (2013). Combined linkage and linkage disequilibrium QTL mapping in multiple families of maize (*Zea mays* L.) line crosses highlights complementarities between models based on parental haplotype and single locus polymorphism. Theor Appl Genet.

[CR4] Benjamini Y, Hochberg Y (1995). Controlling the false discovery rate: a practical and powerful approach to multiple testing. J R Stat Soc Ser B Methodol.

[CR5] Birchler JA, Yao H, Chudalayandi S (2010). Heterosis. Plant Cell.

[CR6] Blanc G, Charcosset A, Mangin B (2006). Connected populations for detecting quantitative trait loci and testing for epistasis: an application in maize. Theor Appl Genet.

[CR7] Boer MP, Wright D, Feng L (2007). A mixed-model quantitative trait loci (QTL) analysis for multiple-environment trial data using environmental covariables for QTL-by-environment interactions, with an example in maize. Genetics.

[CR8] Bordes J, de Vaulx RD, Lapierre A, Pollacsek M (1997). Haplodiploidization of maize (*Zea mays* L.) through induced gynogenesis assisted by glossy markers and its use in breeding. Agronomie.

[CR9] Bouchet S, Servin B, Bertin P (2013). Adaptation of maize to temperate climates: mid-density genome-wide association genetics and diversity patterns Reveal key genomic regions, with a major contribution of the Vgt2 (ZCN8) locus. PLoS ONE.

[CR10] Bouchet S, Bertin P, Presterl T (2017). Association mapping for phenology and plant architecture in maize shows higher power for developmental traits compared with growth influenced traits. Heredity.

[CR11] Bradbury PJ, Zhang Z, Kroon DE (2007). TASSEL: software for association mapping of complex traits in diverse samples. Bioinformatics.

[CR12] Brandenburg J-T, Mary-Huard T, Rigaill G (2017). Independent introductions and admixtures have contributed to adaptation of European maize and its American counterparts. PLOS Genet.

[CR13] Broman KW, Gatti DM, Simecek P (2019). R/qtl2: software for mapping quantitative trait loci with high-dimensional data and multiparent populations. Genetics.

[CR14] Browning BL, Browning SR (2009). A unified approach to genotype imputation and haplotype-phase inference for large data sets of trios and unrelated individuals. Am J Hum Genet.

[CR15] Bruce AB (1910). The Mendelian theory of heredity and the augmentation of vigor. Science.

[CR16] Buckler ES, Holland JB, Bradbury PJ (2009). The genetic architecture of maize flowering time. Science.

[CR17] Castelletti S, Tuberosa R, Pindo M, Salvi S (2014). A MITE transposon insertion is associated with differential methylation at the maize flowering time QTL Vgt1. G3 Genes Genomes Genet.

[CR18] Ceballos H, Pandey S, Narro L, Perez-Velázquez JC (1998). Additive, dominant, and epistatic effects for maize grain yield in acid and non-acid soils. Theor Appl Genet.

[CR19] Charcosset A, Causse M, Moreau L, Gallais A (1994) Investigation into the effect of genetic background on QTL expression using three connected maize recombinant inbred lines (RIL) populations. In: Biometrics in plant breeding: applications of molecular markers: Proceedings of the 9th Meeting of the Eucarpia Section Biometrics in Plant Breeding. pp 75–84

[CR20] Chardon F, Virlon B, Moreau L (2004). Genetic architecture of flowering time in maize as inferred from quantitative trait loci meta-analysis and synteny conservation with the rice genome. Genetics.

[CR21] Crow JF (1948). Alternative hypotheses of hybrid vigor. Genetics.

[CR22] Crow JF (1999) Dominance and Overdominance. In: Genetics and Exploitation of Heterosis in Crops. John Wiley & Sons, Ltd, pp 49–58

[CR23] Da Y, Wang C, Wang S, Hu G (2014). Mixed model methods for genomic prediction and variance component estimation of additive and dominance effects using SNP markers. PLoS ONE.

[CR24] Darwin C (1876). The effects of cross and self fertilisation in the vegetable kingdom: by Charles Darwin.

[CR25] Davenport CB (1908) Degeneration, Albinism and Inbreeding. Science 10.1126/science.28.718.454-b 17771943

[CR26] Deng H-W (2001). Population admixture may appear to mask, change or reverse genetic effects of genes underlying complex traits. Genetics.

[CR27] Dias KODG, Gezan SA, Guimarães CT (2018). Improving accuracies of genomic predictions for drought tolerance in maize by joint modeling of additive and dominance effects in multi-environment trials. Heredity.

[CR28] Dubreuil P, Warburton ML, Chastanet M, et al (2006) More on the introduction of temperate maize into Europe: large-scale bulk SSR genotyping and new historical elements

[CR29] Ducrocq S, Madur D, Veyrieras J-B (2008). Key impact of Vgt1 on flowering time adaptation in maize: evidence from association mapping and ecogeographical information. Genetics.

[CR30] Durand E, Bouchet S, Bertin P (2012). Flowering time in maize: linkage and epistasis at a major effect locus. Genetics.

[CR31] East EM (1908). Inbreeding in corn. Rep Conn Agric Exp Stn.

[CR32] Endelman JB (2011). Ridge Regression and Other Kernels for Genomic Selection with R Package rrBLUP. Plant Genome.

[CR33] Evangelou E, Ioannidis JPA (2013). Meta-analysis methods for genome-wide association studies and beyond. Nat Rev Genet.

[CR34] Falconer DS, Mackay TFC, Frankham R (1996). Introduction to quantitative genetics.

[CR35] Fisher RA (1918). XV.—The correlation between relatives on the supposition of Mendelian inheritance. Earth Environ Sci Trans R Soc Edinb.

[CR36] Ganal MW, Durstewitz G, Polley A (2011). A large maize (*Zea mays* L.) SNP genotyping array: development and germplasm genotyping, and genetic mapping to compare with the B73 reference genome. PLoS ONE.

[CR37] Gerke JP, Edwards JW, Guill KE (2015). The genomic impacts of drift and selection for hybrid performance in maize. Genetics.

[CR38] Giraud H, Lehermeier C, Bauer E (2014). Linkage disequilibrium with linkage analysis of multiline crosses reveals different multiallelic QTL for hybrid performance in the flint and dent heterotic groups of maize. Genetics.

[CR39] González-Diéguez D, Legarra A, Charcosset A (2021). Genomic prediction of hybrid crops allows disentangling dominance and epistasis. Genetics.

[CR40] Graham GI, Wolff DW, Stuber CW (1997). Characterization of a yield quantitative trait locus on chromosome five of maize by fine mapping. Crop Sci.

[CR100] Guo T, Li H, Yan J, Tang J, Li J, Zhang Z, Zhang L, Wang J (2013). Performance prediction of F1 hybrids between
recombinant inbred lines derived from two elite maize inbred lines. Theor Appl Genet.

[CR41] Hill WG, Weir BS (1988). Variances and covariances of squared linkage disequilibria in finite populations. Theor Popul Biol.

[CR42] Huang W, Mackay TFC (2016). The genetic architecture of quantitative traits cannot be inferred from variance component analysis. PLOS Genet.

[CR43] Hull FH (1945). Recurrent selection for specific combining ability in corn1. Agron J.

[CR44] Hull FH (1946). Overdominance and corn breeding where hybrid seed is not feasible. Agron J.

[CR45] Hunter RB, Mortimore G, Gerrish EE, Kannenberg LW (1979). Field drying of flint and dent endosperm maize1. Crop Sci.

[CR46] Ioannidis JPA, Ntzani EE, Trikalinos TA (2004). “Racial” differences in genetic effects for complex diseases. Nat Genet.

[CR47] Jannink J-L, Jansen R (2001). Mapping epistatic quantitative trait loci with one-dimensional genome searches. Genetics.

[CR48] Jinks JL, Jones RM (1958). Estimation of the components of heterosis. Genetics.

[CR49] Jones DF (1917). Dominance of linked factors as a means of accounting for heterosis. Genetics.

[CR50] Lamkey KR, Edwards JW (1999). Quantitative genetics of heterosis. Genet Exploit Heterosis Crops.

[CR51] Lamkey CM, Lorenz AJ (2014). Relative effect of drift and selection in diverging populations within a reciprocal recurrent selection program. Crop Sci.

[CR52] Laporte F, Charcosset A, Mary-Huard T (2022). Efficient ReML inference in variance component mixed models using a Min-Max algorithm. PLOS Comput Biol.

[CR53] Larièpe A, Mangin B, Jasson S (2012). The genetic basis of heterosis: multiparental quantitative trait loci mapping reveals contrasted levels of apparent overdominance among traits of agronomical interest in maize (*Zea mays* L.). Genetics.

[CR54] Li YR, Keating BJ (2014). Trans-ethnic genome-wide association studies: advantages and challenges of mapping in diverse populations. Genome Med.

[CR55] Lippman ZB, Zamir D (2007). Heterosis: revisiting the magic. Trends Genet.

[CR56] Lynch M, Walsh B (1998). Genetics and analysis of quantitative traits.

[CR57] Maher B (2008). The case of the missing heritability: when scientists opened up the human genome, they expected to find the genetic components of common traits and diseases. But they were nowhere to be seen. Brendan Maher shines a light on six places where the missing loot could be stashed away. Nature.

[CR58] Marigorta UM, Navarro A (2013). High trans-ethnic replicability of GWAS results implies common causal variants. PLOS Genet.

[CR59] Mayer M, Hölker AC, González-Segovia E (2020). Discovery of beneficial haplotypes for complex traits in maize landraces. Nat Commun.

[CR60] Melchinger AE, Gumber RK (1998) Overview of Heterosis and Heterotic Groups in Agronomic Crops. In: Concepts and Breeding of Heterosis in Crop Plants. John Wiley & Sons, Ltd, pp 29–44

[CR61] Negro SS, Millet EJ, Madur D (2019). Genotyping-by-sequencing and SNP-arrays are complementary for detecting quantitative trait loci by tagging different haplotypes in association studies. BMC Plant Biol.

[CR62] Ntzani EE, Liberopoulos G, Manolio TA, Ioannidis JPA (2012). Consistency of genome-wide associations across major ancestral groups. Hum Genet.

[CR63] Pasaniuc B, Sankararaman S, Torgerson DG (2013). Analysis of Latino populations from GALA and MEC studies reveals genomic loci with biased local ancestry estimation. Bioinformatics.

[CR64] Peiffer JA, Romay MC, Gore MA (2014). The genetic architecture of maize height. Genetics.

[CR65] Powers L (1944). An Expansion of Jones’s theory for the explanation of heterosis. Am Nat.

[CR66] Rebaï A, Blanchard P, Perret D, Vincourt P (1997). Mapping quantitative trait loci controlling silking date in a diallel cross among four lines of maize. Theor Appl Genet.

[CR67] Rebai A, Goffinet B (1993). Power of tests for QTL detection using replicated progenies derived from a diallel cross. Theor Appl Genet.

[CR68] Rebourg C, Chastanet M, Gouesnard B (2003). Maize introduction into Europe: the history reviewed in the light of molecular data. Theor Appl Genet.

[CR69] Reif JC, Gumpert F-M, Fischer S, Melchinger AE (2007). Impact of interpopulation divergence on additive and dominance variance in hybrid populations. Genetics.

[CR70] Richey FD (1942). Mock-dominance and hybrid vigor. Science.

[CR71] Rincent R, Moreau L, Monod H (2014). Recovering power in association mapping panels with variable levels of linkage disequilibrium. Genetics.

[CR72] Rincent R, Nicolas S, Bouchet S (2014). Dent and Flint maize diversity panels reveal important genetic potential for increasing biomass production. Theor Appl Genet.

[CR73] Rio S, Mary-Huard T, Moreau L, Charcosset A (2019). Genomic selection efficiency and a priori estimation of accuracy in a structured dent maize panel. Theor Appl Genet.

[CR74] Rio S, Mary-Huard T, Moreau L (2020). Disentangling group specific QTL allele effects from genetic background epistasis using admixed individuals in GWAS: An application to maize flowering. PLOS Genet.

[CR75] Rogers AR, Dunne JC, Romay C (2021). The importance of dominance and genotype-by-environment interactions on grain yield variation in a large-scale public cooperative maize experiment. G3 GenesGenomesGenetics.

[CR76] Romay MC, Millard MJ, Glaubitz JC (2013). Comprehensive genotyping of the USA national maize inbred seed bank. Genome Biol.

[CR77] Roth M, Beugnot A, Mary-Huard T, et al (2022) Improving genomic predictions with inbreeding and non-additive effects in two admixed maize hybrid populations in single and multi-environment contexts. Genetics iyac018. 10.1093/genetics/iyac018 10.1093/genetics/iyac018 PMC8982028 35150258

[CR78] Salvi S, Sponza G, Morgante M (2007). Conserved noncoding genomic sequences associated with a flowering-time quantitative trait locus in maize. Proc Natl Acad Sci.

[CR79] Shull GH (1914). Duplicate genes for capsule form in Bursa pastoris Zeitscher. Induktica Abstammu Vererbunglehra.

[CR80] Shull GH (1908) The Composition of a Field of Maize. J Hered os-4:296–301. 10.1093/jhered/os-4.1.296

[CR81] Skotte L, Jørsboe E, Korneliussen TS (2019). Ancestry-specific association mapping in admixed populations. Genet Epidemiol.

[CR82] Su G, Christensen OF, Ostersen T, et al (2012) Estimating Additive and Non-Additive Genetic Variances and Predicting Genetic Merits Using Genome-Wide Dense Single Nucleotide Polymorphism Markers. PLOS ONE 7:e45293. 10.1371/journal.pone.0045293 10.1371/journal.pone.0045293 PMC3441703 23028912

[CR83] Tang H, Siegmund DO, Johnson NA (2010). Joint testing of genotype and ancestry association in admixed families. Genet Epidemiol.

[CR84] Tenaillon MI, Charcosset A (2011). A European perspective on maize history. C R Biol.

[CR85] Tian F, Bradbury PJ, Brown PJ (2011). Genome-wide association study of leaf architecture in the maize nested association mapping population. Nat Genet.

[CR86] Unterseer S, Bauer E, Haberer G (2014). A powerful tool for genome analysis in maize: development and evaluation of the high density 600 k SNP genotyping array. BMC Genomics.

[CR87] Unterseer S, Pophaly SD, Peis R (2016). A comprehensive study of the genomic differentiation between temperate Dent and Flint maize. Genome Biol.

[CR88] Vitezica ZG, Varona L, Legarra A (2013). On the Additive and Dominant Variance and Covariance of Individuals Within the Genomic Selection Scope. Genetics.

[CR89] Vitezica ZG, Legarra A, Toro MA, Varona L (2017). Orthogonal Estimates of Variances for Additive, Dominance, and Epistatic Effects in Populations. Genetics.

[CR90] Waters KM, Stram DO, Hassanein MT, et al (2010) Consistent Association of Type 2 Diabetes Risk Variants Found in Europeans in Diverse Racial and Ethnic Groups. PLOS Genet 6: e1001078. 10.1371/journal.pgen.1001078 10.1371/journal.pgen.1001078 PMC2928808 20865176

[CR91] Williams W (1960). Heterosis and the genetics of complex characters. Heredity.

[CR92] Williams RC, Long JC, Hanson RL (2000). Individual Estimates of European Genetic Admixture Associated with Lower Body-Mass Index, Plasma Glucose, and Prevalence of Type 2 Diabetes in Pima Indians. Am J Hum Genet.

[CR93] Wyss AB, Sofer T, Lee MK (2018). Multiethnic meta-analysis identifies ancestry-specific and cross-ancestry loci for pulmonary function. Nat Commun.

[CR94] Xiao Y, Liu H, Wu L (2017). Genome-wide Association Studies in Maize: Praise and Stargaze. Mol Plant.

[CR95] Zdunic Z, Mijic A, Dugalic K (2008). Genetic Analysis of Grain Yield and Starch Content in Nine Maize Populations. Turk J Agric for.

[CR96] Zhang J, Stram DO (2014). The Role of Local Ancestry Adjustment in Association Studies Using Admixed Populations. Genet Epidemiol.

