# Supplementary material for: Identifying QTLs involved in hybrid performance and heterotic group complementarity: new GWAS models applied to factorial and admixed diallel maize hybrid panels
Source: Theor Appl Genet. 2023 Oct 10;136(11):219. doi: 10.1007/s00122-023-04431-w (PMC10564676; doi:10.1007/s00122-023-04431-w)
Supplement: Supplementary file 1 — Supplementary file1 (DOCX 1667 KB) [file 122_2023_4431_MOESM1_ESM.docx]

**Theoretical and Applied Genetics**

**Identifying QTLs involved in hybrid performance and heterotic group complementarity: new GWAS models applied to factorial and admixed diallel maize hybrid panels**

Aurélien Beugnot^1&^, Tristan Mary-Huard^1,2&^, Cyril Bauland^1^, Valerie Combes^1^, Delphine Madur^1^, Bernard Lagardère^3^, Carine Palaffre^3^, Alain Charcosset^1&^, Laurence Moreau^1&^, Julie B. Fievet^1*&^

1. Université Paris-Saclay, INRAE, CNRS, AgroParisTech, UMR GQE-Le Moulon, 91272 Gif-sur-Yvette, France
2. INRAE, AgroParisTech, UMR MIA-Paris, 75005, Paris, France

3. INRAE, UE 0394, 40390, Saint Martin De Hinx, France.

* Corresponding author e-mail: julie.fievet@inrae.fr

**APPENDIX**

## S1 Appendix: Spatial correction model for Het2 by environment

Local spatial effects were corrected in each Het2 environment using the following model:

$$\begin{matrix} Y_{htrc} & = & \mu+\lambda_{t}+R_{r}+C_{c}+G_{h}+E_{htrc} \\ R & \sim& N(0,I\sigma_{R}^{2}) \\ C & \sim& N(0,{I\sigma}_{C}^{2}) \\ G & \sim& N(0,I\sigma_{G}^{2}) \\ E & \sim& N(0,I\sigma_{\varepsilon}^{2}) \\ & & R\perp C\perp G\perp E \end{matrix}$$

where $Y_{htrc}$ is the phenotype of hybrid *h* with check status *t*, at row *r*, column *c*,$\mu$ is the intercept, $\lambda_{t}$ is the effect of check *t* (including 5 levels, one for each of the 4 checks and 1 for experimental hybrids), $R_{R}$ is the random effect of row *r* with $\sigma_{R}^{2}$ the row variance and $C_{c}$ is the random effect of column *c* with $\sigma_{C}^{2}$ the column variance, $G_{h}$ is the random effect of genotype *h* with $\sigma_{G}^{2}$ the genotypic variance and $E$ is a vector for the error with $\sigma_{\varepsilon}^{2}$ the error variance.

## S2 Appendix: Broad Sense Heritability

Broad sense heritability was calculated based on a single environment genetic model on corrected phenotypes:

$$Y_{hk}=\mu+G_{h}+E_{hk}$$

$$G\sim N\left( 0,I\sigma_{G}^{2} \right), E\sim N\left( 0,I\sigma_{\varepsilon}^{2} \right), G\perp E$$

where $Y_{hk}$ is the *k^th^* phenotype measure of hybrid *h*,$\mu$ is the intercept, $G_{h}$ is the random effect of genotype *h* with $\sigma_{G}^{2}$ the genotypic variance and $E$ is a vector for the error with $\sigma_{\varepsilon}^{2}$ the error variance. Single environment heritability is given by the following formula:

$$H_{S}^{2}=\frac{\sigma_{G}^{2}}{\sigma_{G}^{2}+\frac{\sigma_{\varepsilon}^{2}}{n_{rep}}}$$

where $\sigma_{G}^{2}$ and $\sigma_{\varepsilon}^{2}$ are described above and $n_{rep}$ is the mean number of repeated values.

In addition, broad sense heritability was calculated based on multi-environment genetic model:

$$Y_{hek}=\mu+\theta_{e}+G_{h}+{GE}_{he}+E_{hek}$$

$$G\sim N\left( 0,I\sigma_{G}^{2} \right),GE\sim N\left( 0,I\sigma_{GE}^{2} \right), E\sim N\left( 0,I\sigma_{\varepsilon}^{2} \right), G\perp GE\perp E$$

where $Y_{hek}$ is the *k^th^* phenotype measure of hybrid *h* in the environment *e*,$\mu$ is the intercept, $\theta_{e}$ is the effect of the environment *e*, $G_{h}$ is the random effect of genotype *h* with $\sigma_{G}^{2}$ the genotypic variance, ${GE}_{h}$ is the random effect of genotype-by-environment interactions with variance $\sigma_{GE}^{2}$ and $E$ is the vector for the error with $\sigma_{\varepsilon}^{2}$ is error variance.

Multi environment heritability was defined as follows:

$$H^{2}=\frac{\sigma_{G}^{2}}{\sigma_{G}^{2}+\frac{\sigma_{GE}^{2}}{n_{env}}+\frac{\sigma_{\varepsilon}^{2}}{n_{rep}*n_{env}}}$$

where $\sigma_{G}^{2},\sigma_{GE}^{2}$ and $\sigma_{\varepsilon}^{2}$ are described above, $n_{env}$ is the number of environment and $n_{rep}$ is the mean number of replicates per trial.

## S3 Appendix: Variance estimation procedure in GWAS

GWAS models requires the inference of numerous variance parameters. Fitting such models for each marker would require a prohibitive computation time. A 2-step procedure has been suggested to reduce this issue. In a first step, variance parameters were estimated in a $H_{0}$ model including no marker effect, corresponding to the MONO model of “Variance partition” section:

$$Y=1\mu+Z^{A}A+Z^{D}D+ Z^{I^{aa}}I^{aa}+ Z^{I^{ad}}I^{ad}+ Z^{I^{dd}}I^{dd}+E$$

$$A\sim N\left( 0, K_{a}\sigma_{a}^{2} \right) , D\sim N\left( 0, K_{d}\sigma_{d}^{2} \right) ,$$

$$I^{aa}\sim N\left( 0, K_{aa}\sigma_{aa}^{2} \right), I^{ad}\sim N\left( 0, K_{ad}\sigma_{ad}^{2} \right) , I^{dd}\sim N\left( 0, K_{dd}\sigma_{dd}^{2} \right)$$

$$E\sim N(0, I\sigma_{\varepsilon}^{2})$$

where $Y$ is the vector of phenotypic values,$\mu$ is the intercept, $A$, $D$, $I^{aa}$,$I^{ad}$ and $I^{dd}$ are vectors of random effects for additivity, dominance and epistasis with incidence matrices $Z^{A}$, $Z^{D}$, $Z^{I^{aa}}$, $Z^{I^{ad}}$ and $Z^{I^{dd}}$ and variance$\sigma_{a}^{2}$, $\sigma_{d}^{2}$, $\sigma_{aa}^{2}$, $\sigma_{ad}^{2}$ and $\sigma_{dd}^{2}$ respectively, $K_{a}$, $K_{d}$, $K_{aa}$, $K_{ad}$ and $K_{dd}$ are kinship matrices of those effects and E is a vector for the error of variance$\sigma_{\varepsilon}^{2}$. The phenotypic covariance matrix ${V(Y)}_{SE}$ can be expressed as:

$${V(Y)}_{SE}=\sigma_{a}^{2}Z^{A}K_{a}{Z^{A}}^{T}+{\sigma_{d}^{2}Z}^{D}K_{d}{Z^{D}}^{T}+{\sigma_{aa}^{2}Z}^{I^{aa}}K_{aa}{Z^{I^{aa}}}^{T}+\sigma_{ad}^{2}Z^{I^{ad}}K_{ad}{Z^{I^{ad}}}^{T}+{\sigma_{dd}^{2}Z}^{I^{dd}}K_{dd}{Z^{I^{dd}}}^{T}+\sigma_{\varepsilon}^{2}I$$

In a second step, the phenotypic covariance was considered as known in the marker-by-marker GWAS model. A “all-in-one” model was at marker *m*:

$$Y=1\mu+X\beta+E$$

$$E\sim N(0, {V(Y)}_{SE})$$

where $Y$ is the vector of phenotypic values, $\mu$ is the intercept, $X$ is the incidence matrix of SO genotypes at marker m and $\beta$ is the vector of parameters for SO genotypes, $E$ is a vector of effects accounting for polygenic and environment effects with covariance matrix ${V(Y)}_{SE}$.

This simplification reduced drastically the number of estimated variances but decreased the detection power of the GWAS. We applied an adapted version of this procedure to give more flexibility for fitting the model during the marker-by-marker analysis. We decomposed the phenotypic covariance matrix into three terms (additive, non-additive and residual effects) and considered it as partially known.

Then, the phenotypic covariance matrix was:

$${V(Y)}_{SE}=\lambda_{A}\left[ \sigma_{a}^{2}Z^{A}K_{a}{Z^{A}}^{T} \right]+\lambda_{DI}\left[ {\sigma_{d}^{2}Z}^{D}K_{d}{Z^{D}}^{T}+{\sigma_{aa}^{2}Z}^{I^{aa}}K_{aa}{Z^{I^{aa}}}^{T}+\sigma_{ad}^{2}Z^{I^{ad}}K_{ad}{Z^{I^{ad}}}^{T}+{\sigma_{dd}^{2}Z}^{I^{dd}}K_{dd}{Z^{I^{dd}}}^{T} \right]+\lambda_{E}\left[ \sigma_{\varepsilon}^{2}I \right]$$

or in a simpler way:

${V(Y)}_{SE}=\lambda_{A}\left[ {V\left( Y \right)}_{SE}^{A} \right]+\lambda_{DI}\left[ {V\left( Y \right)}_{SE}^{DI} \right]+\lambda_{E}\left[ {V\left( Y \right)}_{SE}^{E} \right]$.

where $\lambda_{A}$, $\lambda_{DI}$ and $\lambda_{E}$ are fitting parameters for additive, non-additive and residual terms.

We performed a GWAS using the following model:

At marker *m*: $Y=1\mu+X\beta+I^{n}A+I^{n}DI+ I^{n}E$

$A\sim N\left( 0, {\lambda_{A}V\left( Y \right)}_{SE}^{A} \right)$, $DI\sim N\left( 0,\lambda_{DI} {V\left( Y \right)}_{SE}^{DI} \right)$, $E\sim N(0, {\lambda_{E}V\left( Y \right)}_{SE}^{E})$

where $X$ is the incidence matrix of SO genotypes at marker *m*, $\beta$ is the vector of parameter for SO genotypes, $I^{n}$ is an identity matrix of dimension n-by-n, n being the number of observations, $A$ and $DI$ is a random effect accounting for additive and non-additive polygenic effect, $E$ is a vector of effects accounting for covariance due to environment effects, $\lambda_{A}$, $\lambda_{DI}$ and $\lambda_{E}$ are estimated for each marker which results in a better fitting of the model than the “all-in-one” procedure.

Splitting phenotypic covariance into three terms is a reasonable trade-off between full GWAS model (Gso-MONO model) and the “all-in-one” procedure. A gain in detection power were shown in comparison with the “all-in-one” procedure, leading to the identification of new candidate QTL whereas strongly decrease the computational times of the full model (result not shown). This strategy was applied with success as well in previous article (Rio S 2019).

A similar partition of the phenotypic covariance was applied for **MULTI** model:

$$Y=1\mu+W\theta+Z^{A}A+Z^{D}D+ Z^{I^{aa}}I^{aa}+ Z^{I^{ad}}I^{ad}+ Z^{I^{dd}}I^{dd}+\sum_{e} \left[ Z_{e}^{AE}{AE}_{e}+Z_{e}^{DE}{DE}_{e}+Z_{e}^{E}E_{e} \right]$$

$$A\sim N\left( 0, K_{a}\sigma_{a}^{2} \right) , D\sim N\left( 0, K_{d}\sigma_{d}^{2} \right) ,$$

$$I^{aa}\sim N\left( 0, K_{aa}\sigma_{aa}^{2} \right), I^{ad}\sim N\left( 0, K_{ad}\sigma_{ad}^{2} \right) , I^{dd}\sim N\left( 0, K_{dd}\sigma_{dd}^{2} \right)$$

$${AE}_{e}\sim N\left( 0, K_{a}\sigma_{a(e)}^{2} \right) IND,{DE}_{e}\sim N\left( 0, K_{d}\sigma_{d(e)}^{2} \right) IND$$

$$E_{e}\sim N(0, I\sigma_{\varepsilon(e)}^{2})$$

where $W$ is the incidence matrix of environmental effects and $\theta$ is the vector of environmental parameters, ${AE}_{e}$, ${DE}_{e}$ and $E_{e}$ are vectors of random effects for environment specific additivity, dominance and error effects respectively, with environment specific variances$\sigma_{a(e)}^{2}$, $\sigma_{d(e)}^{2}$ and $\sigma_{\varepsilon(e)}^{2}$. $E_{e}$ is a vector for environment specific error with variance $\sigma_{\varepsilon(e)}^{2}$. Other terms were equivalent to the MONO decomposition. The phenotypic covariance matrix can be expressed as:

$${V(Y)}_{ME}=\lambda_{A}\left[ Z^{A}K_{a}\sigma_{a}^{2}{Z^{A}}^{T}+\sum_{e} \left[ Z_{e}^{AE}K_{a}\sigma_{a(e)}^{2}{Z_{e}^{AE}}^{T} \right] \right]+\lambda_{DI}\left[ Z^{D}K_{d}\sigma_{d}^{2}{Z^{D}}^{T}+Z^{I^{aa}}K_{aa}\sigma_{aa}^{2}{Z^{I^{aa}}}^{T}+Z^{I^{ad}}K_{ad}\sigma_{ad}^{2}{Z^{I^{ad}}}^{T}+Z^{I^{dd}}K_{dd}\sigma_{dd}^{2}{Z^{I^{dd}}}^{T}+\sum_{e} \left[ Z_{e}^{DI}K_{d}\sigma_{d(e)}^{2}{Z_{e}^{DI}}^{T} \right] \right]+\lambda_{E}\left[ \sum_{e} \left[ Z_{e}^{E}\sigma_{\varepsilon(e)}^{2}{Z_{e}^{E}}^{T} \right] \right]$$

or:

${V(Y)}_{ME}=\lambda_{A}\left[ {V\left( Y \right)}_{ME}^{A} \right]+\lambda_{DI}\left[ {V\left( Y \right)}_{ME}^{DI} \right]+\lambda_{E}\left[ {V\left( Y \right)}_{ME}^{E} \right]$.

The GWAS model was:

At marker *m*: $Y=1\mu+W\alpha+X\beta+I^{n}A+I^{n}DI+ I^{n}E$

$A\sim N\left( 0, \lambda_{A}{V\left( Y \right)}_{SE}^{A} \right)$, $DI\sim N\left( 0, {\lambda_{DI}V\left( Y \right)}_{SE}^{DI} \right)$, $E\sim N(0, {\lambda_{E}V\left( Y \right)}_{SE}^{E})$,

where $X$ is the incidence matrix of SO genotypes at marker *m*, $\beta$ is the vector of parameter for SO genotypes, $I_{n}$ is an identity matrix of dimension n-by-n, n being the number of observations, $A$ and $DI$ are a random effect accounting for additive and non-additive residual polygenic effect. $E$ is a vector of effects accounting for covariance due to environment effects, $\lambda_{A}$, $\lambda_{DI}$ and $\lambda_{E}$ are fitting parameters for additive, non-additive and residual terms.

**FIGURES**


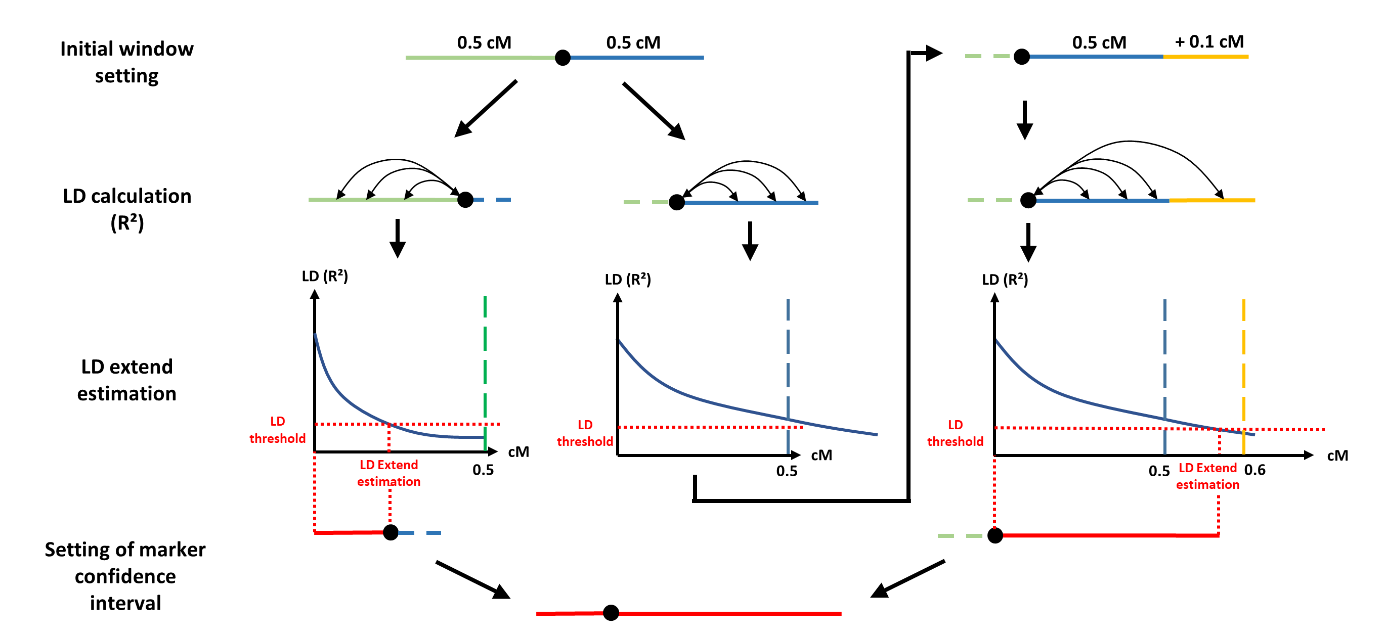


**S1 Fig. Estimation of the** confidence **interval around significant marker.** The confidence interval estimation starts with the definition of an arbitrary interval of 1 cM on both sides of the significant marker. LD (Linkage Disequilibrium) is calculated as a R² between the significant marker and the other markers within each interval using genotypic information. Then, LD is used to estimated LD extend with a threshold of 0.1. If the LD extend value is superior to the size of the interval, the interval size is increased by 0.1 cM. The procedure is repeated from the LD calculation until the LD extend reach a value inferior to the interval size. Lastly, the LD extend value is used to defined the limits of the confidence interval around the significant marker.


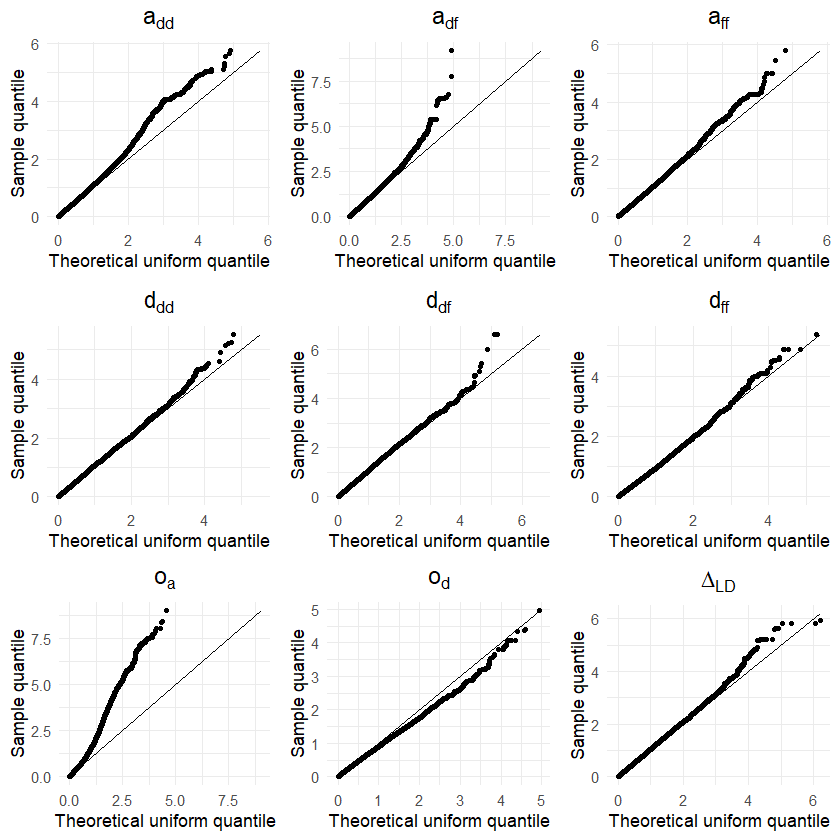


**S2 Fig.** Quantile-by-Quantile diagram of Pvalues for the Male Flowering **time (FloM) in JAR16 (Het2).** Black line represents the ideal distribution of the Pvalue according a uniform law.


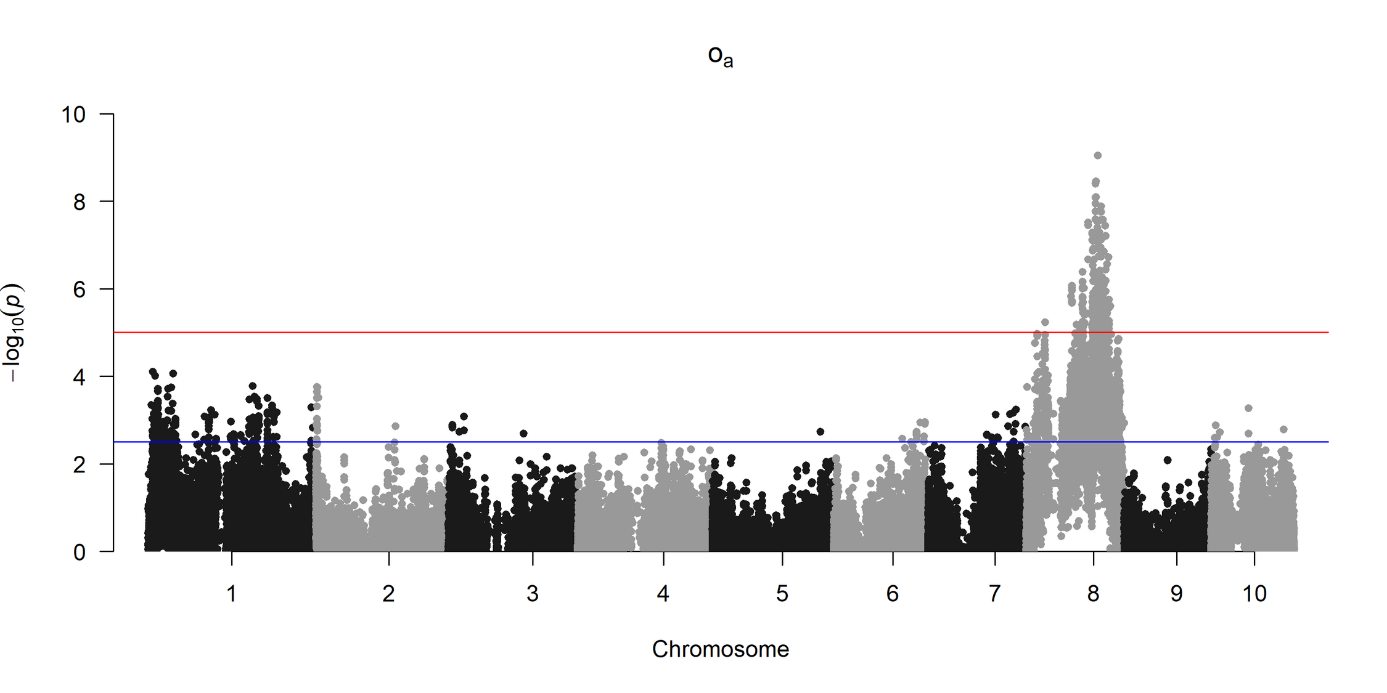


**S3 Fig. Manhattan plot for the Male flowering time (FloM) in JAR16 (Het2) for the additive effect of the origin (**$\boldsymbol{o}_{\boldsymbol{a}}$**).** Blue and red lines represent the Pvalue threshold for FDR levels of 0.2 and 0.05.


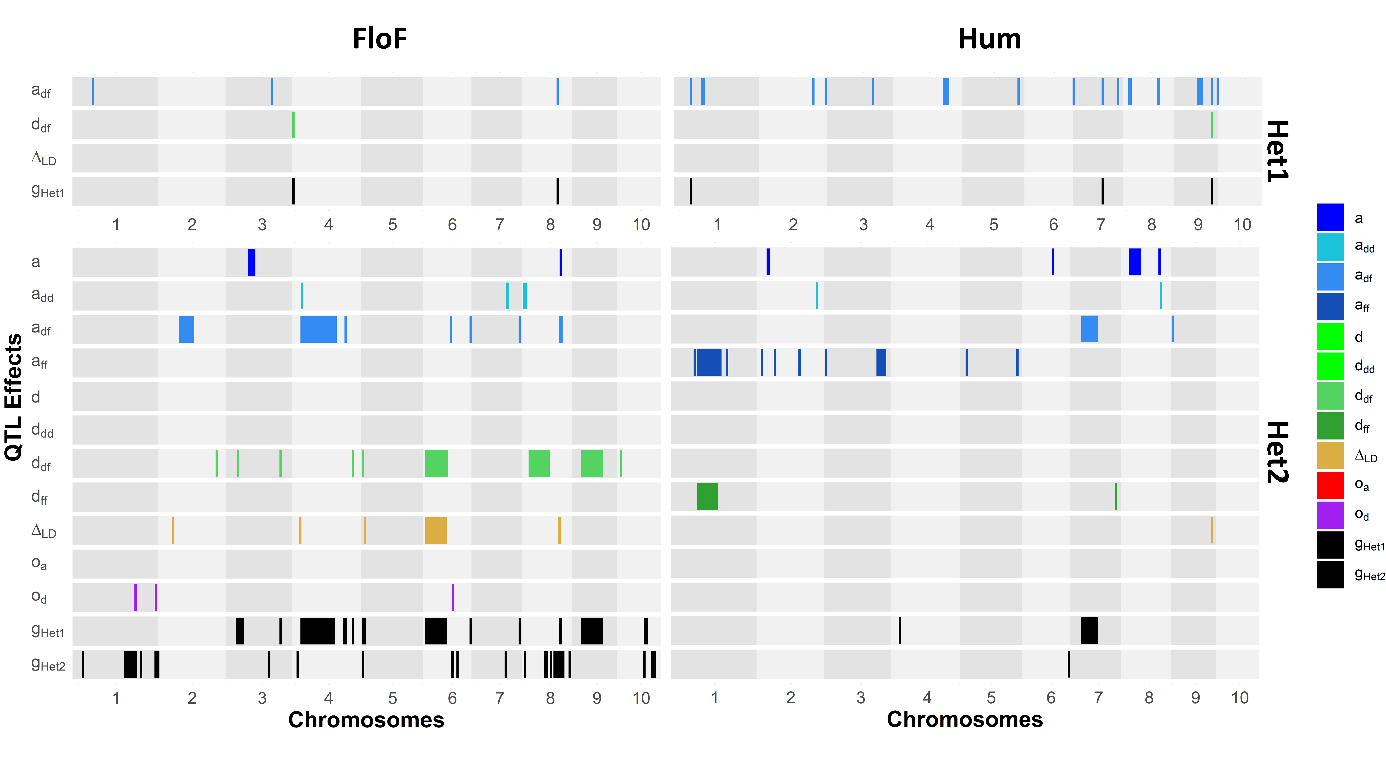


**S4 Fig. Representation of QTLs for the Female flowering (FloF) and the grain moisture (Hum) in the Het1 and Het2 panels.** Rectangles represent the QTL intervals. All results from the MONO and MULTI models were compiled for the Gso model. The nominal FDR level was fixed at 0.05.


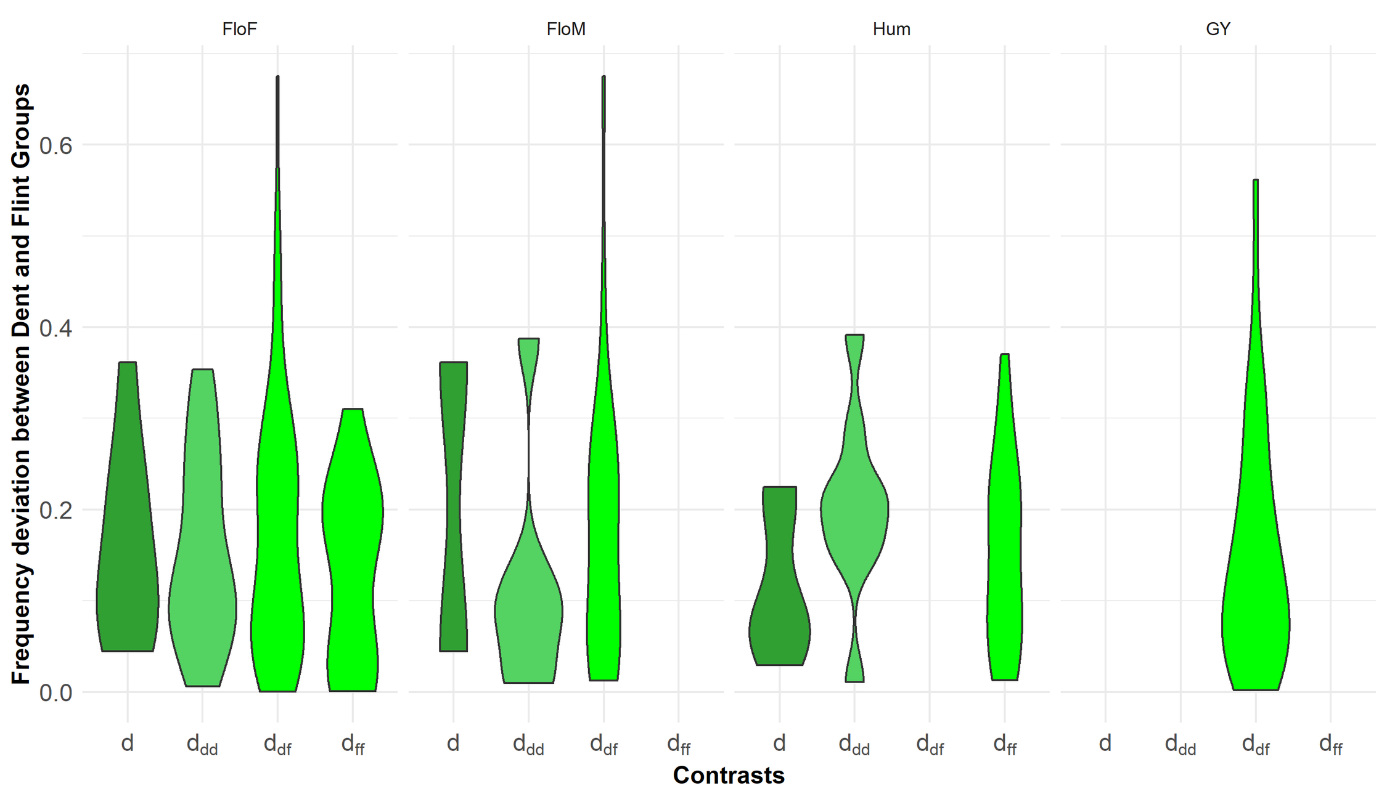


**S5 Fig. Differences between Dent and Flint allelic frequencies for the detected dominance QTLs in Het2.** Violin plot of the difference of SNP allelic frequencies between the Dent and the Flint group calculated as $Diff=\left| p_{D}-p_{F} \right|$ with $p_{D}$ and $p_{F}$ the Dent and Flint frequencies. The FDR nominal level is 0.2.


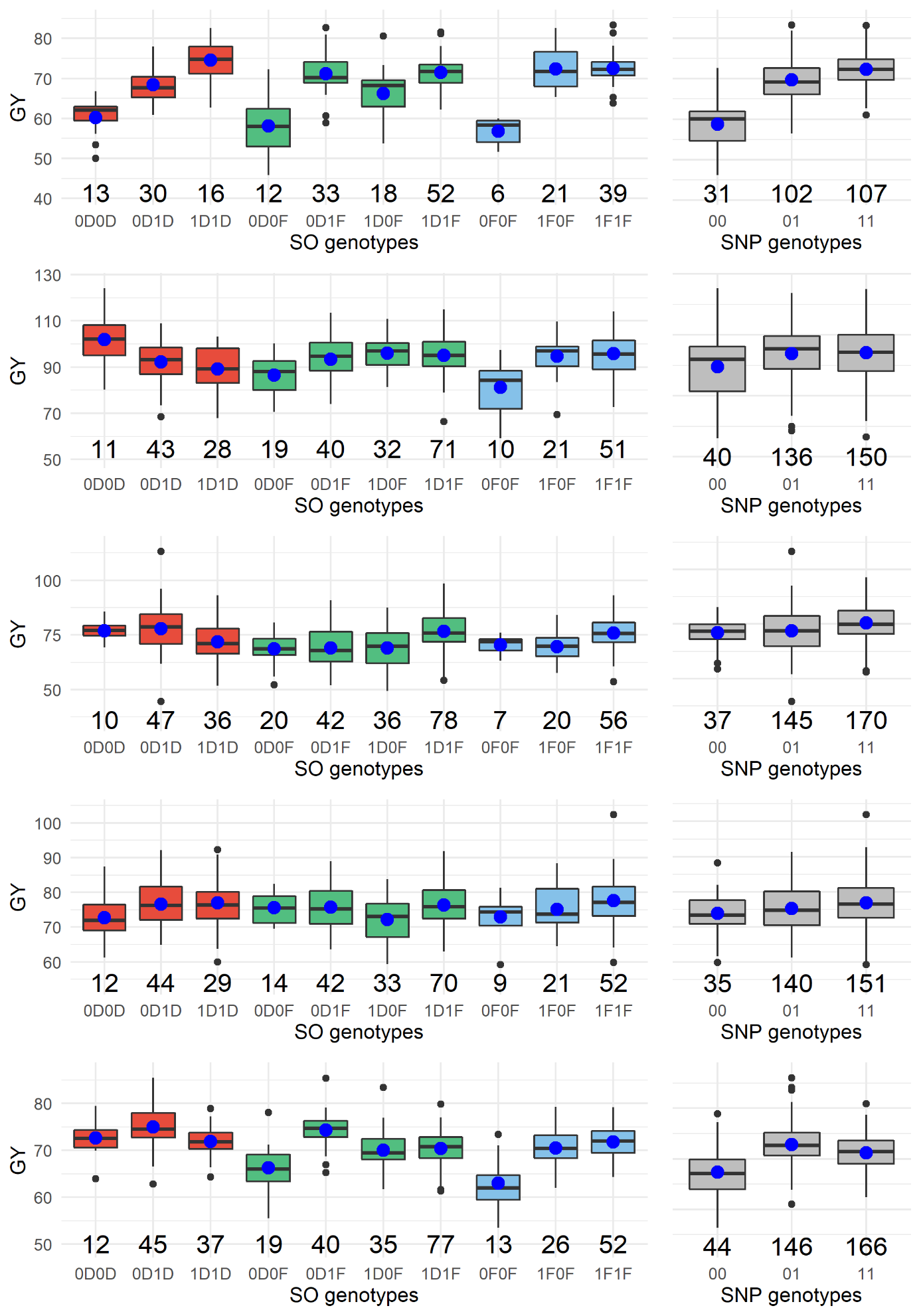


**S6 Fig. Illustration of the variation of additive effect in the dent genetic background across environments.** Boxplot of phenotype corrected by the kinship according to the Gso genotypes of marker AX-91203539 on chromosome 1 for the grain yield (GY) in all Het2 environment (Coloured boxplot; red, green and blue are for DD, DF and FF genetic backgrounds) and Gad model (Grey boxplot). The number at the bottom indicates the number of observations for each SO genotype.

**TABLES**

## S1 Table: Contrast matrix for $\boldsymbol{g}_{\boldsymbol{Het}\boldsymbol{1}}$ effect (global QTL based on Het1 SO genotypes)

|  | $\boldsymbol{\beta}_{\text{1D1D}}$ | $\boldsymbol{\beta}_{\text{0D0D}}$ | $\boldsymbol{\beta}_{\text{1F1F}}$ | $\boldsymbol{\beta}_{\text{0F0F}}$ | $\boldsymbol{\beta}_{\text{1D1F}}$ | $\boldsymbol{\beta}_{\text{0D0F}}$ | $\boldsymbol{\beta}_{\text{0D1D}}$ | $\boldsymbol{\beta}_{\text{1F0F}}$ | $\boldsymbol{\beta}_{\text{0D1F}}$ | $\boldsymbol{\beta}_{\text{1D0F}}$ |
| --- | --- | --- | --- | --- | --- | --- | --- | --- | --- | --- |
| $\boldsymbol{\beta}_{\mathbf{1D1F}}\boldsymbol{-}\boldsymbol{\beta}_{\boldsymbol{0}\boldsymbol{D}\boldsymbol{0}\boldsymbol{F}}=0$ |  |  |  |  | 1 | -1 |  |  |  |  |
| $\boldsymbol{\beta}_{\mathbf{1D1F}}\boldsymbol{-}\boldsymbol{\beta}_{\boldsymbol{0}\boldsymbol{D}\boldsymbol{1}\boldsymbol{F}}=0$ |  |  |  |  | 1 |  |  |  | -1 |  |
| $\boldsymbol{\beta}_{\mathbf{1D1F}}\boldsymbol{-}\boldsymbol{\beta}_{\boldsymbol{1}\boldsymbol{D}\boldsymbol{0}\boldsymbol{F}}=0$ |  |  |  |  | 1 |  |  |  |  | -1 |

Contrast matrix defined to test a global effect of a QTL based on SO genotypes present in Het1 panel. Empty spaces indicate a weight of 0. With $C$ the matrix (defined above) associating weights to the fixed parameters of the model, one can test: $H_{0}:\left\{ C\beta=0 \right\}$ vs $H_{1}:\left\{ C\beta\neq0 \right\}$.

## S2 Table: Contrast matrix for $\boldsymbol{g}_{\boldsymbol{Het}\boldsymbol{2}}$ effect (global QTL based on Het2 SO genotypes)

|  | $\boldsymbol{\beta}_{\text{1D1D}}$ | $\boldsymbol{\beta}_{\text{0D0D}}$ | $\boldsymbol{\beta}_{\text{1F1F}}$ | $\boldsymbol{\beta}_{\text{0F0F}}$ | $\boldsymbol{\beta}_{\text{1D1F}}$ | $\boldsymbol{\beta}_{\text{0D0F}}$ | $\boldsymbol{\beta}_{\text{0D1D}}$ | $\boldsymbol{\beta}_{\text{1F0F}}$ | $\boldsymbol{\beta}_{\text{0D1F}}$ | $\boldsymbol{\beta}_{\text{1D0F}}$ |
| --- | --- | --- | --- | --- | --- | --- | --- | --- | --- | --- |
| $\boldsymbol{\beta}_{\boldsymbol{1}\boldsymbol{D}\boldsymbol{1}\boldsymbol{D}}\boldsymbol{-}\boldsymbol{\beta}_{\boldsymbol{0}\boldsymbol{D}\boldsymbol{0}\boldsymbol{D}}=0$ | 1 | -1 |  |  |  |  |  |  |  |  |
| $\boldsymbol{\beta}_{\boldsymbol{1}\boldsymbol{D}\boldsymbol{1}\boldsymbol{D}}\boldsymbol{-}\boldsymbol{\beta}_{\boldsymbol{1}\boldsymbol{F}\boldsymbol{1}\boldsymbol{F}}=0$ | 1 |  | -1 |  |  |  |  |  |  |  |
| $\boldsymbol{\beta}_{\boldsymbol{1}\boldsymbol{D}\boldsymbol{1}\boldsymbol{D}}\boldsymbol{-}\boldsymbol{\beta}_{\boldsymbol{0}\boldsymbol{F}\boldsymbol{0}\boldsymbol{F}}=0$ | 1 |  |  | -1 |  |  |  |  |  |  |
| $\boldsymbol{\beta}_{\boldsymbol{1}\boldsymbol{D}\boldsymbol{1}\boldsymbol{D}}\boldsymbol{-}\boldsymbol{\beta}_{\boldsymbol{1}\boldsymbol{D}\boldsymbol{1}\boldsymbol{F}}=0$ | 1 |  |  |  | -1 |  |  |  |  |  |
| $\boldsymbol{\beta}_{\boldsymbol{1}\boldsymbol{D}\boldsymbol{1}\boldsymbol{D}}\boldsymbol{-}\boldsymbol{\beta}_{\boldsymbol{0}\boldsymbol{D}\boldsymbol{0}\boldsymbol{F}}=0$ | 1 |  |  |  |  | -1 |  |  |  |  |
| $\boldsymbol{\beta}_{\boldsymbol{1}\boldsymbol{D}\boldsymbol{1}\boldsymbol{D}}\boldsymbol{-}\boldsymbol{\beta}_{\boldsymbol{0}\boldsymbol{D}\boldsymbol{1}\boldsymbol{D}}=0$ | 1 |  |  |  |  |  | -1 |  |  |  |
| $\boldsymbol{\beta}_{\boldsymbol{1}\boldsymbol{D}\boldsymbol{1}\boldsymbol{D}}\boldsymbol{-}\boldsymbol{\beta}_{\boldsymbol{1}\boldsymbol{F}\boldsymbol{0}\boldsymbol{F}}=0$ | 1 |  |  |  |  |  |  | -1 |  |  |
| $\boldsymbol{\beta}_{\boldsymbol{1}\boldsymbol{D}\boldsymbol{1}\boldsymbol{D}}\boldsymbol{-}\boldsymbol{\beta}_{\boldsymbol{0}\boldsymbol{D}\boldsymbol{1}\boldsymbol{F}}=0$ | 1 |  |  |  |  |  |  |  | -1 |  |
| $\boldsymbol{\beta}_{\boldsymbol{1}\boldsymbol{D}\boldsymbol{1}\boldsymbol{D}}\boldsymbol{-}\boldsymbol{\beta}_{\boldsymbol{1}\boldsymbol{D}\boldsymbol{0}\boldsymbol{F}}=0$ | 1 |  |  |  |  |  |  |  |  | -1 |

Contrast matrix defined to test a global effect of a QTL based on SO genotypes present in Het2 panel. Empty spaces indicate a weight of 0. With $C$ the matrix (defined above) associating weights to the fixed parameters of the model, one can test: $H_{0}:\left\{ C\beta=0 \right\}$ vs $H_{1}:\left\{ C\beta\neq0 \right\}$.

## S3 Table: Summary of agronomic traits with single environment models (MONO models)

| **Panel** | **Trait** | **Env** | **Mean** | **Min** | **Max** | **Std. Dev.** | $\boldsymbol{\sigma}_{\boldsymbol{G}}^{\boldsymbol{2}}$ | $\boldsymbol{\sigma}_{\boldsymbol{\varepsilon}}^{\boldsymbol{2}}$ | $\boldsymbol{H}_{\boldsymbol{s}}^{\boldsymbol{2}}$ |
| --- | --- | --- | --- | --- | --- | --- | --- | --- | --- |
| Het1 | FloF | CAU14 | 196 | 184 | 209 | 5,11 | 25,4 | 0,399 | 0,987 |
|  |  | AUB15 | 194 | 186 | 208 | 3,81 | 13,3 | 1,53 | 0,915 |
|  |  | RHO15 | 197 | 186 | 211 | 4,21 | 18,3 | 0,479 | 0,98 |
|  |  | MOR14 | 204 | 197 | 215 | 3,45 | 11,3 | 0,493 | 0,965 |
|  |  | VIL15 | 199 | 189 | 209 | 3,7 | 13,7 | 0,516 | 0,971 |
|  |  | SMH14 | 199 | 192 | 209 | 3,61 | 12,7 | 0,458 | 0,971 |
|  |  | SMH15 | 198 | 189 | 210 | 3,7 | 13,4 | 0,46 | 0,975 |
|  | FloM | CAU14 | 193 | 182 | 207 | 4,64 | 20,8 | 0,224 | 0,991 |
|  |  | AUB15 | 191 | 183 | 205 | 3,57 | 11,7 | 1,51 | 0,905 |
|  |  | RHO15 | 198 | 188 | 213 | 4,7 | 23,1 | 0,42 | 0,986 |
|  |  | MOR14 | 203 | 200 | 214 | 2,56 | 6,1 | 0,44 | 0,944 |
|  |  | VIL15 | 197 | 188 | 208 | 3,51 | 12,4 | 0,398 | 0,975 |
|  |  | SMH14 | 198 | 189 | 210 | 3,14 | 9,64 | 0,387 | 0,968 |
|  |  | SMH15 | 195 | 186 | 205 | 3,7 | 12,6 | 0,821 | 0,954 |
|  | Hum | CAU14 | 25,7 | 15,5 | 39,5 | 3,76 | 12,4 | 1,64 | 0,902 |
|  |  | AUB15 | 30,6 | 18,4 | 40,4 | 3,76 | 13,1 | 1,55 | 0,912 |
|  |  | RHO15 | 33,5 | 17 | 42 | 4,11 | 16,3 | 0,666 | 0,969 |
|  |  | MOR14 | 29,6 | 19,3 | 38,7 | 2,89 | 6,23 | 2,17 | 0,778 |
|  |  | VIL15 | 29,9 | 19,4 | 41,1 | 3,44 | 11,3 | 0,543 | 0,963 |
|  |  | SMH14 | 25,5 | 17,4 | 38,9 | 3,28 | 9,78 | 0,991 | 0,923 |
|  |  | SMH15 | 28,4 | 21,2 | 42,5 | 3,12 | 8,72 | 1,26 | 0,903 |
|  | GY | CAU14 | 83,2 | 24,2 | 139 | 19,7 | 301 | 101 | 0,784 |
|  |  | AUB15 | 112 | 30,7 | 164 | 22,2 | 362 | 144 | 0,756 |
|  |  | RHO15 | 87,9 | 26,3 | 130 | 18,4 | 253 | 97,2 | 0,77 |
|  |  | MOR14 | 73,2 | 34,2 | 123 | 16,6 | 190 | 87,5 | 0,726 |
|  |  | VIL15 | 86,5 | 45,7 | 116 | 13,9 | 126 | 67,8 | 0,699 |
|  |  | SMH14 | 100 | 39,1 | 161 | 20 | 290 | 93,8 | 0,79 |
|  |  | SMH15 | 85,6 | 31,5 | 132 | 16,6 | 129 | 144 | 0,548 |
| Het2 | FloF | JAR16 | 206 | 197 | 218 | 4,43 | 18,1 | 0,651 | 0,972 |
|  |  | AUB17 | 195 | 188 | 209 | 3,93 | 14,8 | 0,662 | 0,967 |
|  |  | SOU17 | 187 | 178 | 197 | 3,19 | 8,53 | 1,81 | 0,861 |
|  |  | SMH16 | 197 | 190 | 208 | 3,01 | 8,57 | 0,907 | 0,924 |
|  |  | SMH17 | 194 | 187 | 204 | 3,47 | 10,5 | 0,83 | 0,941 |
|  | FloM | JAR16 | 204 | 195 | 218 | 3,71 | 12,6 | 0,507 | 0,969 |
|  |  | AUB17 | 193 | 186 | 207 | 3,35 | 10,1 | 1,13 | 0,921 |
|  |  | SOU17 | 185 | 177 | 195 | 2,91 | 6,3 | 2,27 | 0,785 |
|  |  | SMH16 | 196 | 190 | 207 | 2,83 | 7,29 | 1,01 | 0,903 |
|  |  | SMH17 | 192 | 186 | 200 | 2,9 | 7,6 | 0,561 | 0,945 |
|  | Hum | JAR16 | 30,4 | 21,7 | 41,4 | 3,63 | 11,3 | 1,8 | 0,888 |
|  |  | AUB17 | 28,5 | 19,5 | 39,6 | 4,2 | 16,3 | 1,86 | 0,919 |
|  |  | SOU17 | 29,8 | 12,5 | 38,6 | 3,59 | 10,9 | 2,58 | 0,848 |
|  |  | SMH16 | 21,1 | 16,5 | 31,5 | 2,1 | 2,47 | 1,98 | 0,614 |
|  |  | SMH17 | 19,9 | 16 | 28,4 | 2,21 | 4,62 | 0,296 | 0,952 |
|  | GY | JAR16 | 69,8 | 37,1 | 102 | 13,1 | 115 | 48,6 | 0,745 |
|  |  | AUB17 | 93,9 | 31,2 | 139 | 19,6 | 234 | 152 | 0,668 |
|  |  | SOU17 | 73,6 | 14,1 | 130 | 23,2 | 391 | 159 | 0,764 |
|  |  | SMH16 | 76,1 | 38,7 | 119 | 14 | 110 | 88,1 | 0,615 |
|  |  | SMH17 | 71,4 | 37,3 | 116 | 13,3 | 141 | 38,1 | 0,823 |

Min, Max, Mean, Std.Dev are the minimum, maximum, mean and standard deviation of corrected phenotypic values. The variances $\sigma_{G}^{2}$ and $\sigma_{\varepsilon}^{2}$ are respectively for genetic and error effects.$H_{s}^{2}$ is the single environment heritability.

## S4 Table: Summary of agronomic traits with multiple environment models (MULTI model)

| **Panel** | **Trait** | **Mean** | **Min** | **Max** | **Std. Dev.** | **Mean with correction by the checks** | $\boldsymbol{\sigma}_{\boldsymbol{G}}^{\boldsymbol{2}}$ | $\boldsymbol{\sigma}_{\boldsymbol{GE}}^{\boldsymbol{2}}$ | $\boldsymbol{\sigma}_{\boldsymbol{\varepsilon}}^{\boldsymbol{2}}$ | $\boldsymbol{H}_{\boldsymbol{M}}^{\boldsymbol{2}}$ |
| --- | --- | --- | --- | --- | --- | --- | --- | --- | --- | --- |
| Het1 | FloF | 198 | 184 | 215 | 4,95 | 197,5 | 14,3 | 1,5 | 0,613 | 0,98 |
|  | FloM | 197 | 182 | 214 | 5,27 | 195,8 | 12,3 | 1,46 | 0,622 | 0,977 |
|  | Hum | 29 | 15,5 | 42,5 | 4,36 | 28,97 | 8,63 | 2,34 | 1,29 | 0,945 |
|  | GY | 89,8 | 24,2 | 164 | 21,9 | 89,03 | 173 | 65,4 | 109 | 0,881 |
| Het2 | FloF | 195 | 178 | 218 | 7,2 | 196,2 | 10,3 | 1,76 | 1,03 | 0,951 |
|  | FloM | 194 | 177 | 218 | 6,99 | 194,6 | 7,75 | 0,965 | 1,16 | 0,951 |
|  | Hum | 25,8 | 12,5 | 41,4 | 5,57 | 25,08 | 6,56 | 2,37 | 1,75 | 0,894 |
|  | GY | 77,2 | 14,1 | 139 | 19,4 | 79,11 | 126 | 76 | 102 | 0,789 |

Min, Max, Mean, Std.Dev are the minimum, maximum, mean and standard deviation of corrected phenotypic values. “Mean with correction by the checks” is the phenotypic mean of all environments corrected by the value of the checks. The variances $\sigma_{G}^{2}$, $\sigma_{GE}^{2}$ and $\sigma_{\varepsilon}^{2}$ are respectively for genetic, genetic-by-environment interaction and error effects.$H_{M}^{2}$ is the multi environment heritability.

## S5 Table: Single environment variance partition with MONO model

| **Panel** | **Trait** | **Env** | $\boldsymbol{\sigma}_{\boldsymbol{a}}^{\boldsymbol{2}}$ | $\boldsymbol{\sigma}_{\boldsymbol{d}}^{\boldsymbol{2}}$ | $\boldsymbol{\sigma}_{\boldsymbol{aa}}^{\boldsymbol{2}}$ | $\boldsymbol{\sigma}_{\boldsymbol{ad}}^{\boldsymbol{2}}$ | $\boldsymbol{\sigma}_{\boldsymbol{dd}}^{\boldsymbol{2}}$ | $\boldsymbol{\sigma}_{\boldsymbol{\varepsilon}}^{\boldsymbol{2}}$ | ${\boldsymbol{\sigma}_{\boldsymbol{a}}^{\boldsymbol{2}}}/{\boldsymbol{V}\boldsymbol{(}\boldsymbol{G}\boldsymbol{)}}$ | ${\boldsymbol{\sigma}_{\boldsymbol{d}}^{\boldsymbol{2}}}/{\boldsymbol{V}\boldsymbol{(}\boldsymbol{G}\boldsymbol{)}}$ | ${\boldsymbol{(}\boldsymbol{\sigma}_{\boldsymbol{I}}^{\boldsymbol{2}}\boldsymbol{)}}/{\boldsymbol{V}\boldsymbol{(}\boldsymbol{G}\boldsymbol{)}}$ |
| --- | --- | --- | --- | --- | --- | --- | --- | --- | --- | --- | --- |
| Het1 | FloF | CAU14 | 17,34 | 0,00 | 0,00 | 0,97 | 0,48 | 0,40 | 0,92 | 0,00 | 0,08 |
|  |  | AUB15 | 8,86 | 0,01 | 0,00 | 0,08 | 0,73 | 1,45 | 0,92 | 0,00 | 0,08 |
|  |  | RHO15 | 11,80 | 0,13 | 0,02 | 0,99 | 0,83 | 0,47 | 0,86 | 0,01 | 0,13 |
|  |  | MOR14 | 8,03 | 0,14 | 0,17 | 0,16 | 0,13 | 0,49 | 0,93 | 0,02 | 0,05 |
|  |  | VIL15 | 9,74 | 0,08 | 0,04 | 0,39 | 0,37 | 0,54 | 0,92 | 0,01 | 0,08 |
|  |  | SMH14 | 9,07 | 0,01 | 0,01 | 0,65 | 0,37 | 0,47 | 0,90 | 0,00 | 0,10 |
|  |  | SMH15 | 9,64 | 0,11 | 0,08 | 0,20 | 0,20 | 0,46 | 0,94 | 0,01 | 0,05 |
|  | FloM | CAU14 | 13,40 | 0,01 | 0,01 | 0,98 | 0,59 | 0,23 | 0,89 | 0,00 | 0,11 |
|  |  | AUB15 | 8,27 | 0,01 | 0,01 | 0,12 | 0,32 | 1,43 | 0,95 | 0,00 | 0,05 |
|  |  | RHO15 | 16,24 | 0,08 | 0,00 | 0,89 | 0,62 | 0,41 | 0,91 | 0,00 | 0,08 |
|  |  | MOR14 | 3,90 | 0,10 | 0,17 | 0,28 | 0,21 | 0,43 | 0,84 | 0,02 | 0,14 |
|  |  | VIL15 | 8,01 | 0,03 | 0,01 | 0,66 | 0,60 | 0,42 | 0,86 | 0,00 | 0,14 |
|  |  | SMH14 | 6,34 | 0,01 | 0,01 | 0,44 | 0,31 | 0,39 | 0,89 | 0,00 | 0,11 |
|  |  | SMH15 | 8,50 | 0,04 | 0,02 | 0,13 | 0,15 | 0,82 | 0,96 | 0,00 | 0,03 |
|  | Hum | CAU14 | 8,59 | 0,00 | 0,00 | 0,44 | 1,86 | 1,66 | 0,79 | 0,00 | 0,21 |
|  |  | AUB15 | 9,90 | 0,00 | 0,01 | 0,00 | 0,01 | 1,49 | 1,00 | 0,00 | 0,00 |
|  |  | RHO15 | 11,24 | 0,01 | 0,00 | 0,54 | 2,26 | 0,67 | 0,80 | 0,00 | 0,20 |
|  |  | MOR14 | 5,54 | 0,00 | 0,00 | 0,00 | 0,00 | 1,93 | 1,00 | 0,00 | 0,00 |
|  |  | VIL15 | 8,19 | 0,00 | 0,36 | 0,62 | 0,75 | 0,51 | 0,83 | 0,00 | 0,17 |
|  |  | SMH14 | 8,35 | 0,00 | 0,37 | 0,15 | 0,13 | 0,99 | 0,93 | 0,00 | 0,07 |
|  |  | SMH15 | 5,97 | 0,51 | 0,97 | 0,33 | 0,22 | 1,26 | 0,75 | 0,06 | 0,19 |
|  | GY | CAU14 | 200,03 | 64,73 | 0,00 | 0,12 | 0,56 | 98,92 | 0,75 | 0,24 | 0,00 |
|  |  | AUB15 | 272,10 | 5,90 | 0,05 | 8,44 | 40,04 | 139,59 | 0,83 | 0,02 | 0,15 |
|  |  | RHO15 | 192,16 | 1,64 | 0,32 | 16,24 | 12,62 | 94,51 | 0,86 | 0,01 | 0,13 |
|  |  | MOR14 | 140,42 | 2,37 | 1,02 | 11,84 | 12,96 | 86,29 | 0,83 | 0,01 | 0,15 |
|  |  | VIL15 | 106,25 | 0,00 | 0,00 | 0,01 | 0,01 | 67,03 | 1,00 | 0,00 | 0,00 |
|  |  | SMH14 | 212,97 | 0,00 | 0,00 | 8,29 | 32,08 | 93,09 | 0,84 | 0,00 | 0,16 |
|  |  | SMH15 | 111,15 | 0,00 | 0,00 | 0,31 | 17,43 | 143,53 | 0,86 | 0,00 | 0,14 |
| Het2 | FloF | JAR16 | 13,64 | 0,06 | 0,22 | 0,78 | 0,65 | 0,65 | 0,89 | 0,00 | 0,11 |
|  |  | AUB17 | 12,39 | 0,57 | 0,03 | 0,11 | 0,11 | 0,65 | 0,94 | 0,04 | 0,02 |
|  |  | SOU17 | 5,75 | 0,21 | 1,79 | 0,14 | 0,15 | 1,78 | 0,71 | 0,03 | 0,26 |
|  |  | SMH16 | 7,13 | 0,12 | 0,38 | 0,12 | 0,13 | 0,91 | 0,90 | 0,02 | 0,08 |
|  |  | SMH17 | 6,69 | 0,39 | 0,75 | 1,10 | 0,74 | 0,84 | 0,69 | 0,04 | 0,27 |
|  | FloM | JAR16 | 9,74 | 0,03 | 0,05 | 0,48 | 0,56 | 0,51 | 0,90 | 0,00 | 0,10 |
|  |  | AUB17 | 8,46 | 0,18 | 0,01 | 0,04 | 0,04 | 1,12 | 0,97 | 0,02 | 0,01 |
|  |  | SOU17 | 4,27 | 0,14 | 1,34 | 0,07 | 0,08 | 2,20 | 0,72 | 0,02 | 0,25 |
|  |  | SMH16 | 5,97 | 0,01 | 0,01 | 0,38 | 0,43 | 1,01 | 0,88 | 0,00 | 0,12 |
|  |  | SMH17 | 5,80 | 0,35 | 0,07 | 0,28 | 0,26 | 0,55 | 0,86 | 0,05 | 0,09 |
|  | Hum | JAR16 | 7,06 | 0,45 | 0,02 | 1,37 | 0,39 | 1,81 | 0,76 | 0,05 | 0,19 |
|  |  | AUB17 | 12,23 | 0,80 | 0,10 | 0,60 | 0,24 | 1,87 | 0,88 | 0,06 | 0,07 |
|  |  | SOU17 | 8,02 | 0,00 | 0,98 | 0,22 | 0,29 | 2,51 | 0,84 | 0,00 | 0,16 |
|  |  | SMH16 | 2,20 | 0,03 | 0,01 | 0,03 | 0,03 | 1,97 | 0,96 | 0,01 | 0,03 |
|  |  | SMH17 | 3,47 | 0,36 | 0,36 | 0,07 | 0,05 | 0,30 | 0,81 | 0,08 | 0,11 |
|  | GY | JAR16 | 82,07 | 4,52 | 19,39 | 2,86 | 2,75 | 48,02 | 0,74 | 0,04 | 0,22 |
|  |  | AUB17 | 167,46 | 31,85 | 18,96 | 4,88 | 3,76 | 149,76 | 0,74 | 0,14 | 0,12 |
|  |  | SOU17 | 292,57 | 40,63 | 23,47 | 16,90 | 17,91 | 158,31 | 0,75 | 0,10 | 0,15 |
|  |  | SMH16 | 75,43 | 35,07 | 0,60 | 0,76 | 0,68 | 81,76 | 0,67 | 0,31 | 0,02 |
|  |  | SMH17 | 110,50 | 35,18 | 0,01 | 0,00 | 0,00 | 37,28 | 0,76 | 0,24 | 0,00 |

The variances $\sigma_{a}^{2}$ , $\sigma_{d}^{2}$ , $\sigma_{aa}^{2}$ ,$\sigma_{ad}^{2}$ ,$\sigma_{dd}^{2}$ and $\sigma_{\varepsilon}^{2}$ are respectively for additivity, dominance, additive-by-additive epistasis, additive-by-dominance epistasis, dominance-by-dominance epistasis and error effects. The epistatic variance $\sigma_{I}^{2}$ is the sum of $\sigma_{aa}^{2}$ ,$\sigma_{ad}^{2}$ and$\sigma_{dd}^{2}$ variances. V(G) is the global genetic variance defined as: $V\left( G \right)= \sigma_{a}^{2}+\sigma_{d}^{2}+\sigma_{aa}^{2}+\sigma_{ad}^{2}+\sigma_{dd}^{2}$.

## S6 Table: Multi environment variance partition (MULTI model) in Het1

|  | **FloF** | **FloM** | **Hum** | **GY** |
| --- | --- | --- | --- | --- |
| $\boldsymbol{\sigma}_{\boldsymbol{a}}^{\boldsymbol{2}}$ | 8,56 | 7,08 | 6,45 | 112,18 |
| $\boldsymbol{\sigma}_{\boldsymbol{a}\boldsymbol{(}\boldsymbol{CAU}\boldsymbol{14}\boldsymbol{)}}^{\boldsymbol{2}}$ | 2,34 | 1,51 | 0,57 | 25,00 |
| $\boldsymbol{\sigma}_{\boldsymbol{a}\boldsymbol{(}\boldsymbol{AUB}\boldsymbol{15}\boldsymbol{)}}^{\boldsymbol{2}}$ | 0,87 | 1,27 | 0,72 | 19,70 |
| $\boldsymbol{\sigma}_{\boldsymbol{a}\boldsymbol{(}\boldsymbol{RHO}\boldsymbol{15}\boldsymbol{)}}^{\boldsymbol{2}}$ | 0,72 | 0,46 | 0,64 | 75,48 |
| $\boldsymbol{\sigma}_{\boldsymbol{a}\boldsymbol{(}\boldsymbol{MOR}\boldsymbol{14}\boldsymbol{)}}^{\boldsymbol{2}}$ | 0,44 | 0,01 | 2,62 | 94,66 |
| $\boldsymbol{\sigma}_{\boldsymbol{a}\boldsymbol{(}\boldsymbol{VIL}\boldsymbol{15}\boldsymbol{)}}^{\boldsymbol{2}}$ | 1,26 | 2,42 | 1,90 | 22,39 |
| $\boldsymbol{\sigma}_{\boldsymbol{a}\boldsymbol{(}\boldsymbol{SMH}\boldsymbol{14}\boldsymbol{)}}^{\boldsymbol{2}}$ | 0,23 | 0,20 | 1,16 | 3,54 |
| $\boldsymbol{\sigma}_{\boldsymbol{a}\boldsymbol{(}\boldsymbol{SMH}\boldsymbol{15}\boldsymbol{)}}^{\boldsymbol{2}}$ | 0,08 | 0,08 | 0,18 | 49,36 |
| $\boldsymbol{\sigma}_{\boldsymbol{d}}^{\boldsymbol{2}}$ | 0,03 | 0,02 | 0,00 | 0,00 |
| $\boldsymbol{\sigma}_{\boldsymbol{d}\boldsymbol{(}\boldsymbol{CAU}\boldsymbol{14}\boldsymbol{)}}^{\boldsymbol{2}}$ | 1,00 | 0,71 | 1,81 | 52,37 |
| $\boldsymbol{\sigma}_{\boldsymbol{d}\boldsymbol{(}\boldsymbol{AUB}\boldsymbol{15}\boldsymbol{)}}^{\boldsymbol{2}}$ | 0,07 | 0,00 | 0,00 | 23,48 |
| $\boldsymbol{\sigma}_{\boldsymbol{d}\boldsymbol{(}\boldsymbol{RHO}\boldsymbol{15}\boldsymbol{)}}^{\boldsymbol{2}}$ | 0,06 | 0,02 | 0,73 | 0,05 |
| $\boldsymbol{\sigma}_{\boldsymbol{d}\boldsymbol{(}\boldsymbol{MOR}\boldsymbol{14}\boldsymbol{)}}^{\boldsymbol{2}}$ | 0,04 | 0,00 | 0,38 | 54,13 |
| $\boldsymbol{\sigma}_{\boldsymbol{d}\boldsymbol{(}\boldsymbol{VIL}\boldsymbol{15}\boldsymbol{)}}^{\boldsymbol{2}}$ | 2,01 | 2,20 | 2,81 | 32,39 |
| $\boldsymbol{\sigma}_{\boldsymbol{d}\boldsymbol{(}\boldsymbol{SMH}\boldsymbol{14}\boldsymbol{)}}^{\boldsymbol{2}}$ | 0,57 | 0,69 | 1,23 | 2,07 |
| $\boldsymbol{\sigma}_{\boldsymbol{d}\boldsymbol{(}\boldsymbol{SMH}\boldsymbol{15}\boldsymbol{)}}^{\boldsymbol{2}}$ | 0,76 | 0,51 | 2,04 | 0,00 |
| $\boldsymbol{\sigma}_{\boldsymbol{aa}}^{\boldsymbol{2}}$ | 0,01 | 0,01 | 0,04 | 0,00 |
| $\boldsymbol{\sigma}_{\boldsymbol{ad}}^{\boldsymbol{2}}$ | 0,41 | 0,31 | 0,03 | 3,82 |
| $\boldsymbol{\sigma}_{\boldsymbol{dd}}^{\boldsymbol{2}}$ | 0,58 | 0,38 | 0,11 | 18,71 |
| $\boldsymbol{\sigma}_{\boldsymbol{\varepsilon}\boldsymbol{(}\boldsymbol{CAU}\boldsymbol{14}\boldsymbol{)}}^{\boldsymbol{2}}$ | 0,40 | 0,23 | 1,90 | 94,31 |
| $\boldsymbol{\sigma}_{\boldsymbol{\varepsilon}\boldsymbol{(}\boldsymbol{AUB}\boldsymbol{15}\boldsymbol{)}}^{\boldsymbol{2}}$ | 1,37 | 1,46 | 1,58 | 145,34 |
| $\boldsymbol{\sigma}_{\boldsymbol{\varepsilon}\boldsymbol{(}\boldsymbol{RHO}\boldsymbol{15}\boldsymbol{)}}^{\boldsymbol{2}}$ | 0,49 | 0,41 | 0,68 | 94,69 |
| $\boldsymbol{\sigma}_{\boldsymbol{\varepsilon}\boldsymbol{(}\boldsymbol{MOR}\boldsymbol{14}\boldsymbol{)}}^{\boldsymbol{2}}$ | 0,47 | 0,41 | 1,77 | 78,50 |
| $\boldsymbol{\sigma}_{\boldsymbol{\varepsilon}\boldsymbol{(}\boldsymbol{VIL}\boldsymbol{15}\boldsymbol{)}}^{\boldsymbol{2}}$ | 0,57 | 0,45 | 0,54 | 69,95 |
| $\boldsymbol{\sigma}_{\boldsymbol{\varepsilon}\boldsymbol{(}\boldsymbol{SMH}\boldsymbol{14}\boldsymbol{)}}^{\boldsymbol{2}}$ | 0,48 | 0,41 | 1,05 | 91,05 |
| $\boldsymbol{\sigma}_{\boldsymbol{\varepsilon}\boldsymbol{(}\boldsymbol{SMH}\boldsymbol{15}\boldsymbol{)}}^{\boldsymbol{2}}$ | 0,45 | 0,79 | 1,43 | 159,02 |

The variances $\sigma_{a}^{2}$ , $\sigma_{d}^{2}$ , $\sigma_{aa}^{2}$ ,$\sigma_{ad}^{2}$ ,$\sigma_{dd}^{2}$ and $\sigma_{\varepsilon}^{2}$ are respectively for additivity, dominance, additive-by-additive epistasis, additive-by-dominance epistasis, dominance-by-dominance epistasis and error effects. The variances $\sigma_{a(e)}^{2}$, $\sigma_{d(e)}^{2}$ and $\sigma_{\varepsilon(e)}^{2}$ are environment specific variance for the environment *e*.

## S7 Table: Multi environment variance partition (MULTI model) in Het2

|  | **FloF** | **FloM** | **Hum** | **GY** |
| --- | --- | --- | --- | --- |
| $\boldsymbol{\sigma}_{\boldsymbol{a}}^{\boldsymbol{2}}$ | 7,24 | 5,68 | 3,21 | 85,63 |
| $\boldsymbol{\sigma}_{\boldsymbol{a}\boldsymbol{(}\boldsymbol{JAR}\boldsymbol{16}\boldsymbol{)}}^{\boldsymbol{2}}$ | 2,02 | 0,95 | 4,75 | 45,70 |
| $\boldsymbol{\sigma}_{\boldsymbol{a}\boldsymbol{(}\boldsymbol{AUB}\boldsymbol{17}\boldsymbol{)}}^{\boldsymbol{2}}$ | 0,60 | 0,03 | 2,83 | 116,35 |
| $\boldsymbol{\sigma}_{\boldsymbol{a}\boldsymbol{(}\boldsymbol{SOU}\boldsymbol{17}\boldsymbol{)}}^{\boldsymbol{2}}$ | 0,25 | 0,16 | 0,01 | 0,26 |
| $\boldsymbol{\sigma}_{\boldsymbol{a}\boldsymbol{(}\boldsymbol{SMH}\boldsymbol{16}\boldsymbol{)}}^{\boldsymbol{2}}$ | 0,07 | 0,39 | 0,01 | 22,67 |
| $\boldsymbol{\sigma}_{\boldsymbol{a}\boldsymbol{(}\boldsymbol{SMH}\boldsymbol{17}\boldsymbol{)}}^{\boldsymbol{2}}$ | 2,59 | 1,95 | 2,77 | 69,70 |
| $\boldsymbol{\sigma}_{\boldsymbol{d}}^{\boldsymbol{2}}$ | 0,23 | 0,15 | 0,03 | 11,25 |
| $\boldsymbol{\sigma}_{\boldsymbol{d}\boldsymbol{(}\boldsymbol{JAR}\boldsymbol{16}\boldsymbol{)}}^{\boldsymbol{2}}$ | 0,29 | 0,00 | 1,32 | 37,72 |
| $\boldsymbol{\sigma}_{\boldsymbol{d}\boldsymbol{(}\boldsymbol{AUB}\boldsymbol{17}\boldsymbol{)}}^{\boldsymbol{2}}$ | 0,07 | 0,24 | 0,48 | 70,29 |
| $\boldsymbol{\sigma}_{\boldsymbol{d}\boldsymbol{(}\boldsymbol{SOU}\boldsymbol{17}\boldsymbol{)}}^{\boldsymbol{2}}$ | 0,30 | 0,26 | 0,00 | 34,62 |
| $\boldsymbol{\sigma}_{\boldsymbol{d}\boldsymbol{(}\boldsymbol{SMH}\boldsymbol{16}\boldsymbol{)}}^{\boldsymbol{2}}$ | 1,37 | 0,47 | 3,11 | 23,08 |
| $\boldsymbol{\sigma}_{\boldsymbol{d}\boldsymbol{(}\boldsymbol{SMH}\boldsymbol{17}\boldsymbol{)}}^{\boldsymbol{2}}$ | 1,03 | 0,58 | 1,66 | 6,33 |
| $\boldsymbol{\sigma}_{\boldsymbol{aa}}^{\boldsymbol{2}}$ | 0,62 | 0,01 | 0,14 | 0,35 |
| $\boldsymbol{\sigma}_{\boldsymbol{ad}}^{\boldsymbol{2}}$ | 0,10 | 0,19 | 0,13 | 0,14 |
| $\boldsymbol{\sigma}_{\boldsymbol{dd}}^{\boldsymbol{2}}$ | 0,08 | 0,21 | 0,17 | 0,08 |
| $\boldsymbol{\sigma}_{\boldsymbol{\varepsilon}\boldsymbol{(}\boldsymbol{JAR}\boldsymbol{16}\boldsymbol{)}}^{\boldsymbol{2}}$ | 0,67 | 0,52 | 1,83 | 48,26 |
| $\boldsymbol{\sigma}_{\boldsymbol{\varepsilon}\boldsymbol{(}\boldsymbol{AUB}\boldsymbol{17}\boldsymbol{)}}^{\boldsymbol{2}}$ | 0,66 | 1,04 | 1,89 | 154,47 |
| $\boldsymbol{\sigma}_{\boldsymbol{\varepsilon}\boldsymbol{(}\boldsymbol{SOU}\boldsymbol{17}\boldsymbol{)}}^{\boldsymbol{2}}$ | 2,17 | 2,45 | 2,86 | 162,30 |
| $\boldsymbol{\sigma}_{\boldsymbol{\varepsilon}\boldsymbol{(}\boldsymbol{SMH}\boldsymbol{16}\boldsymbol{)}}^{\boldsymbol{2}}$ | 1,01 | 1,10 | 1,71 | 81,10 |
| $\boldsymbol{\sigma}_{\boldsymbol{\varepsilon}\boldsymbol{(}\boldsymbol{SMH}\boldsymbol{17}\boldsymbol{)}}^{\boldsymbol{2}}$ | 0,93 | 0,60 | 0,30 | 38,25 |

The variances $\sigma_{a}^{2}$ , $\sigma_{d}^{2}$ , $\sigma_{aa}^{2}$ ,$\sigma_{ad}^{2}$ ,$\sigma_{dd}^{2}$ and $\sigma_{\varepsilon}^{2}$ are respectively for additivity, dominance, additive-by-additive epistasis, additive-by-dominance epistasis, dominance-by-dominance epistasis and error effects. The variances $\sigma_{a(e)}^{2}$, $\sigma_{d(e)}^{2}$ and $\sigma_{\varepsilon(e)}^{2}$ are environment specific variance for the environment *e*.

## S8 Table: Synthetic table of multiple environment variance partition (MULTI model)

| **Panel** | **Trait** | $\boldsymbol{\sigma}_{\boldsymbol{a}}^{\boldsymbol{2}}$ | $\boldsymbol{V(A\times E)}$ | $\boldsymbol{\sigma}_{\boldsymbol{d}}^{\boldsymbol{2}}$ | $\boldsymbol{V(D\times E)}$ | $\boldsymbol{\sigma}_{\boldsymbol{aa}}^{\boldsymbol{2}}$ | $\boldsymbol{\sigma}_{\boldsymbol{ad}}^{\boldsymbol{2}}$ | $\boldsymbol{\sigma}_{\boldsymbol{dd}}^{\boldsymbol{2}}$ | $\boldsymbol{V(}\boldsymbol{\varepsilon\times}\boldsymbol{E)}$ | $\frac{\boldsymbol{\sigma}_{\boldsymbol{a}}^{\boldsymbol{2}}}{\boldsymbol{V}\boldsymbol{(}\boldsymbol{G}\boldsymbol{)}}$ | $\frac{\boldsymbol{\sigma}_{\boldsymbol{d}}^{\boldsymbol{2}}}{\boldsymbol{V}\boldsymbol{(}\boldsymbol{G}\boldsymbol{)}}$ | $\frac{\boldsymbol{V}\boldsymbol{(}\boldsymbol{A}\boldsymbol{\times}\boldsymbol{E}\boldsymbol{)}}{\boldsymbol{V}\boldsymbol{(}\boldsymbol{G}\boldsymbol{)}}$ | $\frac{\boldsymbol{V}\boldsymbol{(}\boldsymbol{D}\boldsymbol{\times}\boldsymbol{E}\boldsymbol{)}}{\boldsymbol{V}\boldsymbol{(}\boldsymbol{G}\boldsymbol{)}}$ |
| --- | --- | --- | --- | --- | --- | --- | --- | --- | --- | --- | --- | --- | --- |
| Het1 | FloF | 8,56 | 0,85 | 0,03 | 0,64 | 0,01 | 0,41 | 0,58 | 0,60 | 0,89 | 0,00 | 0,09 | 0,07 |
|  | FloM | 7,08 | 0,85 | 0,02 | 0,59 | 0,01 | 0,31 | 0,38 | 0,59 | 0,91 | 0,00 | 0,11 | 0,08 |
|  | Hum | 6,45 | 1,11 | 0,00 | 1,28 | 0,04 | 0,03 | 0,11 | 1,28 | 0,97 | 0,00 | 0,17 | 0,19 |
|  | GY | 112,18 | 41,44 | 0,00 | 23,50 | 0,00 | 3,82 | 18,71 | 104,69 | 0,83 | 0,00 | 0,31 | 0,17 |
| Het2 | FloF | 7,24 | 1,10 | 0,23 | 0,61 | 0,62 | 0,10 | 0,08 | 1,09 | 0,88 | 0,03 | 0,13 | 0,07 |
|  | FloM | 5,68 | 0,70 | 0,15 | 0,31 | 0,01 | 0,19 | 0,21 | 1,14 | 0,91 | 0,02 | 0,11 | 0,05 |
|  | Hum | 3,21 | 2,07 | 0,03 | 1,31 | 0,14 | 0,13 | 0,17 | 1,72 | 0,87 | 0,01 | 0,56 | 0,36 |
|  | GY | 85,63 | 50,93 | 11,25 | 34,41 | 0,35 | 0,14 | 0,08 | 96,88 | 0,88 | 0,12 | 0,52 | 0,35 |

The variances $\sigma_{a}^{2}$ , $\sigma_{d}^{2}$ , $\sigma_{aa}^{2}$ ,$\sigma_{ad}^{2}$ ,$\sigma_{dd}^{2}$ and $\sigma_{\varepsilon}^{2}$ are respectively for additivity, dominance, additive-by-additive epistasis, additive-by-dominance epistasis, dominance-by-dominance epistasis and error effects. The variances $\sigma_{a(e)}^{2}$, $\sigma_{d(e)}^{2}$ and $\sigma_{\varepsilon(e)}^{2}$ are environment specific variance for the environment *e*. V(AxE) is the average additive-by-environment interaction variance (calculated as $\frac{\sum_{\boldsymbol{e}} \boldsymbol{\sigma}_{\boldsymbol{a}\boldsymbol{(e)}}^{\boldsymbol{2}}}{\boldsymbol{n}_{\boldsymbol{e}}}$). V(DxE) is the average dominance-by-environment interaction variance (calculated as $\frac{\sum_{e} \sigma_{d(e)}^{2}}{n_{e}}$). $V(\varepsilon\times E)$ is the average environment specific error variance (calculated as $\frac{\sum_{\boldsymbol{e}} \boldsymbol{\sigma}_{\boldsymbol{d}\boldsymbol{(e)}}^{\boldsymbol{2}}}{\boldsymbol{n}_{\boldsymbol{e}}}$). The term $n_{e}$ refers to the number of environments. V(G) is the main genetic variance of main effects defined as: $V\left( G \right)= \sigma_{a}^{2}+\sigma_{d}^{2}+\sigma_{aa}^{2}+\sigma_{ad}^{2}+\sigma_{dd}^{2}$.

## S9 Table. Number of candidate markers in Het1 for model Gad.

| **Trait** | **Env** | $\boldsymbol{\alpha}$ | $\boldsymbol{\delta}$ |
| --- | --- | --- | --- |
| FloF | AUB15 | 345860 | 345860 |
|  | MOR14 | 348579 | 348579 |
|  | VIL15 | 329092 | 329092 |
|  | SMH14 | 347024 | 347024 |
|  | SMH15 | 349883 | 349883 |
|  | CAU14 | 351133 | 351133 |
|  | CON15 | 346542 | 346542 |
|  | MULTI | 433854 | 433854 |
| FloM | AUB15 | 345860 | 345860 |
|  | MOR14 | 349307 | 349307 |
|  | VIL15 | 329092 | 329092 |
|  | SMH14 | 347024 | 347024 |
|  | SMH15 | 350601 | 350601 |
|  | CAU14 | 351847 | 351847 |
|  | CON15 | 346542 | 346542 |
|  | MULTI | 433854 | 433854 |
| Hum | AUB15 | 346337 | 346337 |
|  | MOR14 | 349307 | 349307 |
|  | VIL15 | 329092 | 329092 |
|  | SMH14 | 347639 | 347639 |
|  | SMH15 | 350601 | 350601 |
|  | CAU14 | 351847 | 351847 |
|  | CON15 | 346542 | 346542 |
|  | MULTI | 433986 | 433986 |
| GY | AUB15 | 346337 | 346337 |
|  | MOR14 | 349307 | 349307 |
|  | VIL15 | 329092 | 329092 |
|  | SMH14 | 347639 | 347639 |
|  | SMH15 | 350601 | 350601 |
|  | CAU14 | 351847 | 351847 |
|  | CON15 | 346542 | 346542 |
|  | MULTI | 433986 | 433986 |

Number of candidate markers according to the environments, the traits and the contrast tests after filter on the minor allele frequency (MAF) and the number of individuals in each genotypic class. AUB14, MOR14, VIL15, SMH14, SMH15, CAU14 and CON15 indicate for Gso-MONO models. MULTI indicates for the Gso-MULTI model.

## S10 Table. Number of candidate markers in Het2 for model Gad.

| **Trait** | **Env** | $\boldsymbol{\alpha}$ | $\boldsymbol{\delta}$ |
| --- | --- | --- | --- |
| FloF | JAR16 | 365725 | 365725 |
|  | SOU17 | 377282 | 377282 |
|  | AUB17 | 370975 | 370975 |
|  | SMH16 | 372279 | 372279 |
|  | SMH17 | 378696 | 378696 |
|  | MULTI | 451578 | 451578 |
| FloM | JAR16 | 366009 | 366009 |
|  | SOU17 | 377282 | 377282 |
|  | AUB17 | 370975 | 370975 |
|  | SMH16 | 372279 | 372279 |
|  | SMH17 | 378696 | 378696 |
|  | MULTI | 451361 | 451361 |
| Hum | JAR16 | 365364 | 365364 |
|  | SOU17 | 377282 | 377282 |
|  | AUB17 | 370975 | 370975 |
|  | SMH16 | 373073 | 373073 |
|  | SMH17 | 378696 | 378696 |
|  | MULTI | 450583 | 450583 |
| GY | JAR16 | 343449 | 343449 |
|  | SOU17 | 377282 | 377282 |
|  | AUB17 | 370975 | 370975 |
|  | SMH16 | 373073 | 373073 |
|  | SMH17 | 378696 | 378696 |
|  | MULTI | 449104 | 449104 |

Number of candidate markers according to the environments, the traits and the contrast tests after filter on the minor allele frequency (MAF) and the number of individuals in each genotypic class. JAR16, AUB17, SOU17, SMH16 and SMH17 indicate for Gso-MONO models. MULTI indicates for the Gso-MULTI model.

## S11 Table. Number of candidate markers in Het1 for model Gso.

| **Trait** | **Env** | $\boldsymbol{a}_{\boldsymbol{DF}}$ | $\boldsymbol{d}_{\boldsymbol{DF}}$ | $\boldsymbol{\Delta}_{\boldsymbol{LD}}$ | $\boldsymbol{g}_{\boldsymbol{Het}\boldsymbol{1}}$ |
| --- | --- | --- | --- | --- | --- |
| FloF | AUB15 | 345860 | 344253 | 428623 | 344253 |
|  | MOR14 | 348579 | 346223 | 429835 | 346223 |
|  | VIL15 | 327046 | 324343 | 416506 | 324343 |
|  | SMH14 | 347024 | 345455 | 430084 | 345455 |
|  | SMH15 | 349883 | 348330 | 431656 | 348330 |
|  | CAU14 | 351133 | 349213 | 431250 | 349213 |
|  | CON15 | 346542 | 344630 | 429036 | 344630 |
|  | MULTI | 433854 | 432675 | 471123 | 432675 |
| FloM | AUB15 | 345860 | 344253 | 428623 | 344253 |
|  | MOR14 | 349307 | 346951 | 430743 | 346951 |
|  | VIL15 | 329092 | 326376 | 419050 | 326376 |
|  | SMH14 | 347024 | 345455 | 430084 | 345455 |
|  | SMH15 | 350601 | 349046 | 432530 | 349046 |
|  | CAU14 | 351847 | 349927 | 432142 | 349927 |
|  | CON15 | 346542 | 344630 | 429036 | 344630 |
|  | MULTI | 433854 | 432675 | 471123 | 432675 |
| Hum | AUB15 | 346337 | 344484 | 428809 | 344484 |
|  | MOR14 | 349307 | 346951 | 430743 | 346951 |
|  | VIL15 | 329092 | 326376 | 419050 | 326376 |
|  | SMH14 | 347639 | 346090 | 430381 | 346090 |
|  | SMH15 | 350601 | 349046 | 432530 | 349046 |
|  | CAU14 | 351847 | 349927 | 432142 | 349927 |
|  | CON15 | 346542 | 344630 | 429036 | 344630 |
|  | MULTI | 433986 | 432808 | 471147 | 432808 |
| GY | AUB15 | 346337 | 344484 | 428809 | 344484 |
|  | MOR14 | 349307 | 346951 | 430743 | 346951 |
|  | VIL15 | 329092 | 326376 | 419050 | 326376 |
|  | SMH14 | 347639 | 346090 | 430381 | 346090 |
|  | SMH15 | 350601 | 349046 | 432530 | 349046 |
|  | CAU14 | 351847 | 349927 | 432142 | 349927 |
|  | CON15 | 346542 | 344630 | 429036 | 344630 |
|  | MULTI | 433986 | 432808 | 471147 | 432808 |

Number of candidate markers according to the environments, the traits and the contrast tests after filter on the minor allele frequency (MAF) and the number of individuals in each genotypic class. AUB14, MOR14, VIL15, SMH14, SMH15, CAU14 and CON15 indicate for Gso-MONO models. MULTI indicates for the Gso-MULTI model.

## S12 Table: Number of candidate markers in Het2 for model Gso.

| **Trait** | **Env** | $\boldsymbol{a}$ | $\boldsymbol{a}_{\boldsymbol{DD}}$ | $\boldsymbol{a}_{\boldsymbol{DF}}$ | $\boldsymbol{a}_{\boldsymbol{FF}}$ | $\boldsymbol{s}_{\boldsymbol{a}}$ | $\boldsymbol{t}_{\boldsymbol{a}}$ | $\boldsymbol{d}$ | $\boldsymbol{d}_{\boldsymbol{DD}}$ | $\boldsymbol{d}_{\boldsymbol{DF}}$ | $\boldsymbol{d}_{\boldsymbol{FF}}$ | $\boldsymbol{s}_{\boldsymbol{d}}$ | $\boldsymbol{t}_{\boldsymbol{d}}$ | $\boldsymbol{\Delta}_{\boldsymbol{LD}}$ | $\boldsymbol{o}_{\boldsymbol{a}}$ | $\boldsymbol{o}_{\boldsymbol{d}}$ | $\boldsymbol{g}_{\boldsymbol{Het}\boldsymbol{2}}$ | $\boldsymbol{g}_{\boldsymbol{Het}\boldsymbol{1}}$ |
| --- | --- | --- | --- | --- | --- | --- | --- | --- | --- | --- | --- | --- | --- | --- | --- | --- | --- | --- |
| FloF | JAR16 | 91622 | 202061 | 260774 | 183863 | 92357 | 91622 | 91396 | 202060 | 248892 | 183862 | 92355 | 91396 | 356715 | 92357 | 91622 | 91396 | 248892 |
|  | SOU17 | 106529 | 219019 | 278583 | 199993 | 107238 | 106529 | 106353 | 219019 | 268850 | 199986 | 107238 | 106353 | 370451 | 107238 | 106529 | 106353 | 268850 |
|  | AUB17 | 66935 | 148659 | 191596 | 133191 | 67418 | 66935 | 66789 | 148658 | 182739 | 133191 | 67417 | 66789 | 257959 | 67418 | 66935 | 66789 | 182739 |
|  | SMH16 | 99561 | 216040 | 265965 | 191216 | 100388 | 99561 | 99377 | 216040 | 255006 | 191216 | 100388 | 99377 | 360979 | 100388 | 99561 | 99377 | 255006 |
|  | SMH17 | 108462 | 220663 | 278234 | 203149 | 109112 | 108462 | 108321 | 220663 | 268827 | 203148 | 109111 | 108321 | 373471 | 109112 | 108462 | 108321 | 268827 |
|  | MULTI | 257011 | 353402 | 394626 | 344940 | 260251 | 257011 | 256734 | 353385 | 390239 | 344857 | 260160 | 256734 | 446459 | 260251 | 257011 | 256734 | 390239 |
| FloM | JAR16 | 92097 | 202367 | 261091 | 184348 | 92818 | 92097 | 91870 | 202366 | 249249 | 184347 | 92816 | 91870 | 357049 | 92818 | 92097 | 91870 | 249249 |
|  | SOU17 | 106529 | 219019 | 278583 | 199993 | 107238 | 106529 | 106353 | 219019 | 268850 | 199986 | 107238 | 106353 | 370451 | 107238 | 106529 | 106353 | 268850 |
|  | AUB17 | 94496 | 205538 | 269028 | 190784 | 95162 | 94496 | 94329 | 205537 | 257333 | 190784 | 95161 | 94329 | 360220 | 95162 | 94496 | 94329 | 257333 |
|  | SMH16 | 99561 | 216040 | 265965 | 191216 | 100388 | 99561 | 99377 | 216040 | 255006 | 191216 | 100388 | 99377 | 360979 | 100388 | 99561 | 99377 | 255006 |
|  | SMH17 | 108462 | 220663 | 278234 | 203149 | 109112 | 108462 | 108321 | 220663 | 268827 | 203148 | 109111 | 108321 | 373471 | 109112 | 108462 | 108321 | 268827 |
|  | MULTI | 257319 | 353607 | 394718 | 345141 | 260546 | 257319 | 257041 | 353593 | 390329 | 345058 | 260455 | 257041 | 446478 | 260546 | 257319 | 257041 | 390329 |
| Hum | JAR16 | 90999 | 201111 | 260259 | 183333 | 91681 | 90999 | 90772 | 201110 | 247924 | 183332 | 91679 | 90772 | 355677 | 91681 | 90999 | 90772 | 247924 |
|  | SOU17 | 106529 | 219019 | 278583 | 199993 | 107238 | 106529 | 106353 | 219019 | 268850 | 199986 | 107238 | 106353 | 370451 | 107238 | 106529 | 106353 | 268850 |
|  | AUB17 | 94496 | 205538 | 269028 | 190784 | 95162 | 94496 | 94329 | 205537 | 257333 | 190784 | 95161 | 94329 | 360220 | 95162 | 94496 | 94329 | 257333 |
|  | SMH16 | 101365 | 217806 | 266829 | 192799 | 102195 | 101365 | 101172 | 217806 | 256279 | 192799 | 102195 | 101172 | 362605 | 102195 | 101365 | 101172 | 256279 |
|  | SMH17 | 108462 | 220663 | 278234 | 203149 | 109112 | 108462 | 108321 | 220663 | 268827 | 203148 | 109111 | 108321 | 373471 | 109112 | 108462 | 108321 | 268827 |
|  | MULTI | 257171 | 353481 | 394423 | 345048 | 260409 | 257171 | 256893 | 353457 | 390034 | 344965 | 260320 | 256893 | 446418 | 260409 | 257171 | 256893 | 390034 |
| GY | JAR16 | 63294 | 166041 | 234267 | 150303 | 64066 | 63294 | 62955 | 166040 | 216824 | 150302 | 64065 | 62955 | 328388 | 64066 | 63294 | 62955 | 216824 |
|  | SOU17 | 106529 | 219019 | 278583 | 199993 | 107238 | 106529 | 106353 | 219019 | 268850 | 199986 | 107238 | 106353 | 370451 | 107238 | 106529 | 106353 | 268850 |
|  | AUB17 | 94496 | 205538 | 269028 | 190784 | 95162 | 94496 | 94329 | 205537 | 257333 | 190784 | 95161 | 94329 | 360220 | 95162 | 94496 | 94329 | 257333 |
|  | SMH16 | 101365 | 217806 | 266829 | 192799 | 102195 | 101365 | 101172 | 217806 | 256279 | 192799 | 102195 | 101172 | 362605 | 102195 | 101365 | 101172 | 256279 |
|  | SMH17 | 108462 | 220663 | 278234 | 203149 | 109112 | 108462 | 108321 | 220663 | 268827 | 203148 | 109111 | 108321 | 373471 | 109112 | 108462 | 108321 | 268827 |
|  | MULTI | 249305 | 346812 | 390188 | 340013 | 252539 | 249305 | 249062 | 346776 | 385529 | 339926 | 252441 | 249062 | 444713 | 252539 | 249305 | 249062 | 385529 |

Number of candidate markers according to the environments, the traits and the contrast tests after filter on the minor allele frequency (MAF) and the number of individuals in each genotypic class. JAR16, AUB17, SOU17, SMH16 and SMH17 indicate for Gso-MONO models. MULTI indicates for the Gso-MULTI model.

## S13 Table: Number of significant markers in Het1

| **Trait** | **Env** | $\boldsymbol{a}_{\boldsymbol{DF}}$ | $\boldsymbol{d}_{\boldsymbol{DF}}$ | $\boldsymbol{\Delta}_{\boldsymbol{LD}}$ | $\boldsymbol{g}_{\boldsymbol{Het}\boldsymbol{1}}$ |
| --- | --- | --- | --- | --- | --- |
| FloF | AUB15 | 54 \| 0 | 5 \| 0 | 0 \| 0 | 25 \| 0 |
|  | MOR14 | 41 \| 4 | 2 \| 0 | 0 \| 0 | 9 \| 4 |
|  | VIL15 | 27 \| 1 | 21 \| 0 | 0 \| 0 | 11 \| 0 |
|  | SMH14 | 48 \| 0 | 2 \| 0 | 0 \| 0 | 33 \| 0 |
|  | SMH15 | 20 \| 0 | 1 \| 0 | 0 \| 0 | 11 \| 0 |
|  | CAU14 | 48 \| 3 | 13 \| 0 | 0 \| 0 | 31 \| 4 |
|  | CON15 | 51 \| 1 | 5 \| 1 | 0 \| 0 | 13 \| 1 |
|  | MULTI | 68 \| 4 | 0 \| 0 | 0 \| 0 | 16 \| 4 |
| FloM | AUB15 | 10 \| 2 | 0 \| 0 | 0 \| 0 | 4 \| 0 |
|  | MOR14 | 39 \| 7 | 3 \| 0 | 4 \| 0 | 24 \| 2 |
|  | VIL15 | 5 \| 1 | 0 \| 0 | 1 \| 0 | 1 \| 0 |
|  | SMH14 | 14 \| 1 | 1 \| 0 | 1 \| 0 | 7 \| 0 |
|  | SMH15 | 11 \| 3 | 0 \| 0 | 1 \| 0 | 5 \| 0 |
|  | CAU14 | 10 \| 3 | 13 \| 0 | 0 \| 0 | 10 \| 1 |
|  | CON15 | 20 \| 5 | 0 \| 0 | 2 \| 0 | 11 \| 0 |
|  | MULTI | 13 \| 7 | 0 \| 0 | 0 \| 0 | 4 \| 1 |
| Hum | AUB15 | 6 \| 1 | 0 \| 0 | 0 \| 0 | 4 \| 0 |
|  | MOR14 | 5 \| 1 | 0 \| 0 | 0 \| 0 | 2 \| 0 |
|  | VIL15 | 17 \| 3 | 1 \| 0 | 0 \| 0 | 23 \| 0 |
|  | SMH14 | 27 \| 11 | 3 \| 0 | 0 \| 0 | 19 \| 0 |
|  | SMH15 | 53 \| 19 | 6 \| 1 | 1 \| 0 | 22 \| 3 |
|  | CAU14 | 3 \| 0 | 0 \| 0 | 0 \| 0 | 15 \| 0 |
|  | CON15 | 22 \| 0 | 0 \| 0 | 0 \| 0 | 2 \| 0 |
|  | MULTI | 0 \| 0 | 0 \| 0 | 0 \| 0 | 18 \| 0 |
| GY | AUB15 | 21 \| 1 | 12 \| 0 | 12 \| 1 | 16 \| 0 |
|  | MOR14 | 22 \| 2 | 0 \| 0 | 0 \| 0 | 7 \| 0 |
|  | VIL15 | 9 \| 5 | 3 \| 0 | 6 \| 0 | 11 \| 0 |
|  | SMH14 | 2 \| 0 | 13 \| 0 | 0 \| 0 | 8 \| 0 |
|  | SMH15 | 3 \| 1 | 2 \| 0 | 0 \| 0 | 5 \| 0 |
|  | CAU14 | 1 \| 0 | 0 \| 0 | 0 \| 0 | 0 \| 0 |
|  | CON15 | 3 \| 0 | 1 \| 0 | 1 \| 0 | 1 \| 0 |
|  | MULTI | 0 \| 0 | 0 \| 0 | 0 \| 0 | 0 \| 0 |

Number of significant markers identified for each trait and each environment in Het1. The first and second value are respectively for an FDR levels of 0.2 and 0.05. CAU14, AUB14, CON15, MOR14, VIL15, SMH14 and SMH15 indicate for Gso-MONO models. MULTI indicates for Gso-MULTI model.

| **Trait** | **Env** | $\boldsymbol{a}$ | $\boldsymbol{a}_{\boldsymbol{DD}}$ | $\boldsymbol{a}_{\boldsymbol{DF}}$ | $\boldsymbol{a}_{\boldsymbol{FF}}$ | $\boldsymbol{s}_{\boldsymbol{a}}$ | $\boldsymbol{t}_{\boldsymbol{a}}$ | $\boldsymbol{d}$ | $\boldsymbol{d}_{\boldsymbol{DD}}$ | $\boldsymbol{d}_{\boldsymbol{DF}}$ | $\boldsymbol{d}_{\boldsymbol{FF}}$ | $\boldsymbol{s}_{\boldsymbol{d}}$ | $\boldsymbol{t}_{\boldsymbol{d}}$ | $\boldsymbol{\Delta}_{\boldsymbol{LD}}$ | $\boldsymbol{o}_{\boldsymbol{a}}$ | $\boldsymbol{o}_{\boldsymbol{d}}$ | $\boldsymbol{g}_{\boldsymbol{Het}\boldsymbol{2}}$ | $\boldsymbol{g}_{\boldsymbol{Het}\boldsymbol{1}}$ |
| --- | --- | --- | --- | --- | --- | --- | --- | --- | --- | --- | --- | --- | --- | --- | --- | --- | --- | --- |
| FloF | JAR16 | 21 \| 6 | 8 \| 0 | 217 \| 0 | 25 \| 0 | 0 \| 0 | 0 \| 0 | 2 \| 0 | 14 \| 0 | 1 \| 0 | 1 \| 0 | 0 \| 0 | 2 \| 0 | 5 \| 0 | 1275 \| 0 | 9 \| 1 | 652 \| 89 | 271 \| 0 |
|  | AUB17 | 31 \| 2 | 6 \| 1 | 252 \| 0 | 24 \| 0 | 0 \| 0 | 1 \| 0 | 1 \| 0 | 82 \| 0 | 28 \| 1 | 2 \| 0 | 0 \| 0 | 0 \| 0 | 0 \| 0 | 161 \| 0 | 4 \| 0 | 207 \| 3 | 395 \| 1 |
|  | SOU17 | 5 \| 0 | 18 \| 10 | 64 \| 0 | 26 \| 0 | 0 \| 0 | 0 \| 0 | 11 \| 0 | 30 \| 0 | 14 \| 0 | 8 \| 2 | 0 \| 0 | 0 \| 0 | 0 \| 0 | 705 \| 0 | 18 \| 7 | 366 \| 28 | 83 \| 0 |
|  | SMH16 | 9 \| 0 | 5 \| 3 | 172 \| 0 | 4 \| 0 | 0 \| 0 | 5 \| 0 | 1 \| 0 | 34 \| 0 | 5 \| 0 | 2 \| 0 | 0 \| 0 | 12 \| 0 | 1 \| 0 | 557 \| 0 | 0 \| 0 | 332 \| 42 | 206 \| 0 |
|  | SMH17 | 13 \| 0 | 65 \| 0 | 132 \| 0 | 8 \| 0 | 0 \| 0 | 2 \| 0 | 3 \| 0 | 130 \| 0 | 14 \| 1 | 21 \| 6 | 0 \| 0 | 0 \| 0 | 1 \| 0 | 647 \| 0 | 5 \| 1 | 490 \| 22 | 215 \| 0 |
|  | MULTI | 6 \| 4 | 0 \| 0 | 373 \| 52 | 0 \| 0 | 2 \| 0 | 38 \| 0 | 0 \| 0 | 30 \| 0 | 225 \| 122 | 1 \| 0 | 0 \| 0 | 0 \| 0 | 375 \| 129 | 13 \| 0 | 61 \| 0 | 132 \| 0 | 439 \| 178 |
| FloM | JAR16 | 28 \| 16 | 41 \| 0 | 51 \| 26 | 10 \| 0 | 0 \| 0 | 0 \| 0 | 0 \| 0 | 0 \| 0 | 9 \| 0 | 0 \| 0 | 0 \| 0 | 9 \| 1 | 7 \| 0 | 3602 \| 1406 | 0 \| 0 | 2449 \| 702 | 56 \| 1 |
|  | AUB17 | 35 \| 8 | 80 \| 1 | 14 \| 3 | 33 \| 0 | 0 \| 0 | 0 \| 0 | 7 \| 7 | 1 \| 0 | 67 \| 0 | 0 \| 0 | 0 \| 0 | 88 \| 71 | 2 \| 1 | 828 \| 43 | 0 \| 0 | 561 \| 43 | 17 \| 1 |
|  | SOU17 | 12 \| 1 | 25 \| 0 | 16 \| 3 | 64 \| 14 | 0 \| 0 | 0 \| 0 | 0 \| 0 | 0 \| 0 | 11 \| 0 | 2 \| 2 | 0 \| 0 | 1 \| 0 | 1 \| 0 | 1204 \| 172 | 0 \| 0 | 376 \| 26 | 25 \| 0 |
|  | SMH16 | 6 \| 0 | 11 \| 0 | 10 \| 0 | 11 \| 1 | 0 \| 0 | 3 \| 0 | 1 \| 1 | 4 \| 0 | 6 \| 0 | 0 \| 0 | 0 \| 0 | 15 \| 14 | 1 \| 0 | 1551 \| 233 | 0 \| 0 | 585 \| 63 | 5 \| 0 |
|  | SMH17 | 8 \| 0 | 2 \| 0 | 23 \| 1 | 5 \| 0 | 0 \| 0 | 0 \| 0 | 0 \| 0 | 2 \| 0 | 0 \| 0 | 0 \| 0 | 0 \| 0 | 13 \| 11 | 12 \| 0 | 720 \| 67 | 0 \| 0 | 299 \| 37 | 13 \| 0 |
|  | MULTI | 15 \| 6 | 0 \| 0 | 168 \| 0 | 0 \| 0 | 29 \| 0 | 0 \| 0 | 0 \| 0 | 9 \| 0 | 122 \| 3 | 0 \| 0 | 0 \| 0 | 0 \| 0 | 145 \| 0 | 2807 \| 245 | 0 \| 0 | 1694 \| 244 | 23 \| 4 |
| Hum | JAR16 | 49 \| 9 | 221 \| 0 | 3 \| 0 | 93 \| 1 | 0 \| 0 | 0 \| 0 | 8 \| 0 | 1 \| 0 | 0 \| 0 | 25 \| 1 | 0 \| 0 | 0 \| 0 | 0 \| 0 | 0 \| 0 | 0 \| 0 | 5 \| 0 | 0 \| 0 |
|  | AUB17 | 38 \| 0 | 98 \| 0 | 4 \| 0 | 265 \| 0 | 0 \| 0 | 0 \| 0 | 2 \| 0 | 29 \| 14 | 0 \| 0 | 2 \| 0 | 0 \| 0 | 0 \| 0 | 1 \| 0 | 0 \| 0 | 0 \| 0 | 4 \| 0 | 2 \| 2 |
|  | SOU17 | 102 \| 2 | 203 \| 5 | 6 \| 1 | 721 \| 107 | 0 \| 0 | 0 \| 0 | 1 \| 0 | 2 \| 0 | 0 \| 0 | 2 \| 0 | 0 \| 0 | 0 \| 0 | 0 \| 0 | 0 \| 0 | 0 \| 0 | 7 \| 0 | 0 \| 0 |
|  | SMH16 | 13 \| 1 | 34 \| 0 | 48 \| 3 | 273 \| 56 | 0 \| 0 | 0 \| 0 | 1 \| 0 | 1 \| 0 | 0 \| 0 | 6 \| 0 | 0 \| 0 | 0 \| 0 | 0 \| 0 | 0 \| 0 | 0 \| 0 | 9 \| 0 | 3 \| 3 |
|  | SMH17 | 44 \| 0 | 50 \| 0 | 16 \| 0 | 137 \| 5 | 0 \| 0 | 0 \| 0 | 22 \| 0 | 0 \| 0 | 0 \| 0 | 72 \| 3 | 0 \| 0 | 0 \| 0 | 2 \| 1 | 0 \| 0 | 0 \| 0 | 12 \| 1 | 0 \| 0 |
|  | MULTI | 8 \| 0 | 0 \| 0 | 0 \| 0 | 49 \| 5 | 0 \| 0 | 0 \| 0 | 0 \| 0 | 8 \| 0 | 0 \| 0 | 0 \| 0 | 0 \| 0 | 0 \| 0 | 4 \| 0 | 0 \| 0 | 0 \| 0 | 4 \| 0 | 0 \| 0 |
| GY | JAR16 | 2 \| 0 | 0 \| 0 | 0 \| 0 | 0 \| 0 | 0 \| 0 | 0 \| 0 | 0 \| 0 | 0 \| 0 | 1 \| 0 | 0 \| 0 | 0 \| 0 | 0 \| 0 | 1 \| 0 | 19 \| 0 | 20 \| 4 | 88 \| 0 | 18 \| 1 |
|  | AUB17 | 1 \| 0 | 0 \| 0 | 0 \| 0 | 0 \| 0 | 0 \| 0 | 6 \| 1 | 0 \| 0 | 0 \| 0 | 80 \| 53 | 0 \| 0 | 0 \| 0 | 0 \| 0 | 0 \| 0 | 67 \| 0 | 23 \| 2 | 131 \| 0 | 59 \| 51 |
|  | SOU17 | 0 \| 0 | 0 \| 0 | 0 \| 0 | 0 \| 0 | 0 \| 0 | 13 \| 6 | 1 \| 0 | 0 \| 0 | 9 \| 2 | 0 \| 0 | 0 \| 0 | 0 \| 0 | 0 \| 0 | 576 \| 0 | 68 \| 18 | 1110 \| 0 | 6 \| 1 |
|  | SMH16 | 0 \| 0 | 0 \| 0 | 0 \| 0 | 0 \| 0 | 0 \| 0 | 117 \| 111 | 0 \| 0 | 0 \| 0 | 20 \| 4 | 0 \| 0 | 0 \| 0 | 0 \| 0 | 0 \| 0 | 43 \| 0 | 277 \| 125 | 400 \| 0 | 27 \| 0 |
|  | SMH17 | 12 \| 0 | 0 \| 0 | 0 \| 0 | 0 \| 0 | 1 \| 0 | 13 \| 2 | 0 \| 0 | 0 \| 0 | 8 \| 0 | 0 \| 0 | 0 \| 0 | 0 \| 0 | 0 \| 0 | 19 \| 0 | 431 \| 163 | 1012 \| 0 | 7 \| 2 |
|  | MULTI | 13 \| 0 | 0 \| 0 | 0 \| 0 | 0 \| 0 | 0 \| 0 | 0 \| 0 | 0 \| 0 | 0 \| 0 | 16 \| 14 | 0 \| 0 | 0 \| 0 | 5 \| 0 | 1 \| 0 | 0 \| 0 | 70 \| 1 | 0 \| 0 | 22 \| 0 |

## S14 Table: Number of significant markers in Het2

Number of significant markers identified for each trait and each environment in Het1. The first and second value are respectively for an FDR levels of 0.2 and 0.05. JAR16, AUB17, SOU17, SMH16 and SMH17 indicate for Gso-MONO models. MULTI indicates for Gso-MULTI model

## S15 Table: Description of representative markers

|  | **Markers** | **A** | | **B** | | **C** | | **D** | | **E** | | **F** | |
| --- | --- | --- | --- | --- | --- | --- | --- | --- | --- | --- | --- | --- | --- |
|  |  | AX-91769326 | | AX-90974317 | | AX-91747122 | | AX-90921960 | | AX-90654883 | | AX-91003160 | |
|  | **Chromosome** | 8 | | 5 | | 8 | | 5 | | 1 | | 6 | |
|  | **Physical position (Mbp)** | 130 | | 205 | | 9,3 | | 5,1 | | 18,2 | | 102 | |
|  | $\boldsymbol{p}_{\boldsymbol{D}}$ | 0,31 | | 0,28 | | 0,38 | | 0,54 | | 0,56 | | 0,37 | |
|  | $\boldsymbol{p}_{\boldsymbol{F}}$ | 0,38 | | 0,53 | | 0,53 | | 0,31 | | 0,39 | | 0,45 | |
|  |  | **-log(Pvalue)** | **Effect** | **-log(Pvalue)** | **Effect** | **-log(Pvalue)** | **Effect** | **-log(Pvalue)** | **Effect** | **-log(Pvalue)** | **Effect** | **-log(Pvalue)** | **Effect** |
| **Gso** | $\boldsymbol{g}_{\boldsymbol{Het}\boldsymbol{2}}$ | 3,435 | - | 3,453 | - | 4,204 | - | 2,994 | - | 1,626 | - | 5,201 | - |
|  | $\boldsymbol{g}_{\boldsymbol{Het}\boldsymbol{1}}$ | 1,883 | - | 2,004 | - | 0,548 | - | 4,72 | - | 0,278 | - | 0,472 | - |
|  | $\boldsymbol{a}$ | 5,467 | -1,466 | 0,692 | -0,395 | 1,632 | 0,728 | 0,566 | -0,39 | 0,668 | -0,369 | 0,8 | -0,44 |
|  | $\boldsymbol{a}_{\boldsymbol{DD}}$ | 2,178 | -1,618 | 0,556 | -0,593 | 5,516 | 2,617 | 0,22 | 0,263 | 0,009 | -0,013 | 2,114 | -1,391 |
|  | $\boldsymbol{a}_{\boldsymbol{DF}}$ | 2,905 | -1,401 | 0,614 | -0,464 | 0,253 | 0,195 | 0,436 | -0,364 | 0,625 | -0,444 | 0,001 | -0,001 |
|  | $\boldsymbol{a}_{\boldsymbol{FF}}$ | 2,113 | -1,379 | 0,093 | -0,128 | 0,496 | -0,626 | 0,798 | -1,069 | 0,67 | -0,649 | 0,045 | 0,073 |
|  | $\boldsymbol{s}_{\boldsymbol{a}}$ | 0,115 | -0,239 | 0,266 | -0,465 | 4,013 | 3,243 | 0,827 | 1,332 | 0,432 | 0,636 | 1,185 | -1,464 |
|  | $\boldsymbol{t}_{\boldsymbol{a}}$ | 0,068 | 0,097 | 0,082 | -0,104 | 0,972 | -0,801 | 0,026 | 0,039 | 0,099 | -0,113 | 0,769 | 0,658 |
|  | $\boldsymbol{d}$ | 0,987 | 0,582 | 6,055 | 1,663 | 0,262 | -0,217 | 2,213 | 1,056 | 0,339 | 0,255 | 3,502 | 1,228 |
|  | $\boldsymbol{d}_{\boldsymbol{DD}}$ | 0,506 | 0,677 | 3,607 | 2,376 | 0,52 | -0,758 | 0,194 | -0,281 | 0,866 | -0,886 | 1,775 | 1,518 |
|  | $\boldsymbol{d}_{\boldsymbol{DF}}$ | 0,426 | -0,458 | 2,482 | -1,436 | 0,496 | 0,403 | 5,946 | -2,51 | 0,127 | -0,155 | 0,282 | 0,27 |
|  | $\boldsymbol{d}_{\boldsymbol{FF}}$ | 0,491 | 0,612 | 1,281 | 1,176 | 0,318 | 0,51 | 0,612 | 0,939 | 1,614 | 1,497 | 3,49 | 2,435 |
|  | $\boldsymbol{s}_{\boldsymbol{d}}$ | 0,026 | 0,065 | 0,755 | 1,201 | 0,644 | -1,268 | 0,663 | -1,22 | 2,142 | -2,383 | 0,496 | -0,916 |
|  | $\boldsymbol{t}_{\boldsymbol{d}}$ | 0,106 | -0,187 | 0,217 | -0,34 | 0,174 | -0,279 | 2,637 | 2,181 | 0,089 | -0,151 | 3,396 | -2,247 |
|  | $\boldsymbol{\Delta}_{\boldsymbol{LD}}$ | 0,086 | 0,084 | 0,348 | 0,286 | 0,762 | -0,463 | 0,753 | 0,585 | 0,286 | -0,243 | 1,02 | 0,559 |
|  | $\boldsymbol{o}_{\boldsymbol{a}}$ | 0,883 | 0,58 | 0,229 | -0,196 | 3,613 | 1,498 | 1,037 | 0,74 | 4,271 | 1,386 | 0,276 | 0,238 |
|  | $\boldsymbol{o}_{\boldsymbol{d}}$ | 0,02 | 0,03 | 0,041 | 0,053 | 0,284 | -0,303 | 1,131 | -0,969 | 0,11 | 0,127 | 6,607 | 2,48 |
| **Gad** | $\boldsymbol{\alpha}$ | 6,2 | -1,517 | 0,744 | -0,368 | 0,656 | 0,35 | 0,104 | -0,081 | 0,557 | -0,323 | 1,444 | -0,62 |
|  | $\boldsymbol{\delta}$ | 1,027 | 0,568 | 6,861 | 1,58 | 0,292 | -0,207 | 2,434 | 0,955 | 0,13 | 0,109 | 1,555 | 0,705 |

Markers A and B represent QTLs identified with both Gad and Gso models, respectively for additivity and dominance. Markers C and D represent background specific effects in Gso model with no effect in Gad model. Markers E and F represent the origin effect on Gso model. Markers A, C and E represent additive effect. Markers B, D and F represent dominance effect. The frequencies $p_{D}$ and $p_{F}$ are respectively the SNP allelic frequencies in the Dent and the Flint group. Effect indicates the estimated value of the contrast tests.

## S16 Table. Repartition of partial and overdominance markers.

| **Panel** | **Trait** | **Env** | **Contrast** | **Nb of significant dominance QTLs** | **% of overdominance QTLs** | **Ratio \|d/a\|** | | | |
| --- | --- | --- | --- | --- | --- | --- | --- | --- | --- |
|  |  |  |  |  |  | **Min** | **Max** | **Mean** | **Sd** |
| Het2 | FloF | JAR16 | $\boldsymbol{d}$ | 2 | 100 | 2,06 | 3,93 | 3 | 1,32 |
|  |  | JAR16 | $\boldsymbol{d}_{\boldsymbol{DD}}$ | 11 | 100 | 1,71 | 12,3 | 4,32 | 4,19 |
|  |  | JAR16 | $\boldsymbol{d}_{\boldsymbol{DF}}$ | 1 | 100 | 2,87 | 2,87 | 2,87 | - |
|  |  | JAR16 | $\boldsymbol{d}_{\boldsymbol{FF}}$ | 1 | 100 | 2,08 | 2,08 | 2,08 | - |
|  |  | AUB17 | $\boldsymbol{d}_{\boldsymbol{DD}}$ | 29 | 100 | 1,17 | 18,5 | 3,54 | 3,01 |
|  |  | AUB17 | $\boldsymbol{d}_{\boldsymbol{DF}}$ | 27 | 100 | 1,08 | 63 | 18,4 | 15,3 |
|  |  | SOU17 | $\boldsymbol{d}$ | 11 | 100 | 2,29 | 5,92 | 3,63 | 1,29 |
|  |  | SOU17 | $\boldsymbol{d}_{\boldsymbol{DD}}$ | 24 | 100 | 1,69 | 10,6 | 4,91 | 2,66 |
|  |  | SOU17 | $\boldsymbol{d}_{\boldsymbol{DF}}$ | 15 | 100 | 1,5 | 50,1 | 6,95 | 12,1 |
|  |  | SOU17 | $\boldsymbol{d}_{\boldsymbol{FF}}$ | 13 | 100 | 2,08 | 8,83 | 5,09 | 2,94 |
|  |  | SMH16 | $\boldsymbol{d}$ | 2 | 100 | 14,9 | 47,6 | 31,2 | 23,1 |
|  |  | SMH16 | $\boldsymbol{d}_{\boldsymbol{DD}}$ | 32 | 100 | 1,55 | 46,3 | 27,9 | 13,6 |
|  |  | SMH16 | $\boldsymbol{d}_{\boldsymbol{DF}}$ | 5 | 100 | 1,96 | 349 | 73,6 | 154 |
|  |  | SMH16 | $\boldsymbol{d}_{\boldsymbol{FF}}$ | 2 | 100 | 8,07 | 49,5 | 28,8 | 29,3 |
|  |  | SMH17 | $\boldsymbol{d}$ | 4 | 100 | 2,18 | 3,06 | 2,52 | 0,38 |
|  |  | SMH17 | $\boldsymbol{d}_{\boldsymbol{DD}}$ | 119 | 100 | 1,04 | 147 | 6,6 | 23 |
|  |  | SMH17 | $\boldsymbol{d}_{\boldsymbol{DF}}$ | 14 | 100 | 1,59 | 261 | 34,5 | 73,7 |
|  |  | SMH17 | $\boldsymbol{d}_{\boldsymbol{FF}}$ | 39 | 100 | 1,28 | 123 | 10,7 | 19,8 |
|  |  | MULTI | $\boldsymbol{d}_{\boldsymbol{DD}}$ | 31 | 100 | 1,35 | 7,54 | 1,94 | 1,23 |
|  |  | MULTI | $\boldsymbol{d}_{\boldsymbol{DF}}$ | 236 | 100 | 0,95 | 1040 | 10,6 | 67,4 |
|  |  | MULTI | $\boldsymbol{d}_{\boldsymbol{FF}}$ | 1 | 100 | 2,62 | 2,62 | 2,62 | - |
|  | FloM | JAR16 | $\boldsymbol{d}_{\boldsymbol{DF}}$ | 9 | 100 | 1,98 | 10,1 | 4,26 | 3,33 |
|  |  | AUB17 | $\boldsymbol{d}$ | 7 | 100 | 15,9 | 76,4 | 34,6 | 19,2 |
|  |  | AUB17 | $\boldsymbol{d}_{\boldsymbol{DD}}$ | 1 | 100 | 3,3 | 3,3 | 3,3 | - |
|  |  | AUB17 | $\boldsymbol{d}_{\boldsymbol{DF}}$ | 67 | 100 | 1,66 | 57,8 | 8,45 | 7,96 |
|  |  | SOU17 | $\boldsymbol{d}_{\boldsymbol{DF}}$ | 11 | 100 | 1,03 | 42,3 | 7,88 | 11,7 |
|  |  | SOU17 | $\boldsymbol{d}_{\boldsymbol{FF}}$ | 2 | 100 | 24,3 | 24,3 | 24,3 | 0 |
|  |  | SMH16 | $\boldsymbol{d}$ | 1 | 100 | 97,3 | 97,3 | 97,3 | - |
|  |  | SMH16 | $\boldsymbol{d}_{\boldsymbol{DD}}$ | 4 | 100 | 2,48 | 2,48 | 2,48 | 0 |
|  |  | SMH16 | $\boldsymbol{d}_{\boldsymbol{DF}}$ | 6 | 100 | 1,61 | 16,8 | 9,66 | 5,79 |
|  |  | SMH17 | $\boldsymbol{d}_{\boldsymbol{DD}}$ | 2 | 100 | 1,64 | 2,23 | 1,94 | 0,42 |
|  |  | MULTI | $\boldsymbol{d}_{\boldsymbol{DD}}$ | 9 | 100 | 1,49 | 12,9 | 3,58 | 3,58 |
|  |  | MULTI | $\boldsymbol{d}_{\boldsymbol{DF}}$ | 123 | 100 | 1,24 | 9,32 | 7,08 | 1,45 |
|  | Hum | JAR16 | $\boldsymbol{d}$ | 8 | 100 | 4,14 | 10,1 | 4,89 | 2,09 |
|  |  | JAR16 | $\boldsymbol{d}_{\boldsymbol{DD}}$ | 1 | 100 | 8,14 | 8,14 | 8,14 | - |
|  |  | JAR16 | $\boldsymbol{d}_{\boldsymbol{FF}}$ | 25 | 100 | 1,38 | 77,9 | 4,98 | 15,4 |
|  |  | AUB17 | $\boldsymbol{d}$ | 2 | 100 | 2,47 | 4,35 | 3,41 | 1,32 |
|  |  | AUB17 | $\boldsymbol{d}_{\boldsymbol{DD}}$ | 29 | 100 | 1,39 | 330 | 21,8 | 75,1 |
|  |  | AUB17 | $\boldsymbol{d}_{\boldsymbol{FF}}$ | 2 | 100 | 5,44 | 23,2 | 14,3 | 12,5 |
|  |  | SOU17 | $\boldsymbol{d}$ | 1 | 100 | 20,2 | 20,2 | 20,2 | - |
|  |  | SOU17 | $\boldsymbol{d}_{\boldsymbol{DD}}$ | 2 | 100 | 1,82 | 4,1 | 2,96 | 1,62 |
|  |  | SOU17 | $\boldsymbol{d}_{\boldsymbol{FF}}$ | 2 | 100 | 3,53 | 6,25 | 4,89 | 1,92 |
|  |  | SMH16 | $\boldsymbol{d}$ | 1 | 100 | 4,82 | 4,82 | 4,82 | - |
|  |  | SMH16 | $\boldsymbol{d}_{\boldsymbol{DD}}$ | 1 | 100 | 3,64 | 3,64 | 3,64 | - |
|  |  | SMH16 | $\boldsymbol{d}_{\boldsymbol{FF}}$ | 6 | 100 | 1,2 | 4,69 | 2,18 | 1,27 |
|  |  | SMH17 | $\boldsymbol{d}$ | 22 | 100 | 1,26 | 91,4 | 6,07 | 19,1 |
|  |  | SMH17 | $\boldsymbol{d}_{\boldsymbol{FF}}$ | 72 | 100 | 1,06 | 216 | 9,72 | 26,2 |
|  |  | MULTI | $\boldsymbol{d}_{\boldsymbol{DD}}$ | 8 | 100 | 5,11 | 5,11 | 5,11 | 0 |
|  | GY | JAR16 | $\boldsymbol{d}_{\boldsymbol{DF}}$ | 4 | 100 | 2,42 | 5,94 | 3,67 | 1,59 |
|  |  | AUB17 | $\boldsymbol{d}_{\boldsymbol{DF}}$ | 107 | 100 | 1,24 | 3,39 | 2,15 | 0,455 |
|  |  | SOU17 | $\boldsymbol{d}$ | 1 | 100 | 211 | 211 | 211 | - |
|  |  | SOU17 | $\boldsymbol{d}_{\boldsymbol{DF}}$ | 78 | 100 | 1,42 | 111 | 22,1 | 20,6 |
|  |  | SMH16 | $\boldsymbol{d}_{\boldsymbol{DF}}$ | 28 | 100 | 1,48 | 12,6 | 4,02 | 3,44 |
|  |  | SMH17 | $\boldsymbol{d}_{\boldsymbol{DF}}$ | 9 | 100 | 5,13 | 7,46 | 5,57 | 0,712 |
|  |  | MULTI | $\boldsymbol{d}_{\boldsymbol{DF}}$ | 16 | 100 | 2,41 | 7,17 | 6,47 | 1,14 |
| Het1 | FloF | AUB15 | $\boldsymbol{d}_{\boldsymbol{DF}}$ | 5 | 100 | 1,41 | 6 | 3,14 | 1,7 |
|  |  | MOR14 | $\boldsymbol{d}_{\boldsymbol{DF}}$ | 2 | 100 | 2,51 | 3,05 | 2,78 | 0,382 |
|  |  | VIL15 | $\boldsymbol{d}_{\boldsymbol{DF}}$ | 21 | 100 | 1,74 | 64,2 | 4,71 | 13,6 |
|  |  | SMH14 | $\boldsymbol{d}_{\boldsymbol{DF}}$ | 2 | 100 | 1,29 | 3,45 | 2,37 | 1,53 |
|  |  | SMH15 | $\boldsymbol{d}_{\boldsymbol{DF}}$ | 1 | 100 | 7,73 | 7,73 | 7,73 | - |
|  |  | CAU14 | $\boldsymbol{d}_{\boldsymbol{DF}}$ | 13 | 100 | 1,8 | 3,76 | 2,1 | 0,542 |
|  |  | CON15 | $\boldsymbol{d}_{\boldsymbol{DF}}$ | 5 | 100 | 1,11 | 28,9 | 7,92 | 11,9 |
|  | FloM | MOR14 | $\boldsymbol{d}_{\boldsymbol{DF}}$ | 3 | 100 | 1,11 | 1,61 | 1,33 | 0,255 |
|  |  | SMH14 | $\boldsymbol{d}_{\boldsymbol{DF}}$ | 1 | 100 | 2,94 | 2,94 | 2,94 | - |
|  |  | CAU14 | $\boldsymbol{d}_{\boldsymbol{DF}}$ | 13 | 100 | 1,07 | 3,16 | 1,8 | 0,527 |
|  | Hum | VIL15 | $\boldsymbol{d}_{\boldsymbol{DF}}$ | 1 | 100 | 14,6 | 14,6 | 14,6 | - |
|  |  | SMH14 | $\boldsymbol{d}_{\boldsymbol{DF}}$ | 3 | 100 | 1,15 | 1,79 | 1,58 | 0,37 |
|  |  | SMH15 | $\boldsymbol{d}_{\boldsymbol{DF}}$ | 6 | 100 | 1,19 | 1,7 | 1,33 | 0,204 |
|  | GY | AUB15 | $\boldsymbol{d}_{\boldsymbol{DF}}$ | 12 | 100 | 1,07 | 23,1 | 3,47 | 6,2 |
|  |  | VIL15 | $\boldsymbol{d}_{\boldsymbol{DF}}$ | 3 | 100 | 1,8 | 1,8 | 1,8 | 0 |
|  |  | SMH14 | $\boldsymbol{d}_{\boldsymbol{DF}}$ | 13 | 100 | 1,49 | 13,7 | 3,37 | 4,59 |
|  |  | SMH15 | $\boldsymbol{d}_{\boldsymbol{DF}}$ | 2 | 100 | 2,69 | 2,91 | 2,8 | 0,154 |
|  |  | CON15 | $\boldsymbol{d}_{\boldsymbol{DF}}$ | 1 | 100 | 2,01 | 2,01 | 2,01 | - |

Number of significant dominance QTLs for each panel, trait and environment. The percentage of overdominance QTLs is the proportion of dominance QTLs with a ratio |d/a| superior to 1. The FDR nominal level is 0.05.

## S17 Table: QTL stability across environments in Het1

| **Contrast** | **Trait** | **Tot** | **Gso-MULTI only** | **Gso-MULTI and Gso-MONO** | | | | | | | **Gso-MONO only** | | | | | | |
| --- | --- | --- | --- | --- | --- | --- | --- | --- | --- | --- | --- | --- | --- | --- | --- | --- | --- |
|  |  |  |  | **1** | **2** | **3** | **4** | **5** | **6** | **7** | **1** | **2** | **3** | **4** | **5** | **6** | **7** |
| Additivity | FloF | 3 | 0 | 0 | 1 | 0 | 0 | 0 | 0 | 0 | 2 | 0 | 0 | 0 | 0 | 0 | 0 |
|  | FloM | 8 | 0 | 0 | 1 | 0 | 0 | 0 | 1 | 0 | 6 | 0 | 0 | 0 | 0 | 0 | 0 |
|  | Hum | 19 | 0 | 0 | 0 | 0 | 0 | 0 | 0 | 0 | 19 | 0 | 0 | 0 | 0 | 0 | 0 |
|  | GY | 4 | 0 | 0 | 0 | 0 | 0 | 0 | 0 | 0 | 4 | 0 | 0 | 0 | 0 | 0 | 0 |
| Dominance | FloF | 1 | 0 | 0 | 0 | 0 | 0 | 0 | 0 | 0 | 1 | 0 | 0 | 0 | 0 | 0 | 0 |
|  | FloM | 0 | 0 | 0 | 0 | 0 | 0 | 0 | 0 | 0 | 0 | 0 | 0 | 0 | 0 | 0 | 0 |
|  | Hum | 1 | 0 | 0 | 0 | 0 | 0 | 0 | 0 | 0 | 1 | 0 | 0 | 0 | 0 | 0 | 0 |
|  | GY | 0 | 0 | 0 | 0 | 0 | 0 | 0 | 0 | 0 | 0 | 0 | 0 | 0 | 0 | 0 | 0 |

“Contrast” terms correspond respectively to the Additivity ($a_{DF})$ and the Dominance ($d_{DF}$) QTLs. “Gso-MONO” counts for the number of times a QTL has been observed in single environments models. “Gso-MULTI and Gso-MONO” counts for the number of times a QTL has been observed in single environment and multiple environment models. “Gso-MULTI” only refers to QTLs only identified with multiple environment model. “Total” indicates the total number of QTLs. The nominal FDR level is fixed at 0.05.

## S18 Table QTL stability across environments in Het2.

| **Contrasts** | **Trait** | **Total** | **Gso-MULTI**  **only** | **Gso-MULTI and Gso-MONO** | | | | | **Gso-MONO only** | | | | |
| --- | --- | --- | --- | --- | --- | --- | --- | --- | --- | --- | --- | --- | --- |
|  |  |  |  | **1** | **2** | **3** | **4** | **5** | **1** | **2** | **3** | **4** | **5** |
| Additivity | FloF | 11 | 5 | 1 | 1 | 0 | 0 | 0 | 4 | 0 | 0 | 0 | 0 |
|  | FloM | 13 | 0 | 1 | 1 | 1 | 0 | 0 | 9 | 1 | 0 | 0 | 0 |
|  | Hum | 18 | 0 | 1 | 0 | 0 | 0 | 0 | 15 | 2 | 0 | 0 | 0 |
| Dominance | FloF | 9 | 7 | 0 | 0 | 0 | 0 | 0 | 2 | 0 | 0 | 0 | 0 |
|  | FloM | 12 | 1 | 1 | 0 | 0 | 0 | 0 | 10 | 0 | 0 | 0 | 0 |
|  | Hum | 2 | 0 | 0 | 0 | 0 | 0 | 0 | 2 | 0 | 0 | 0 | 0 |
|  | GY | 7 | 1 | 0 | 0 | 0 | 0 | 0 | 6 | 0 | 0 | 0 | 0 |
| Additivity Origin | FloM | 29 | 0 | 1 | 1 | 0 | 0 | 1 | 23 | 2 | 1 | 0 | 0 |
|  | GY | 8 | 0 | 0 | 0 | 0 | 0 | 0 | 8 | 0 | 0 | 0 | 0 |
| Dominance Origin | FloF | 3 | 0 | 0 | 0 | 0 | 0 | 0 | 3 | 0 | 0 | 0 | 0 |
|  | GY | 15 | 0 | 1 | 0 | 0 | 0 | 0 | 14 | 0 | 0 | 0 | 0 |

QTLs detected with the MULTI and the different MONO models were merged if their confidence intervals overlapped. “Contrast” refers to QTLs detected for Additivity ($a$, $a_{DD}$, $a_{DF}$, $a_{FF}$, $s_{a}$ and $t_{a})$, Dominance ($d$, $d_{DD}$, $d_{DF}$, $d_{FF}$, $s_{d}$ and $t_{d}$) and the Additivity Origin ($o_{a}$) and the Dominance Origin ($o_{d}$) QTLs. “Gso-MONO only” counts the number of times a QTL was observed in single environments. “Gso-MULTI and Gso-MONO” counts the number of times a QTL has been observed in single environment and in the multiple environment analysis. “Gso-MULTI” refers to QTLs only identified with multiple environment model. “Total” indicates the total number of QTLs. The nominal FDR level is fixed at 0.05.

## S19 Table: Comparison of the number of QTLs identified in Het1, in Het2 or in both panels.

| **Trait** | **Contrast** | **Total** | **Het1 only** | **Both** | **Het2 only** |
| --- | --- | --- | --- | --- | --- |
| FloF | $a_{DF}$ | 117 | 37 | 18 | 62 |
|  | $d_{DF}$ | 61 | 16 | 3 | 42 |
|  | $\Delta_{LD}$ | 40 | 0 | 0 | 40 |
|  | $g_{Het1}$ | 120 | 16 | 21 | 83 |
| FloM | $a_{DF}$ | 68 | 27 | 7 | 34 |
|  | $d_{DF}$ | 23 | 8 | 0 | 15 |
|  | $\Delta_{LD}$ | 24 | 5 | 3 | 16 |
|  | $g_{Het1}$ | 50 | 23 | 2 | 25 |
| Hum | $a_{DF}$ | 71 | 55 | 5 | 11 |
|  | $d_{DF}$ | 7 | 7 | 0 | 0 |
|  | $\Delta_{LD}$ | 6 | 1 | 0 | 5 |
|  | $g_{Het1}$ | 40 | 38 | 0 | 2 |
| GY | $a_{DF}$ | 22 | 22 | 0 | 0 |
|  | $d_{DF}$ | 40 | 9 | 4 | 27 |
|  | $\Delta_{LD}$ | 10 | 7 | 0 | 3 |
|  | $g_{Het1}$ | 46 | 22 | 1 | 23 |

QTLs detected with the MULTI model and the MONO model *in* different environments, were merged if their confidence intervals overlapped. Only $a_{DF}$, $d_{DF}$,$\Delta_{LD}$ and $g_{Het1}$ QTLs are strictly comparable between both hybrid panels. QTLs were merged if their confidence intervals overlapped. “Total” is the sum of all QTLs identified in at least one panel, at a nominal FDR level of 0.2.

## S20 Table. Correlation of additive effect between Gad and Gso.

| **Panel** | **Trait** | **Env** | $\boldsymbol{cor}\left( \boldsymbol{a,\alpha} \right)$ | $\boldsymbol{cor}\left( \boldsymbol{d,\delta} \right)$ | $\boldsymbol{cor}\left( \boldsymbol{Pval}_{\boldsymbol{a}}\boldsymbol{,}\boldsymbol{Pval}_{\boldsymbol{\alpha}} \right)$ | $\boldsymbol{cor}\left( \boldsymbol{Pval}_{\boldsymbol{d}}\boldsymbol{,}\boldsymbol{Pval}_{\boldsymbol{\delta}} \right)$ |
| --- | --- | --- | --- | --- | --- | --- |
| Het1 | FloF | AUB15 | 0.998 | 0.939 | 0.992 | 0.852 |
|  |  | MOR14 | 0.997 | 0.946 | 0.989 | 0.867 |
|  |  | VIL15 | 0.997 | 0.942 | 0.991 | 0.848 |
|  |  | SMH14 | 0.997 | 0.944 | 0.99 | 0.86 |
|  |  | SMH15 | 0.997 | 0.943 | 0.991 | 0.861 |
|  |  | CAU14 | 0.997 | 0.946 | 0.989 | 0.864 |
|  |  | CON15 | 0.998 | 0.945 | 0.992 | 0.86 |
|  |  | MULTI | 0.995 | 0.928 | 0.987 | 0.851 |
|  | FloM | AUB15 | 0.998 | 0.944 | 0.992 | 0.86 |
|  |  | MOR14 | 0.998 | 0.941 | 0.992 | 0.854 |
|  |  | VIL15 | 0.998 | 0.942 | 0.992 | 0.85 |
|  |  | SMH14 | 0.998 | 0.947 | 0.991 | 0.863 |
|  |  | SMH15 | 0.998 | 0.946 | 0.992 | 0.862 |
|  |  | CAU14 | 0.997 | 0.946 | 0.99 | 0.865 |
|  |  | CON15 | 0.997 | 0.943 | 0.991 | 0.856 |
|  |  | MULTI | 0.996 | 0.93 | 0.988 | 0.856 |
|  | Hum | AUB15 | 0.997 | 0.938 | 0.989 | 0.846 |
|  |  | MOR14 | 0.998 | 0.941 | 0.992 | 0.852 |
|  |  | VIL15 | 0.998 | 0.936 | 0.992 | 0.841 |
|  |  | SMH14 | 0.997 | 0.944 | 0.991 | 0.858 |
|  |  | SMH15 | 0.998 | 0.941 | 0.994 | 0.852 |
|  |  | CAU14 | 0.998 | 0.939 | 0.993 | 0.846 |
|  |  | CON15 | 0.998 | 0.944 | 0.993 | 0.855 |
|  |  | MULTI | 0.993 | 0.93 | 0.984 | 0.849 |
|  | GY | AUB15 | 0.998 | 0.934 | 0.994 | 0.835 |
|  |  | MOR14 | 0.999 | 0.939 | 0.995 | 0.848 |
|  |  | VIL15 | 0.998 | 0.935 | 0.994 | 0.833 |
|  |  | SMH14 | 0.999 | 0.946 | 0.995 | 0.855 |
|  |  | SMH15 | 0.999 | 0.942 | 0.996 | 0.86 |
|  |  | CAU14 | 0.999 | 0.945 | 0.995 | 0.852 |
|  |  | CON15 | 0.999 | 0.936 | 0.994 | 0.843 |
|  |  | MULTI | 0.997 | 0.933 | 0.991 | 0.848 |
| Het2 | FloF | AUB15 | 0.998 | 0.939 | 0.992 | 0.852 |
|  |  | MOR14 | 0.997 | 0.943 | 0.99 | 0.861 |
|  |  | VIL15 | 0.997 | 0.942 | 0.991 | 0.848 |
|  |  | SMH14 | 0.997 | 0.946 | 0.991 | 0.859 |
|  |  | SMH15 | 0.997 | 0.943 | 0.991 | 0.861 |
|  |  | CAU14 | 0.996 | 0.948 | 0.989 | 0.866 |
|  |  | CON15 | 0.998 | 0.945 | 0.992 | 0.86 |
|  |  | MULTI | 0.995 | 0.928 | 0.987 | 0.851 |
|  | FloM | AUB15 | 0.998 | 0.944 | 0.992 | 0.86 |
|  |  | MOR14 | 0.998 | 0.941 | 0.992 | 0.854 |
|  |  | VIL15 | 0.998 | 0.942 | 0.992 | 0.85 |
|  |  | SMH14 | 0.998 | 0.947 | 0.991 | 0.863 |
|  |  | SMH15 | 0.998 | 0.946 | 0.992 | 0.862 |
|  |  | CAU14 | 0.997 | 0.946 | 0.99 | 0.865 |
|  |  | CON15 | 0.997 | 0.943 | 0.991 | 0.856 |
|  |  | MULTI | 0.996 | 0.93 | 0.988 | 0.856 |
|  | Hum | AUB15 | 0.997 | 0.938 | 0.989 | 0.846 |
|  |  | MOR14 | 0.998 | 0.941 | 0.992 | 0.852 |
|  |  | VIL15 | 0.998 | 0.936 | 0.992 | 0.841 |
|  |  | SMH14 | 0.997 | 0.944 | 0.991 | 0.858 |
|  |  | SMH15 | 0.998 | 0.941 | 0.994 | 0.852 |
|  |  | CAU14 | 0.998 | 0.939 | 0.993 | 0.846 |
|  |  | CON15 | 0.998 | 0.944 | 0.993 | 0.855 |
|  |  | MULTI | 0.993 | 0.93 | 0.984 | 0.849 |
|  | GY | AUB15 | 0.998 | 0.934 | 0.994 | 0.835 |
|  |  | MOR14 | 0.999 | 0.939 | 0.995 | 0.848 |
|  |  | VIL15 | 0.998 | 0.935 | 0.994 | 0.833 |
|  |  | SMH14 | 0.999 | 0.946 | 0.995 | 0.855 |
|  |  | SMH15 | 0.999 | 0.942 | 0.996 | 0.86 |
|  |  | CAU14 | 0.999 | 0.945 | 0.995 | 0.852 |
|  |  | CON15 | 0.999 | 0.936 | 0.994 | 0.843 |
|  |  | MULTI | 0.997 | 0.933 | 0.991 | 0.848 |

$cor\left( \alpha,a \right)$ (or $cor\left( \delta,d \right)$) indicates the correlation between additive effects $\alpha$ and a (or between dominance effects $\delta$ and d).$cor\left( {Pval}_{\alpha},{Pval}_{a} \right)$ indicates the correlation between additive Pvalues for effects $\alpha$ and a (and between dominance effects$\delta$ and d). All correlations are calculated over all candidate markers for each panel, trait and environment.
