# Supplementary material for: Identifying QTLs involved in hybrid performance and heterotic group complementarity: new GWAS models applied to factorial and admixed diallel maize hybrid panels
Source: Theor Appl Genet. 2023 Oct 10;136(11):219. doi: 10.1007/s00122-023-04431-w (PMC10564676; doi:10.1007/s00122-023-04431-w)
Supplement: Supplementary file 2 — (DOCX 20 KB) [file 122_2023_4431_MOESM2_ESM.docx]

# Supporting information

**S1 Appendix. Spatial correction model for Het2 by environment**

**S2 Appendix. Broad Sense Heritability**

**S3 Appendix. Variance estimation procedure in GWAS**

**S1 Fig. Estimation of the** LD **interval around a significant marker.** The LD interval estimation starts with the definition of an arbitrary interval of 0.5 cM on both sides of the significant marker. LD (Linkage Disequilibrium) is calculated as an R² between the significant marker and the other markers within each interval using genotypic information. Then, LD is used to estimate LD extent with a threshold of 0.1. If the LD extent value is superior to the size of the interval, the interval size is increased by 0.1 cM. The procedure is repeated from the LD calculation until the LD extent reaches a value inferior to the LD interval length. Lastly, the LD extent value is used to define the limits of the LD interval around the significant marker.

**S2 Fig.** Quantile-by-Quantile diagram of Pvalues for the Male Flowering **time (FloM) in JAR16 (Het2).** The black lines represent the ideal distribution of the Pvalue according to a uniform law.

**S3 Fig. Manhattan plot for the Male flowering time (FloM) in JAR16 (Het2) for the additive effect of the origin (**$\boldsymbol{o}_{\boldsymbol{a}}$**).** Blue and red lines represent the Pvalue threshold for FDR levels of 0.2 and 0.05.

**S4 Fig. Representation of QTLs for the Female flowering (FloF) and the grain moisture (Hum).** Rectangles represent the interval of QTLs. All results from the MONO and MULTI models were compilated for the Gso and Gad models. The FDR nominal level is 0.05.

**S5 Fig. Differences between Dent and Flint allelic frequencies for the detected dominance QTLs in Het2.** Violin plot of the difference of SNP allelic frequencies between the Dent and the Flint group calculated as $Diff=\left| p_{D}-p_{F} \right|$ with $p_{D}$ and $p_{F}$ the Dent and Flint frequencies. The FDR nominal level is 0.2.

**S6 Fig. Illustration of the variation of additive effect in the dent genetic background across environments.** Boxplot of phenotype corrected by the kinship according to the Gso genotypes of marker AX-91203539 on chromosome 1 for the grain yield (GY) in all Het2 environments (Colored boxplot; red, green and blue are for DD, DF and FF genetic backgrounds) and Gad model (Grey boxplot). The number at the bottom indicates the number of observations for each SO genotype.

**S1 Table. Contrast matrix for** $\boldsymbol{g}_{\boldsymbol{Het}\boldsymbol{1}}$ **effect (global QTL based on Het1 SO genotypes)** Contrast matrix defined to test a global effect of a QTL based on SO genotypes present in Het1 panel. Empty spaces indicate a weight of 0. With $C$ the matrix (defined above) associating weights to the fixed parameters of the model, one can test: $H_{0}:\left\{ C\beta=0 \right\}$ vs $H_{1}:\left\{ C\beta\neq0 \right\}$.

**S2 Table. Contrast matrix for** $\boldsymbol{g}_{\boldsymbol{Het}\boldsymbol{2}}$ **effect (global QTL based on Het2 SO genotypes).** Contrast matrix defined to test a global effect of a QTL based on SO genotypes present in the Het2 panel. Empty spaces indicate a weight of 0. With $C$ the matrix (defined above) associating weights to the fixed parameters of the model, one can test: $H_{0}:\left\{ C\beta=0 \right\}$ vs $H_{1}:\left\{ C\beta\neq0 \right\}$.

**S3 Table. Summary of agronomic traits with single environment models (MONO models)****.** Min, Max, Mean, Std.Dev are the minimum, maximum, mean and standard deviation of corrected phenotypic values. The variances $\sigma_{G}^{2}$ and $\sigma_{\varepsilon}^{2}$ are respectively for genetic and error effects.$H_{s}^{2}$ is the single environment heritability.

**S4 Table. Summary of agronomic traits with multiple environment models (MULTI model).** Min, Max, Mean, Std.Dev are the minimum, maximum, mean and standard deviation of corrected phenotypic values. “Mean with correction by the checks” is the phenotypic mean of all environments corrected by the value of the checks. The variances $\sigma_{G}^{2}$, $\sigma_{GE}^{2}$ and $\sigma_{\varepsilon}^{2}$ are respectively for genetic, genetic-by-environment interaction and error effects.$H_{M}^{2}$ is the multi-environment heritability.

**S5 Table. Single environment variance partition with MONO model. The** variances $\sigma_{a}^{2}$ , $\sigma_{d}^{2}$ , $\sigma_{aa}^{2}$ ,$\sigma_{ad}^{2}$ ,$\sigma_{dd}^{2}$ and $\sigma_{\varepsilon}^{2}$ are respectively for additivity, dominance, additive-by-additive epistasis, additive-by-dominance epistasis, dominance-by-dominance epistasis and error effects. The epistatic variance $\sigma_{I}^{2}$ is the sum of $\sigma_{aa}^{2}$ ,$\sigma_{ad}^{2}$ and$\sigma_{dd}^{2}$ variances. V(G) is the genetic variance defined as $V\left( G \right)= \sigma_{a}^{2}+\sigma_{d}^{2}+\sigma_{aa}^{2}+\sigma_{ad}^{2}+\sigma_{dd}^{2}$.

**S6 Table. Multi environment variance partition (MULTI model) in Het1.** The variances $\sigma_{a}^{2}$ , $\sigma_{d}^{2}$ , $\sigma_{aa}^{2}$ ,$\sigma_{ad}^{2}$ ,$\sigma_{dd}^{2}$ and $\sigma_{\varepsilon}^{2}$ are respectively for additivity, dominance, additive-by-additive epistasis, additive-by-dominance epistasis, dominance-by-dominance epistasis and error effects. The variances $\sigma_{a(e)}^{2}$, $\sigma_{d(e)}^{2}$ and $\sigma_{\varepsilon(e)}^{2}$ are environment-specific variances for the environment *e*.

**S7 Table. Multi environment variance partition (MULTI model) in Het2.** The variances $\sigma_{a}^{2}$ , $\sigma_{d}^{2}$ , $\sigma_{aa}^{2}$ ,$\sigma_{ad}^{2}$ ,$\sigma_{dd}^{2}$ and $\sigma_{\varepsilon}^{2}$ are respectively for additivity, dominance, additive-by-additive epistasis, additive-by-dominance epistasis, dominance-by-dominance epistasis and error effects. The variances $\sigma_{a(e)}^{2}$, $\sigma_{d(e)}^{2}$ and $\sigma_{\varepsilon(e)}^{2}$ are environment-specific variances for the environment *e*.

**S8 Table. Synthetic table of multiple environment variance partition (MULTI model).** The variances $\sigma_{a}^{2}$ , $\sigma_{d}^{2}$ , $\sigma_{aa}^{2}$ ,$\sigma_{ad}^{2}$ ,$\sigma_{dd}^{2}$ and $\sigma_{\varepsilon}^{2}$ are respectively for additivity, dominance, additive-by-additive epistasis, additive-by-dominance epistasis, dominance-by-dominance epistasis and error effects. The variances $\sigma_{a(e)}^{2}$, $\sigma_{d(e)}^{2}$ and $\sigma_{\varepsilon(e)}^{2}$ are environment-specific variances for the environment *e*. V(AxE) is the average additive-by-environment interaction variance (calculated as $\frac{\sum_{e} \sigma_{a(e)}^{2}}{n_{e}}$). V(DxE) is the average dominance-by-environment interaction variance (calculated as $\frac{\sum_{e} \sigma_{d(e)}^{2}}{n_{e}}$). $V(\varepsilon xE)$ is the average environment-specific error variance (calculated as $\frac{\sum_{e} \sigma_{d(e)}^{2}}{n_{e}}$). The term $n_{e}$ refers to the number of environments. V(G) is the genetic variance of main effects defined as $V\left( G \right)= \sigma_{a}^{2}+\sigma_{d}^{2}+\sigma_{aa}^{2}+\sigma_{ad}^{2}+\sigma_{dd}^{2}$.

**S9 Table. Number of candidate markers in Het1 for model Gad.** Number of candidate markers according to the environments, the traits and the contrast tests after filtering on the minor allele frequency (MAF) and the number of individuals in each genotypic class. AUB14, MOR14, VIL15, SMH14, SMH15, CAU14 and CON15 indicate for Gso-MONO models. MULTI indicates the Gso-MULTI model.

**S10 Table. Number of candidate markers in Het2 for model Gad.** Number of candidate markers according to the environments, the traits and the contrast tests after filtering on the minor allele frequency (MAF) and the number of individuals in each genotypic class. JAR16, AUB17, SOU17, SMH16 and SMH17 indicate the Gso-MONO models. MULTI indicates the Gso-MULTI model.

**S11 Table. Number of candidate markers in Het1 for model Gso.** Number of candidate markers according to the environments, the traits and the contrast tests after filtering on the minor allele frequency (MAF) and the number of individuals in each genotypic class. AUB14, MOR14, VIL15, SMH14, SMH15, CAU14 and CON15 indicate the Gso-MONO models. MULTI indicates the Gso-MULTI model.

**S12 Table: Number of candidate markers in Het2 for model Gso.** Number of candidate markers according to the environments, the traits and the contrast tests after filtering on the minor allele frequency (MAF) and the number of individuals in each genotypic class. JAR16, AUB17, SOU17, SMH16 and SMH17 indicate the Gso-MONO models. MULTI indicates the Gso-MULTI model.

**S13 Table. Number of significant markers in Het1.** Number of significant markers identified for each trait and each environment in Het1. The first and second values are, respectively, for FDR levels of 0.2 and 0.05. CAU14, AUB14, CON15, MOR14, VIL15, SMH14 and SMH15 indicate for Gso-MONO models. MULTI indicates for Gso-MULTI model.

**S14 Table. Number of significant markers in Het2.** Number of significant markers identified for each trait and each environment in Het1. The first and second values are, respectively, for FDR levels of 0.2 and 0.05. JAR16, AUB17, SOU17, SMH16 and SMH17 indicate the Gso-MONO models. MULTI indicates the Gso-MULTI model.

**S15 Table. Description of representative markers.** Markers A and B represent QTLs identified with both Gad and Gso models, respectively, for additivity and dominance. Markers C and D represent within background effects in the Gso model with no effect in the Gad model. Markers E and F represent the origin effect in the Gso model. Markers A, C and E represent additive effects. Markers B, D and F represent the dominance effect. Effect indicates the estimated value of the contrast tests.

**S16 Table. Repartition of partial and overdominance markers.** Number of significant dominance QTLs for each panel, trait and environment. The percentage of overdominance QTLs is the proportion of dominance QTLs with a ratio |d/a| superior to 1. The nominal FDR level is fixed at 0.05.

**S17 Table. QTL stability across environments in Het1.** “Contrast” terms correspond respectively to the Additivity ($a_{DF})$ and the Dominance ($d_{DF}$) QTLs. “Gso-MONO” counts for the number of times a QTL has been observed in single environments models. “Gso-MULTI and Gso-MONO” count for the number of times a QTL has been observed in single environment and multiple environment models. “Gso-MULTI” only refers to QTLs only identified with the multiple environment model. “Total” indicates the total number of QTLs. The nominal FDR level is fixed at 0.05.

**S18 Table. QTL stability across environments in Het2.** QTLs detected with the MULTI and the different MONO models were merged if their QTL intervals overlapped. “Contrast” refers to QTLs detected for Additivity ($a$, $a_{DD}$, $a_{DF}$, $a_{FF}$, $s_{a}$ and $t_{a})$, Dominance ($d$, $d_{DD}$, $d_{DF}$, $d_{FF}$, $s_{d}$ and $t_{d}$) and the Additivity Origin ($o_{a}$) and the Dominance Origin ($o_{d}$) QTLs. “Gso-MONO only” counts the number of times a QTL was observed in single environments. “Gso-MULTI and Gso-MONO” count the number of times a QTL has been observed in single environment and in the multiple environment analysis. “Gso-MULTI” refers to QTLs only identified with the multiple environment model. “Total” indicates the total number of QTLs. The nominal FDR level is fixed at 0.05.

**S19 Table Comparison of the number of QTLs identified in Het1, in Het2 or in both panels.** QTLs detected with the MULTI model and the MONO model *in* different environments were merged if their QTL intervals overlapped. Only $a_{DF}$, $d_{DF}$,$\Delta_{LD}$ and $g_{Het1}$ QTLs are strictly comparable between both hybrid panels. QTLs were merged if their QTL intervals overlapped. “Total” is the sum of all QTLs identified in at least one panel at a nominal FDR level of 0.2.

**S20 Table. Correlation of additive effect between Gad and Gso**. $cor\left( \alpha,a \right)$ (or $cor\left( \delta,d \right)$) indicates the correlation between additive effects $\alpha$ and a (or between dominance effects $\delta$ and d).$cor\left( {Pval}_{\alpha},{Pval}_{a} \right)$ indicates the correlation between additive Pvalues for effects $\alpha$ and a (and between dominance effects$\delta$ and d). All correlations are calculated over all candidate markers for each panel, trait and environment.
